# *Teucrium montanum* L.—Unrecognized Source of Phenylethanoid Glycosides: Green Extraction Approach and Elucidation of Phenolic Compounds via NMR and UHPLC-HR MS/MS

**DOI:** 10.3390/antiox12111903

**Published:** 2023-10-24

**Authors:** Ana Mandura Jarić, Ana Čikoš, Marijana Pocrnić, Krunoslav Aladić, Stela Jokić, Danijela Šeremet, Aleksandra Vojvodić Cebin, Draženka Komes

**Affiliations:** 1 Department of Food Engineering, Faculty of Food Technology and Biotechnology, University of Zagreb, Pierotii St. 6, 10000 Zagreb, Croatia; ana.mandura@pbf.unizg.hr (A.M.J.); dseremet@pbf.hr (D.Š.); avojvodic@pbf.hr (A.V.C.); 2 NMR Centre, Ruđer Bošković Institute, Bijenička 54, 10000 Zagreb, Croatia; 3 Department of Chemistry, Faculty of Science, University of Zagreb, Horvatovac 102a, 10000 Zagreb, Croatia; mpocrnic@chem.pmf.hr; 4 Faculty of Food Technology, Josip Juraj Strossmayer University of Osijek, Franje Kuhača 20, 31000 Osijek, Croatia; krunoslav.aladic@ptfos.hr (K.A.); stela.jokic@ptfos.hr (S.J.)

**Keywords:** microwave-assisted extraction, phenylethanoid glycosides, polyphenols, subcritical water extraction, *Teucrium montanum* L.

## Abstract

Health-oriented preferences, a demand for innovative food concepts, and technological advances have greatly influenced changes in the food industry and led to remarkable development of the functional food market. Incorporating herbal extracts as a rich source of bioactive compounds (BC) could be an effective solution to meet the high demand of consumers in terms of expanding the high-quality range of functional foods. The aim of this study is the valorization of the bioactive potential of *T. montanum* L., an understudied Mediterranean plant species, and the in-depth elucidation of a polyphenolic profile with a UHPLC-HR MS/MS and NMR analysis. The total phenolic content (TPC) and antioxidant capacity (AC) were determined on heat-assisted (HAE), microwave-assisted (MAE) and subcritical water (SWE) extracts. In terms of antioxidant capacity, SWE extracts showed the most notable potential (ABTS: 0.402–0.547 mmol eq Trolox g^−1^ dw, DPPH: 0.336–0.427 mmol eq Trolox g^−1^ dw). 12 phenolic compounds were identified in the samples of *T. montanum* from six microlocations in Croatia, including nine phenylethanoid glycosides (PGs) with total yields of 30.36–68.06 mg g^−1^ dw and 25.88–58.88 mg g^−1^ dw in HAE and MAE extracts, respectively. Echinacoside, teupolioside, stachysoside A, and poliumoside were the most abundant compounds HAE and MAE extracts, making *T. montanum* an emerging source of PGs.

## 1. Introduction

Nowadays, the valorization of traditional plant species plays an important role in reshaping global pharmaceutical and food markets, mainly due to the emergence of multidrug resistance, a growing awareness of the prevalence of chronic diseases, and a high consumer demand for herbal supplements. The undeniable importance of consuming herbal remedies for a balanced and healthy lifestyle has prompted global institutions to provide additional support for the sustainable development of traditional medicine [1]. A significant part of the drugs and nutraceuticals used in allopathic medicine (about 50%) are isolated products from natural sources and their derivatives [2]. Considering that most of the plant-derived bioactive compounds (BC) (74%) with proven medicinal uses are discovered within ethnomedicinal practices, many research studies aim to comprehensively characterize traditional herbal remedies [3].

Within *Lamiaceae*, *Teucrium* L. (Germander) is one of the least studied genera, and it has been used ethnopharmacologically in the Balkans for centuries, mostly in the form of infusions and decoctions. Germanders include more than 300 species and are mainly distributed in the Mediterranean region (96% of all species). On the Croatian coast, 12 *Teucrium* species have been identified so far, of which *T. montanum* is commonly used in the form of infusions for fevers and to strengthen the immune system. Among the local population, the phrase “brings the dead back to life” is often used to specifically refer to *T. montanum* to emphasize the importance of this herbal remedy [4]. Of the 72 ethnobotanical studies conducted so far on *Teucrium* L., 20 species have been confirmed for their medicinal purposes in various treatments, mainly for abdominal pain and gastrointestinal complaints reported in a total of 56 studies [5,6], followed by treating spasms, treating rheumatism and as blood purification therapy [7], treating hemorrhoids [8], treating respiratory diseases [9], strengthening the immune system [10], etc. The limited studies conducted so far report antimicrobial [11], anti-inflammatory [12], antiulcerogenic [13], radical scavenging [14], and lipid-protective activities [15] of *Teucrium* species. These effects are related to the presence of secondary metabolites, such as BC, *e.g.*, phenolic compounds, whose long-term consumption has been shown to play an important role in suppressing oxidative cell damage and the prevalence of various chronic diseases [16,17,18,19].

Among the polyphenols identified so far in *Teucrium* L., flavonoids represent the most extensive subgroup, *e.g.*, luteolin and its glycosides, apigenin and its glycosides, vicenin-2, isoquercetin, rutin, (epi)catechin, etc. [15,20,21]. Some studies also reported the presence of hydroxybenzoic and hydroxycinnamic acids, including gallic acid, protocatechuic acid, vanillic acid, caffeic acid, chlorogenic acid, *p*-coumaric acid, etc. [20,22,23]. The least studied phenolic subgroup is phenylethanoid glycosides (PGs), 16 of which have been identified in *Teucrium* species, with few studies reporting their content [21,22]. The recent research studies on PGs have reported promising antioxidant [24], neuroprotective [25], cardioprotective [26], hepatoprotective [27], antidiabetic [28], and antiviral properties [29].

To ensure the maximum recovery of phenolic compounds from plant material, the selection of an extraction technique and associated process parameters is a crucial step. In addition to the established conventional extraction techniques, advanced techniques have become the “state of the art” approaches for the sustainable production of polyphenol-rich herbal extracts [30,31], expanding their potential application in the food industry. Microwave-assisted extraction (MAE) is based on the heating of the solvent with microwave energy (electromagnetic frequencies between 300 MHz and 300 GHz) and is widely used for the successful recovery of relatively polar polyphenols, *e.g.*, phenolic acids [32]. In contrast, subcritical water extraction (SWE) uses an altered physical property of water—the dielectric constant—for the extraction of less polar BC. Under high pressure and temperature (100 and 374 °C), there is a reduction in viscosity and weakening of strong hydrogen bonds, making subcritical water more similar to less polar organic solvents [33].

In the context of the potential commercialization of traditional plant species as cost-effective and remarkable polyphenolic sources, an evaluation of bioactive potential and an in-depth elucidation of the polyphenolic profile of *T. montanum* from six different microsites in Dalmatia (Croatia) were performed. The prepared extracts HAE, MAE, and SWE were analyzed for their total phenolic content (TPC) and antioxidant capacity (AC). Elucidation of the unknown phenolic compounds was performed with ultrahigh performance liquid chromatography coupled with high-resolution mass spectrometry (UHPLC-HR MS/MS), together with isolation and nuclear magnetic resonance (NMR) for the most dominant polyphenols in all extracts studied. The chemometric analysis for the classification of the studied *T. montanum* samples was performed using the TPC, ABTS, and DPPH results as well as the HPLC results of the quantified PGs. This study will provide a comprehensive insight into the qualitative and quantitative polyphenolic composition of understudied *T. montanum*, which has emerged as a remarkable source of PGs. These results could be used for further studies focusing on the bioactive potential of *T. montanum* and possible incorporation into functional foods.

## 2. Materials and Methods

### 2.1. Plant Materials

All six analyzed plant samples of mountain germander (*T. montanum*) were collected in the Adriatic region of Croatia in five different municipalities, namely Seget, Kistanje, Stankovci, Hrvace, and Klis, with one sample collected in the mountain region of Svilaja. In this study, each sample (T) was marked with a number (1–6), depending on its geographical location.

T1 (voucher ID: 62598) was generously donated by OPG Piteša (Dalmatia Naturalis, Trogir, Croatia). Samples T2 (voucher ID: 75518) and T3 (voucher ID: 75520) were obtained from local suppliers Ljekovito bilje Jerkin j.d.o.o. (Zadar, Croatia) and MB Natural, d.o.o. (Bjelovar, Croatia), respectively. T4 (voucher specimen ID: 75519), T5 (voucher specimen ID: 62599), and T6 (voucher specimen ID: 75234) were collected by the experienced plant collectors. The voucher specimens T1–T5 and T6 were identified and deposited in the Herbarium Croaticum (Faculty of Natural Sciences, Zagreb, Croatia) and ZAGR Herbarium (Faculty of Agriculture, Zagreb, Croatia), respectively. Carefully sorted, dried, ground, and sieved areal plant parts (<450 μm) were used in all experiments.

### 2.2. Chemicals and Reagents

Folin–Ciocalteu’s reagent and sodium carbonate were purchased from Kemika (Zagreb, Croatia). Gallic acid (≥99%), echinacoside (≥98%), DPPH (2,2-Di(4-*tert*-octylphenyl)-1-picrylhydrazyl), apigenin (≥99%), ABTS (2,2′-Azino-bis(3-ethylbenzthiazoline-6-sulfonic acid)), and Trolox ((*S*)-6-Methoxy-2,5,7,8-tetramethylchromane-2-carboxylic acid) were supplied from Sigma Aldrich (St. Louis, MO, USA). Verbascoside (>90.3%) was supplied from HWI Pharma (Rülzheim, Germany). Acacetin (>99%) and diosmetin (≥95%) were purchased from Biosynth (Bratislava, Slovakia). Acetonitrile, formic acid, methanol, and ethanol were all of analytical grade (Fisher Chemicals, Hampton, NH, USA). Methanol-d_4_ (CD3OD, 99.8%) was purchased from Sigma-Aldrich, Inc. (St. Louis, MO, USA).

### 2.3. Methods

#### 2.3.1. Determination of Dry Weight

The content of dry matter (dw) of plant samples was measured according to the standard modified gravimetric method AOAC 930.15 (1990) [34]. The dry matter for all six samples was determined to be in the range of 90.22–91.30%.

#### 2.3.2. Preparation of Water Extracts via HAE, MAE, and SWE Technique

In this study, the optimal extraction conditions, *i.e.*, solid-to-solvent ratio, temperature, and time, were selected according to the previously conducted experimental design using response surface methodology [35].

For HAE, the plant sample (1 g) was weighed, mixed with boiled distilled water (100 mL), and extracted for 30 min at 100 °C in a water bath (Inkolab d.o.o., Zagreb, Croatia). The MAE performance (0.5 g:50 mL, 90 °C, and 9 min) was performed using a microwave extraction system (Ethos Easy, Milestone, Sorisole, Italy) in sealed plastic vials positioned on a rotating diffuser under 900 W with precise temperature control. SWE (1 g:100 mL, 200 °C, and 15 min) was performed in a batch system (4740 Stainless Steel series, Parr Instruments, Moline, IL, USA) with a maximum operating temperature of 540 °C and a pressure of 580 bar. Extraction was performed in a vessel (75 mL) with constant stirring (600 rpm), and pressure was controlled with N_2_ and temperature (TRC oven).

After completion of all extraction techniques, the solid–solvent mixture was cooled and filtered from the solid residues, with additional washing of the solids to maximize the extracted soluble fraction. The water extracts obtained after extraction were immediately analyzed for TPC, AC, and HPLC-UV-DAD. In addition, all extracts were concentrated on a rotary evaporator (RV 8, IKA, Staufen, Germany), freeze-dried (Alpha 1– LSC, Christ, Germany), and stored in a freezer (−18 °C) for NMR and LC–HR MS/MS analysis.

#### 2.3.3. Total Phenolic Content (TPC)

TPC was analyzed in the prepared water extracts according to assay by Singleton and Rossi (1965) [36]. An amount of 7.9 mL of water was mixed with the sample (100 μL) and the diluted Folin–Ciocalteau reagent at a ratio of 1:1, *v*/*v* (500 μL). After alkalizing the mixture with 20% sodium carbonate, *w*/*v* (1.5 mL), the samples were incubated in the dark (25 °C) for 2 h. The absorbance of the blue colored reaction mixture was measured spectrophotometrically at 765 nm. The blank sample contained water instead of the extract. The result of subtracting the absorbance of the reaction mixture from the absorbance of the blank sample (ΔA) was used in the equation of standard calibration curve (10–100 μg mL^−1^, R^2^ = 0.99) for gallic acid to calculate TPC. Results were expressed as gallic acid equivalents per gram of dry weight of sample (mg eq GA g^−1^ dw).

#### 2.3.4. Antioxidant Capacity (AC)

The ABTS radical scavenging test was performed by Re et al. (1999) [37]. First, an ABTS radical stock solution was prepared by dissolving the ABTS salt in water to reach a final concentration of 7 mM and then oxidizing with (140 mM) potassium persulfate to reach a final concentration of 2.45 mM potassium persulfate. An amount of 2 mL of the 1% diluted ABTS stock radical cation solution with 96% ethanol (1:99, *v*/*v*) and 20 μL of a diluted extract were mixed vigorously and allowed to stand in the dark (25 °C). The absorbance of the reaction mixture was measured at 734 nm. The blank control contained the ABTS reagent and 96% ethanol (*v/v*) in place of the extract. The variable ΔA was used in the Trolox standard calibration curve equation (10–90 μg mL^−1^, R^2^ = 0.99) to calculate the total ABTS radicals that reacted with the antioxidants present.

The assay for DPPH free radical scavenging was performed by Brand-Williams et al. (1995) [38]. The reaction mixture of the prepared 0.094 mM DPPH reagent in methanol (3.9 mL) and the extract (100 μL) was shaken and left in the dark (25 °C). The reduction in purple color was measured spectrophotometrically at 515 nm after 30 min. The blank sample contained all reagents and methanol instead of the extract. To calculate the antioxidant capacity measured with the DPPH reagent, ΔA was substituted into the equation of the Trolox standard calibration curve (10–90 μg mL^−1^, R^2^ = 0.99).

The results of both the ABTS and DPPH assays were expressed as mmol Trolox equivalent per gram of dry weight of sample (mmol eq Trolox g^−1^ dw).

#### 2.3.5. Identification and Structure Characterization Using NMR Spectroscopy

##### Isolation of Dominant Phenylethanoid Glycosides

**Figure 1:**
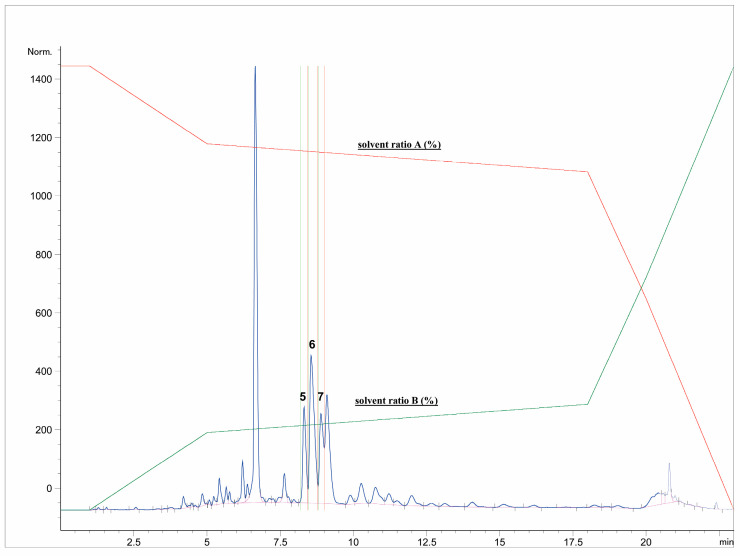
Isolation of dominant PGs: PH5,, PH6 and PH7 from T2_HAE sample using preparative HPLC-UV-DAD (recorded at 320 nm). The red and green lines represent the gradient of solvent A and solvent B, respectively, during chromatographic analysis.

First, the freeze-dried extract (0.53 g) was dissolved in purified water (10 mL) and precipitated with 96% ethanol (1:1). The supernatant was centrifuged (9500 rpm, 10 min), separated, and filtered through RC-HPLC filters (0.20 μm). Separation of targeted polyphenolic compounds was performed using the 1260 Infinity II Manual Preparative LC System (Agilent Technologies, Santa Clara, CA, USA). Chromatographic separation was performed on a Zorbax Eclipse XDB-C18 column (L × I.D. 250 mm × 9.4 mm, 5 μm particle size) (Agilent Technologies, Santa Clara, CA, USA) with gradient elution using mobile phase A (1% formic acid in water, *v*/*v*) and mobile phase B (1% formic acid in acetonitrile, *v*/*v*). The optimized method parameters were as follows: 0–1 min: 7% B phase, 1–5 min: 7–15% B phase, 5–18 min: 15–25% B phase, 18–20 min: 25–40% B phase, 20–23 min: 40–70% B phase, followed by a 5-min equilibration to the original analysis conditions. The column temperature was maintained at 25 °C, the flow rate was 9 mL min^−1^, and the injection volume was set to 400 μL. UV signal detection was monitored at 320 nm using a diode array detector (DAD). The selected trigger mode, *i.e.*, timetable (combination of time intervals and peaks), was defined primarily according to the specific retention time for each target compound, *i.e.*, the base peaks. Each isolated phenolic fraction was collected in a separate glass vial during each sequence run, combined in a beaker according to the specific retention time, and then concentrated into dry residue in the R-215 rotary evaporator (Büchi Labortechnik AG, Flawil, Switzerland). Figure 1 represents HPLC-UV-DAD chromatogram of the separated and isolated PGs from the T2_HAE sample, labeled as follows: PH5 (Rt = 8.31 min), PH6 (Rt = 8.55 min), PH7 (Rt = 9.09 min).

##### NMR Spectroscopy

The most dominant components in all *T. montanum* extracts (Figure 1), phenolic compounds PH5, PH6, and PH7 were isolated and further analyzed with NMR spectroscopy. PH5 (22.8 mg), PH6 (26.2 mg), and PH7 (26.1 mg) were dissolved in 600 μL of metanol-d_4_ (CD_3_OD) and transferred to 5 mm NMR tubes.

One-dimensional (^1^H, ^13^C-DEPTq) and two-dimensional (COSY, ^1^H-^13^C HSQC, ^1^H-^13^C HMBC, NOESY) NMR spectra were recorded using standard pulse sequences on Avance Neo 600 MHz spectrometer (Bruker, Billerica, MA, USA) equipped with 5 mm Prodigy Cryoprobe (CPP1.1 TCl 600S3 H&F-C/N-D-05 Z XT) and single axis Z-gradient. NOESY spectra were obtained with a mixing time of 400 ms. Residual methanol-d_4_ solvent signal (3.31 ppm for proton and 49.15 ppm for carbon) was used for referencing. Experimental parameters for all recorded spectra can be found in Supplementary Material (Figures S1–S29). For the comparison and unambiguous structure characterization of compounds, analytical standards with similar structures, *i.e.*, echinacoside and verbascoside, were analyzed under same experimental conditions. These phenolic compounds were identified in all *T. montanum* samples.

#### 2.3.6. Identification Using HPLC-UV-DAD and UHPLC-HR MS/MS Techniques

##### Isolation of Polyphenolic Compounds Using Analytical HPLC-UV-DAD

First, the selected polyphenolic fractions were isolated from the extract according to the retention time. For this purpose, an HPLC-UV-DAD Agilent 1200 Series (Agilent, Santa Clara, CA, USA) coupled to an Agilent 1260 Infinity II (Agilent, Santa Clara, CA, USA) fraction collector was used. Components were analyzed on a Zorbax extend C-18 analytical column (L× I.D. 250 mm × 4.5 mm, 5 μm particle size) (Agilent Technologies, Santa Clara, CA, USA) using a gradient elution with aqueous mobile phase A (1% formic acid in water, *v/v*) and organic mobile phase B (1% formic acid in acetonitrile, *v/v*), as follows: 0–5 min: 3% B, 5–45 min: 40% B, 45–47 min: 70% B, 47–52 min: 70% B. The flow rate of the mobile phases was 1 mL min^−1^ at a constant temperature of 25 °C. The duration of the method was 52 min with an additional 10 min equilibration to the initial conditions of the analysis. The volume of the injected sample was 20 μL. Each fraction was isolated and collected in glass vials with the trigger mode running on a predetermined timetable. The mobile phase was removed from the collected polyphenolic fractions in a rotary evaporator, and the dry residue of each fraction was resuspended in 80% acetonitrile (200 μL). UV spectra were recorded in the range of 260–370 nm using DAD.

The extract T2_HAE was selected for isolation of the unidentified phenolic compounds. All isolated phenolic compounds were present in all *T. montanum* samples, regardless of the extraction technique used.

##### UHPLC-HR MS/MS and HPLC-UV-DAD Analysis

Polyphenolic compounds were analyzed with ultrahigh-pressure liquid chromatography and high-resolution mass spectrometry using an Agilent 6550 Series Accurate–Mass Quadrupole Time-of-Flight (Q-TOF) mass spectrometer (Agilent Technologies, Santa Clara, CA, USA) coupled with Agilent 1290 Infinity II LC (Agilent Technologies, Santa Clara, CA, USA). Chromatographic separation was performed on a Zorbax SB -C18 column (L × I.D. 100 mm × 2.1 mm, 1.8 μm particle size). The mobile phase consisted of 0.1% formic acid in water (A) and in acetonitrile (B) with gradient elution. The gradient of the mobile phase was as follows.

The values were 0–15 min: 5–95% B phase and 15–17 min: 95% B phase. The temperature of the column was maintained at 40 °C, the flow rate of the mobile phase was 0.2 mL min^−1^, and 2 μL of the sample were injected. Electrospray ionization was performed in negative mode in the mass range 100–1200 *m/z* for MS analysis and 50–850 *m*/*z* for MS/MS analysis. The analysis parameters were set as follows: Capillary potential, 3500 V; nozzle voltage, 1000 V; nebulizer pressure, 35 psi; gas sheath flow rate 11 L min^−1^; sheath gas temperature, 350 °C; drying gas flow rate, 14 L min^−1^; drying gas temperature, 200 °C. Nitrogen was used as the sheath gas and drying gas. The collision energies applied for fragmentation of selected precursor ions were 10, 20, and 40 V. Identification of the analyzed polyphenolic components was performed by comparing the obtained data for precursor ions and fragment ions with available data from the literature and available databases for mass spectra identification (Sci Finder, Columbus, OH, USA).

HPLC-UV-DAD identification was performed according to the exact method described in 2.3.6., only with a change in the injection volume of sample (5 μL). Echinacoside and verbascoside in all samples were identified compared to the retention times of the secondary reference standards.

#### 2.3.7. HPLC-UV-DAD Quantification of Identified Polyphenolic Compounds

Echinacoside and verbascoside were quantified based on the specific retention time of their analytical reference standards (the range of the standard curve for echinacoside and verbascoside was 3–90 μg mL^−1^ and 99–3 μg mL^−1^, respectively). The compounds identified with both NMR spectra and HRMS data (PH5, PH6, and PH7) were quantified based on their high structural similarity to the echinacoside standard curve, having the same phenolic constituents, *i.e.*, caffeic acid and hydroxytyrosol, and a similar molar mass and type of esterified monosaccharides. The results were expressed in mg of echinacoside equivalents per gram of dry matter (mg eq ECH g^−1^ dw). Other phenylethanoid compounds identified only with experimental HR MS data were also quantified using the echinacoside (R^2^ = 0.99) or verbascoside (R^2^ = 1) standard curve, expressed as mg eq ECH g^−1^ dw or mg eq VERB g^−1^ dw for echinacoside and verbascoside, respectively. The identified flavonoids diosmin (diosmetin-7-*O*-rutinoside), acacetin-7-*O*-rutinoside, and vicenin-2 (apigenin-6,8-di-C-glucoside) were quantified using the calibration curve for secondary reference standards, i.e., diosmetin (R^2^ = 0.99, 3.9–97.5 μg mL^−1^) (mg DS g^−1^ dw), acacetin (R^2^ = 0.99, 3.6–90 μg mL^−1^) (mg AC g^−1^ dw), and apigenin (R^2^ = 0.99, 2.4–120 μg mL^−1^) (mg AP g^−1^ dw).

#### 2.3.8. Statistical Analysis

Results of TPC, antioxidant capacity evaluated with ABTS and DPPH, and HPLC-UV-DAD quantification of individual phenolic compounds were analyzed using one-way analysis of variance with Tukey’s post hoc test (*p* < 0.05) in the OriginPro 2023b (10.05; trial version) software (Origin Lab Corporation, Northampton, MA, USA).

Pearson’s correlation coefficients were determined in Microsoft Excel 2016 (Microsoft Corporation, Redmond, WA, USA) to evaluate the linear correlation between TPC and antioxidant capacity (DPPH and ABTS).

Multivariate method PCA was performed to reduce the data sets, *i.e.*, experimentally measured dependent variables, by transforming them into new orthogonal variables—principal components—each representing a linear combination of the variables originally used. The TPC, ABTS, and DPPH results as well as the quantified PGs, the most abundant polyphenolic group in all samples of *T. montanum*, were used as variables for PCA performance in OriginPro 2023b (10.05; trial version) software (Origin Lab Corporation, Northampton, MA, USA).

## 3. Results and Discussion

### 3.1. Determination of TPC and AC

To facilitate the mass transfer of BC using water as a “clean” solvent for maximum extraction recovery from *T. montanum* plant material, optimized parameters for conventional and innovative extraction techniques, *i.e.*, MAE and SWE, were previously applied. The results of the TPC assay, which is known as an established spectrophotometric method for the rapid screening of phenolic content, are shown in Table 1.

As can be seen, similar recovery of TPC content was obtained for all samples for the extracts HAE (46.16–71.60 mg eq GA g^−1^) and MAE (46.52–71.80 mg eq GA g^−1^). The only research studies available for comparison with *Teucrium* species are those using a traditional preparation, *i.e.*, a conventional extraction method without heat transfer, with organic solvents or a mixture of organic and aqueous solvents. The reported TPC values for the 100% water macerate of *T. montanum* (136.97 mg eq GA g^−1^ dw) and the 50% ethanolic extract of *T. polium* (60.80 mg eq GA g^−1^ dw) were generally lower than those obtained in this study, considering that the authors reported the results per g dry weight of the extract [14,15]. This may be attributed to the higher extraction temperature, which has been shown to maximize polyphenol yield, as it promotes mass diffusion as a result of the higher kinetic energy of the molecules in the solvent–sample system. At MAE, the main advantage is the reduction of the extraction time due to a rapid energy transfer from ionic conduction and dipole rotation in the electromagnetic field [39].

However, all SWE extracts in this study were significantly different (*p* < 0.05) from samples HAE and MAE (84.50–109.55 mg eq GA g^−1^), suggesting a higher bioactive potential. This is in good agreement with the results of Nastić et al. (2018) [40], where a phenol-rich *T. montanum* extract (143.89–174.61 mg eq GA g^−1^ dw of extract) with flavonoid glycosides as the most dominant components was obtained (temperature range: 60–200 °C, 1 g:10 mL, extraction time −30 min), suggesting that SWE is an effective technique for relatively non-polar polyphenolic compounds. However, despite the improved properties of subcritical water related to lower viscosity and high diffusivity, the application of temperatures above the boiling point of water could have the opposite effect due to the hydrothermal degradation of BC [41].

Considering the important role of antioxidants in neutralizing free radicals and peroxides through electron/proton donation, the antioxidant capacity was also determined using standard assays, ABTS and DPPH.

Table 1 shows that the results of AC for all extract types follow the TPC trend. HAE (ABTS: 0.221–0.547 mmol eq Trolox g^−1^ dw, DPPH: 0.209–0.338 mmol eq Trolox g^−1^ dw) and MAE (ABTS: 0.206–0.329 mmol eq Trolox g^−1^ dw, DPPH: 0.171–0.331 mmol eq Trolox g^−1^ dw) extracts gave similar antioxidant capacity. However, the SWE extracts had about twice the antioxidant capacity (*p* < 0.05) of the conventionally prepared extracts for all six *T. montanum* samples (ABTS: 0.402–0.547 mmol eq Trolox g^−1^ dw, DPPH: 0.336–0.427 mmol eq Trolox g^−1^ dw). The results of Pearson correlation coefficients greater than 0.80 for TPC, ABTS, and DPPH between all extraction techniques indicate very strong positive correlations between the studied variables (Table 2).

Compared to other studies on the free radical scavenging properties of *Teucrium* species [15,42], the higher values we obtained here for the antioxidant capacity of *T. montanum* water decoction are probably the result of many different factors, *e.g.*, extraction parameters, species, microsites, specific growth conditions, etc. Nastić et al. (2018b) [40] reported higher DPPH values for SWE extract in the range of 60–160 °C while significantly lower antioxidant capacity was observed at 180 °C, which may be due to the degradation of antioxidants via pyrolysis. Since there is not always a positive correlation between the antioxidant capacity results and the individually recovered phenolic compounds when SWE is applied [43,44], the hydrolysis of phenolic compounds under the influence of specific SWE conditions could lead to the formation of Maillard products, whose radical scavenging properties are preserved [45].

### 3.2. NMR Structure Characterization of Selected PGs

PGs are structurally characterized by phenylethyl moiety, *e.g.*, hydroxytirosol, which forms a glycosidic bond with disaccharide or oligosaccharide units, and phenylpropanoid moiety, *e.g.*, phenolic acid, which forms an ester bond with the glycone part. Due to the presence of isomeric compounds (same molecular weight) and sugars with strong similarities and subtle structural differences, the elucidation of PGs structures with NMR spectroscopy in combination with high-resolution mass spectrometry (HR MS) is highly recommended.

To this end, the structures of the three phenylethanoid glycosides PH5, PH6, and PH7, which were found to be dominant phenolic compounds in all extracts, were elucidated with NMR spectroscopy. In addition, echinacoside and verbascoside were given as standard compounds to facilitate the structural elucidation of compounds with an unknown structure in fractions PH5, PH6, and PH7 by comparing the chemical shifts of protons and carbon chemical shifts and the coupling constants. Both compounds were identified and quantified in all HAE, MAE, and SWE extracts by comparing the retention times of the analytical standards. A complete list of chemical shifts and their comparison among all compounds studied can be found in Table S1 for the proton shifts and Table S2 for the carbon shifts. The values of the proton–proton coupling constants ^n^J_H,H_ (Hz) are represented in Table S3.

**Figure 2:**
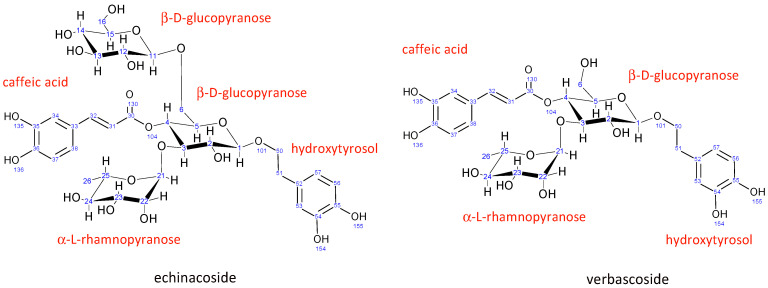
Structure and numbering for echinacoside and verbascoside.

Echinacoside is a caffeic acid glycoside first isolated from *Echinacea angustifolia* in 1950. Its structure (Figure 2) was elucidated using NMR spectroscopy in 1982 to be β-(3′,4′-dihydroxyphenyl)-ethyl-*O*-α-L-rhamnopyranosyl-(1→3)-*O*-β-D-[β-D-glucopyranosyl-(1→6)]-(4-*O*-caffeoyl)-glucopyranoside [46].

The structure of verbascoside was determined in the same year (1982) by Andary et al. [47]. It is structurally very similar to echinacoside, the main difference being the absence of a second β-D-glucopyranose sugar attached to the central glucopyranose sugar unit. All other units in verbascoside have nearly identical chemical shifts (Tables S1 and S2) and coupling constants (Table S3), suggesting the same conformation as echinacoside.

**Figure 3:**
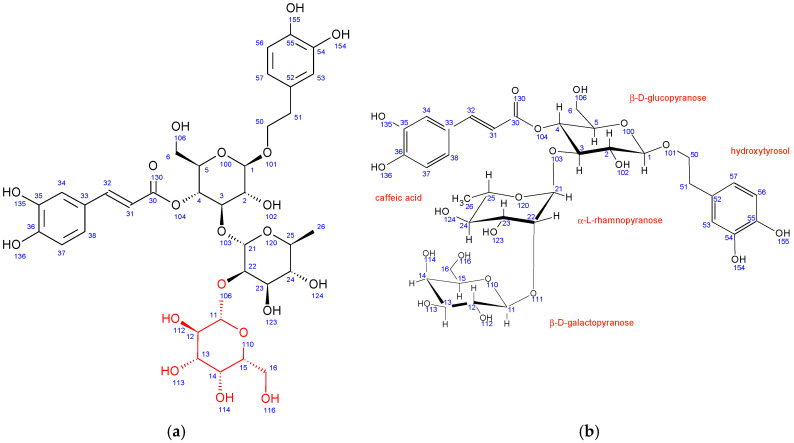
Fraction PH5 (teupolioside): (**a**) structure and numbering, (**b**) conformation of sugar units.

In comparison to echinacoside, the major structural difference of fraction PH5 is the absence of β-D-glucopyranose at position 6 combined with the presence of a different sugar unit attached to position 22 of α-L-rhamnopyranose sugar, as evidenced by strong ^1^H-^13^C HMBC interaction between C11 and H22. Chemical shifts, coupling constants, 2D NMR interactions, and NOE cross peaks revealed the structure of this sugar to be β-D-galactopyranose. It adopts the same chair conformation as β-D-glucopyranose (Figure 3). 

A structure search using SciFinder (Columbus, OH, USA) revealed that the PH5 compound is already described in the literature and is known as teupolioside [48]. The original NMR data could not be compared to the data from this study due to the different solvent used (pyridine/water).

**Figure 4:**
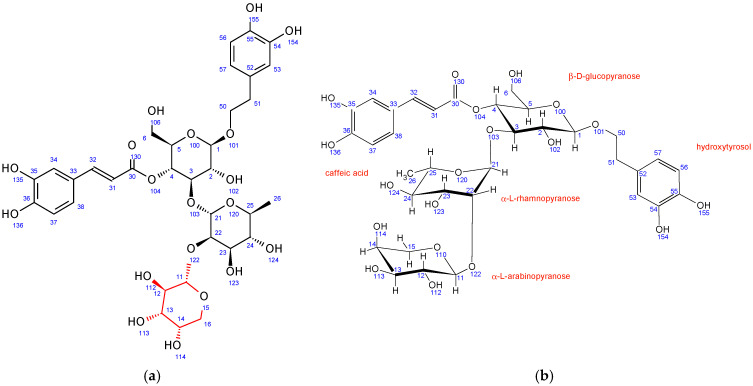
Fraction PH6 (stachysoside A): (**a**) structure and numbering, (**b**) conformation of sugar units.

Similar to PH5, PH6 also does not have β-D-glucopyranose at position 6 while having a different sugar unit attached to the position 22 of α-L-rhamnopyranose sugar. Its ^1^H-^13^C HMBC NMR spectrum also shows strong interactions between C11 and H22. However, in PH6, the chemical shifts, coupling constants, 2D NMR interactions, and NOE cross peaks revealed the structure of this sugar to be α-L-arabinopyranose (Figure 4). It adopts the thermodynamically stable 1C_4_ ring conformation with 14OH substituent in axial position. A structure search using SciFinder (Columbus, OH, USA) revealed that the PH6 compound is also described in the literature and is known as stachysoside A [49]. The original NMR data were compared to ours and were a good match.

**Figure 5:**
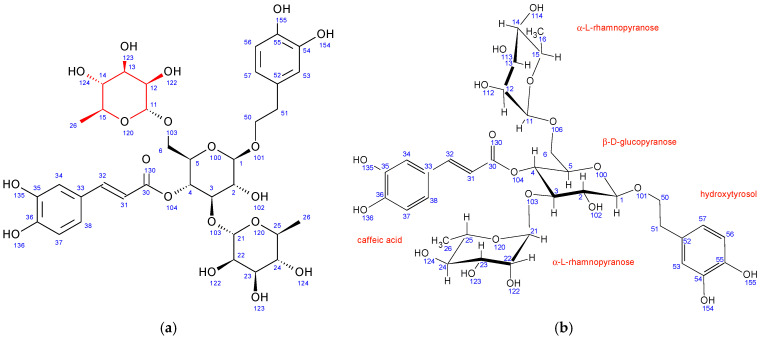
Fraction PH7 (poliumoside): (**a**) structure and numbering, (**b**) conformation of sugar units.

In comparison to echinacoside, PH7 has a different sugar unit attached to position 6 of the central β-D-glucopyranose, as evidenced by a strong ^1^H-^13^C HMBC interaction between C11 and H6. The chemical shifts, coupling constants, 2D NMR interactions, and NOE cross peaks revealed its structure to be α-L-rhamnopyranose, identical to the unit attached to position 3 of the same central sugar (Figure 5). Both rhamnopyranoses adopt the same ^1^C_4_ ring conformation.

A structure search using SciFinder (Columbus, OH, USA) revealed that the PH7 compound is also described in the literature and is known as poliumoside [50] The original NMR data could not be compared to ours due to the different solvent used (DMSO/TFA).

### 3.3. HPLC-UV-DAD and UHPLC-HR MS/MS Identification

Due to the high accuracy and sensitivity in structure elucidation, the identification of the unknown polyphenols was performed with UHPLC-HR MS/MS using the LC-q-TOF mass spectrometer.

**Figure 6:**
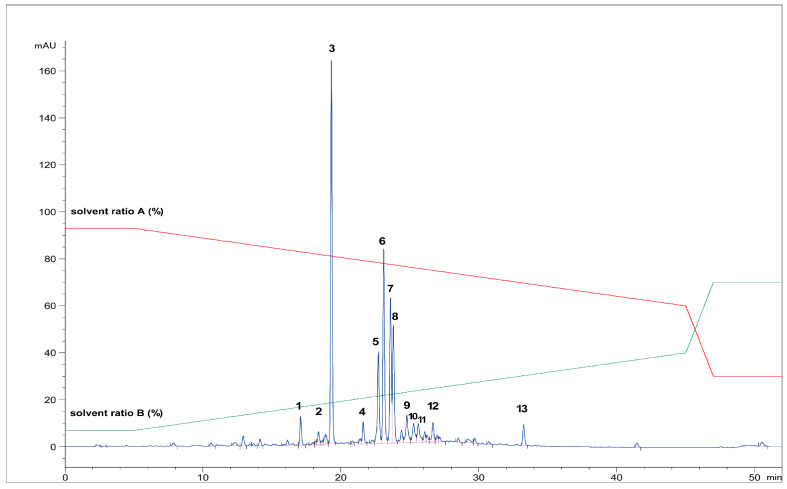
The HPLC chromatogram of all analyzed phenolic fractions from the T2_HAE extract (1–13) (recorded at 320 nm).The red and green lines represent the gradient of solvent A and solvent B, respectively, during chromatographic analysis.

Figure 6 shows the HPLC chromatogram of all analyzed phenolic compounds in *Teucrium m*. The phenolic fractions identified with the UHPLC-HR MS/MS technique were the following: PH1, PH2, PH4, PH5, PH6, PH7, PH9, PH10, PH12, and PH13 while PH3 (Rt = 19.20) and PH8 (Rt = 23.62) were identified using the HPLC-UV-DAD technique by comparing the retention time of secondary reference standards, *i.e.*, echinacoside (PH3) and verbascoside (PH8). All identified compounds were already described in the literature. PH11 was not identified.

#### 3.3.1. UHPLC-HR MS/MS Identification of PGs

The results of the UHPLC-HR MS/MS analyses of unknown polyphenolic fractions of *T. montanum* species are presented in Table 3. 

Among the identified compounds were seven PGs: β-OH-forsythoside (PH2) [51], jionoside A (PH4) [52], teupolioside (PH5) [53], stachysoside A (PH6) [54], poliumoside (PH7) [55], forshythoside B (PH9) [52], and isoverbascoside (PH10) [51], and three were flavone glycosides: vicenin-2 (PH1) [56], diosmin (PH12) [53], and acacetin-7-*O*-rutinoside (PH13) [57].

**Table 3:** UHPLC-HRMS/MS experimental data of *T. montanum* extract.

Fraction	t_R_ of Fraction (min)	Identified Compound	Molecular Formula	Error (ppm)	Calculated Mass (*m*/*z*)	Observed Mass [M-H]^−^ (*m*/*z*)	MS/MS (*m*/*z*) and Abundance of Each Fragment Ion (%)	Reference
PH1	16.93	vicenin-2	C_27_H_30_O_15_	3.37	593.1512	593.1532	MS^2^ [593.1532]: 353.0669 (100), 383.0773 (60.8), 473.1095 (18.5), 325.0716 (10.9), 413.0876 (6.6), 503.1192 (6.4)	[56]
PH2	18.25	β-OH-forsythoside B	C_34_H_44_O_20_	3.37	771.2353	771.2379	MS^2^ [771.2379]: 179.0345 (100), 753.2234 (24.7), 661.1967 (5.3), 591.1925 (13.5)	[51]
PH4	21.47	jionoside A	C_36_H_48_O_20_	3.88	799.2666	799.2697	MS^2^ [799.2697]: 623.2192 (100), 175.0399 (60.9), 477.1604 (5.8),	[52]
PH5	22.55	teupolioside	C_35_H_46_O_20_	1.53	785.2510	785.2522	MS^2^ [785.2522]: 623.2189 (52.3), 161.0241 (100), 477.1609 (2.61),	[53]
PH6	22.93	stachysoside A	C_34_H_44_O_19_	1.46	755.2404	755.2415	MS^2^ [755.2415]: 161.0244 (100), 593.2097 (42.2), 461.1660 (14.08), 623.1972 (2.5), 315.1078 (2.6)	[54]
PH7	23.43	poliumoside	C_35_H_46_O_19_	2.60	769.2561	769.2581	MS^2^ [769.2581]: 161.0244 (100), 607.2240 (35.1), 461.1649 (3.4),	[55]
PH9	24.59	forsythoside B	C_34_H_44_O_19_	0.66	755.2404	755.2409	MS^2^ [755.2409]: 161.0241 (100), 593.2080 (55.1), 461.1656 (16.4), 623.1980 (6.6), 315.1079 (3.1)	[52]
PH10	25.07	isoverbascoside	C_29_H_36_O_15_	2.84	623.1981	623.1999	MS^2^ [623.1999]: 161.0249 (100), 113.0242 (16.4), 461.1659 (15.4), 315.1076 (3.4), 251.0551 (1.5)	[51]
PH11	25.42	unknown compound	/	/	/	635.1993	MS^2^ [635.1993]: 455.1345 (100), 309.0977 (97.5), 163.0396 (29.5), 187.0395 (29.5)	/
PH12	26.48	diosmin	C_28_H_32_O_15_	0.00	607.1668	607.1668	MS^2^ [607.1668]: 299.0558 (100), 284.0322 (59.8)	[53]
PH13	33.02	acacetin-7-*O*-rutinoside	C_28_H_32_O_14_	3.04	591.1719	591.1737	MS^2^ [591.1737]: 283.0620 (100), 268.0377 (39.0)	[57]

Analytical reference standards of echinacoside and verbascoside, whose characteristic fragmentation patterns are shown in Table 4, were used for the optimization of the MS/MS analysis in negative mode.

As shown in Table 3 and Table 4, similar fragmentation patterns of PGs with caffeic acid as part of aglycone were observed with the general formation of three characteristic moieties: [M-H-caffeoyl unit]^−^, [caffeoyl unit/ion]^−^ after the cleavage of a caffeoyl unit/ion, and [M-H-glycosyl unit]^–^ after a neutral loss of different glycosyl fragments. Thus, most of the generated fragment ions were as follows: [M-H-161]^−^, [M-H-179]^−^, [161]^−^, [179]^−^ for the caffeoyl unit/ion, [M-H-162]^−^ for the glucosyl and galactosyl unit (162 Da), [M-H-146]^−^ for the rhamnosyl unit (146 Da), and [M-H-132]^−^ for the apiosyl and arabinosyl unit (132 Da).

Analytical PGs standards, echinacoside and verbascoside, were first subjected to an HR MS/MS analysis to serve as an example of the fragmentation pattern of compounds with similar structure. For echinacoside (C_35_H_46_O_20_), fragment ions corresponding to the *m*/*z* 623.2200 (loss of caffeoyl moiety), *m*/*z* 477.1603 (loss of glucosyl and rhamnosyl units), and *m*/*z* 161.0245 (cleavage of caffeoyl moiety) were observed. The parent ion [M-H]^−^ of verbascoside (C_29_H_36_O_15_) at *m*/*z* 623.1996 exhibited the following fragmentation pattern; *m*/*z* 461.1665 (loss of caffeoyl unit), *m*/*z* 315.1081 (loss of caffeoyl unit and rhamnose), and *m*/*z* 161.0248 (caffeoyl unit-H_2_O).

Compound PH4 was identified as jionoside A, with a parent ion at *m*/*z* 799.2697 (C_36_H_47_O_20_). It produced a base peak at *m*/*z* 623.2192, indicating a loss of the methylated caffeoyl unit (176 Da). The formation of the fragment ion at *m*/*z* 477.1605 is likely due to the loss of the glucosyl (162 Da) and demethylated caffeoyl (161 Da) moieties.

Compounds PH5, PH6, and PH7 were characterized as teupolioside (*m*/*z* 785.2510, C_35_H_46_O_20_), stachysoside A (*m*/*z* 755.2415, C_34_H_44_O_19_), and poliumoside (*m*/*z* 769.2581, C_35_H_46_O_19_), respectively, with the same base peak at *m*/*z* 161, indicating the presence of the caffeoyl unit. The fragments that has an *m*/*z* 477.1609 for teupolioside, *m*/*z* 461.1660 for stachysoside A, and *m*/*z* 461.1649 for poliumoside, respectively, refer to the loss of glucosyl and rhamnosyl units [M-H-162-146]^−^, the loss of glucosyl and arabinosyl units [M-H-162-132]^−^, and the loss of glucosyl and rhamnosyl units [M-H-162-146]^-^. In addition, the fragment ion *m*/*z* 315.1078 confirms the presence of glucose, rhamnose, and arabinose [M-H-162-146-132]^−^ in PH5 (stachysoside A).

PH9 was identified as forsythoside B (C_34_H_43_O_19_) due to an [M-H]^−^ ion at *m*/*z* 755.2409 and generated fragment ions at *m*/*z* 593.2080 [M-H-caffeoyl unit]^−^, *m*/*z* 623.1980 [M-H-apiosyl unit]^−^, *m*/*z* 461.1656 [M-H-caffeoyl-apiosyl unit]^−^, *m*/*z* 161.0241 [M-H-caffeoyl-apiosyl-glucosyl-hydroxyphenylethyl]^−^, and *m*/*z* 315.1079 [M-H-caffeoyl-apiosyl-rhamnosyl]^−^.

The fragmentation pattern assigned to the generated product ions at *m*/*z* 753.2247 [M-H-H_2_O]^−^, *m*/*z* 661.1967 [M-H-C_6_H_6_O_2_]^−^, and *m*/*z* 591.1925 [M-H-H_2_O-caffeoyl unit]^−^ and corresponding to the loss of the caffeic acid ion at *m*/*z* 179.0345 identified PH2 as a β-OH-forsythoside (C_34_H_44_O_20_) with the parent ion [M-H]^−^ at *m*/*z* 771.2379.

PH10 (C_29_H_35_O_15_) with the parent ion at *m*/*z* 623.1999 showed the same product ions as verbascoside but with a different retention time, indicating the presence of its isomer, *i.e.*, isoverbascoside.

#### 3.3.2. UHPLC-HR MS/MS Identification of Flavonoid (di)glycosides

The presence of three flavonoids in *T. montanum* samples was preliminarily confirmed with HR MS/MS experiments, including one flavone C-glycoside, *i.e.*, vicenin-2, and two flavonoid *O*-rhamnoglucosides, *i.e.*, diosmin and acacetin-7-*O*-rutinoside. All three identified compounds were already found in the studied plant species [21,58].

Vicenin-2 (PH1), formerly known as apigenin-6,8-di-C-β-D-glucoside (C_27_H_30_O_15_), was identified based on the parent ion generated at *m*/*z* 593.1532 and the fragmentation pattern characteristic of C-diglucosylflavones as follows: *m*/*z* 503.1192, *m*/*z* 473.1095, *m*/*z* 383.0773, *m*/*z* 353.0669, *m*/*z* 325.0716, and *m*/*z* 191.0346 [59,60]. The fragment ion at *m*/*z* 473.1095 is the result of the cross cleavage of the hexose residue and water molecules in C-glycosil flavones [M-H-120]^−^. The presence of apigenin (Apg) is confirmed by the formation of characteristic fragments at *m*/*z* 383.0773 [Apg+113]^−^ and *m*/*z* 353.0669 [Apg+83]^−^ [61,62]. This flavone C-glycoside was not detected in any of the *T. montanum* SWE extracts.

PH12 and PH13, which were eluted as the most nonpolar compared with the previously studied compounds, were characterized as diosmin (C_28_H_32_O_15_, diosmetin-7-*O*-rutinoside) and acacetin-7-*O*-rutinoside (C_28_H_32_O_14_) in HAE, MAE, and SWE extracts, respectively. The cleavage of glycosidic acid bonds from the parent ion of diosmin (*m*/*z* 607.1668) and acacetin-7-*O*-rutinoside (*m*/*z* 591.1737) led to the formation of fragment ions *m*/*z* 299.0558 and *m*/*z* 283.0620, respectively. In addition, the loss of methyl groups (15 Da) led to the formation of smaller fragment ions at *m*/*z* 284.0322 and 268.0377, respectively.

### 3.4. HPLC-UV-DAD Quantification of Identified Polyphenols

The quantification of the identified phenylethanoids and flavonoids with UHPLC-HR MS/MS (Q-TOF), NMR spectroscopy and HPLC-UV-DAD was performed via HPLC-UV-DAD. The results are shown in Table 5.

Echinacoside, teupolioside, stachysosyde A, poliumoside, and verbascoside were the most represented phenolic compounds in all HAE, MAE, and SWE extracts of *T. montanum*. Heat-assisted extraction proved to be the most suitable for the extraction of phenylethanoid glycosides from all six samples, with total PGs yields ranging from 30.36 to 68.06 mg g^−1^ dw. Although MAE seemed to be less effective in PGs extraction (*p* < 0.05) (25.88–58.88 mg g^−1^ dw), this technique has the advantage of a short extraction time (9 min) compared to HAE (30 min). On the other hand, SWE extracts had the lowest PGs content (4.62 to 21.32 mg g^−1^ dw) (*p* < 0.05). These results could be explained by the lower dielectric constant of the solvent, which favors the mass transfer of polyphenols with moderate and/or low polarity. In addition, due to the high temperature (200 °C), the hydrolysis of glycoside bonds probably occurred, resulting in the significant degradation of PGs. In comparison, Nastić et al., 2018b [40], used 160 °C and reported only the presence of phenolic acids, *i.e.*, gallic acid, protocatehuic acid, chlorogenic acid, and caffeic acid.

Considering the available data for the PGs profile of *T. montanum*, Mitreski et al. (2014) [21] reported lower levels of total PGs (24.5 mg g^−1^ dw of the herb) in the methanol extract of *T. montanum* from Macedonian flora, including caerulescenoside (7.0 mg g^−1^ dw), cas-tanoside A (1.1. mg g^−1^ dw), echinacoside (2.4 mg g^−1^ dw), forsythoside B (10.2 mg g^−1^ dw), verbascoside (2.0 mg g^−1^ dw), and samioside (1.7 mg g^−1^ dw), which were detected and quantified with LC/DAD/ESI-MSn. In this study, β-OH-forsythoside B, jionoside A, teupolioside, and stachysoside A were detected for the first time in *T. montanum*.

Considering the content of individual PGs in the samples from different microlocations, it can be seen that HAE extracts of T2, T3 and T4 are characterized by a remarkable content of echinacoside (23.54 mg g^−1^ dw), poliumoside (21.72 mg g^−1^ dw), and teupolioside (19.56 mg g^−1^ dw) while T6 proves to be an outstanding source of both teupolioside (21.02 mg g^−1^ dw) and stachysoside A (21.33 mg g^−1^ dw). When compared to the contents of major PGs in various *Cistanche* spp. from commercial sources in the global herbal market, it is evident that the analyzed contents of selected PGs in *T. montanum* are in a similar range, *e.g.*, verbascoside and echinacoside dominated in *C. deserticola* (0.8–31.4 mg g^−1^ and 2.3–27.1 mg g^−1^ dw, respectively) [63] and *C. salsa* (9.44 mg g^−1^ and 10.98 mg g^−1^, respectively) [64] while poliumoside was the most abundant compound in *C. sinensis* (3.4–36.2 mg g^−1^) [65].

Although the flavonoid subclass has been described as the largest group of phenolic compounds in *Teucrium* spp., only three flavone glycosides were detected in the extracts of HAE and MAE in this study: vicenin-2 (apigenin-6,8-C-di-C-glucopyranoside), diosmin (diosmetin-7-*O*-rutinoside), and acacetin-7-*O*-rutinoside. These compounds were quantified in significantly lower amounts than the previously mentioned PGs. Vicenin-2 accounted for 0.36–0.70 mg g^−1^ dw and 0.27–0.75 mg g^−1^ dw in HAE and MAE extracts, respectively. Diosmin (HAE: 0.37–0.55 mg g^−1^ dw, MAE: 0.33–0.58 mg g^−1^ dw) and acacetin-7-*O*-rutinoside (HAE: 0.15–0.46 mg g^−1^ dw, MAE: 0.16–0.54 mg g^−1^ dw) were also present at similar levels. Mitreski et al. (2014) [21] also reported a lower content of total flavonoids (2.2 mg g^−1^ dw) in the *T. montanum* extract compared to PGs content, with rutin, luteolin, and apigenin-7-*O*-glycosides being the most abundant compounds. As for the quantification of flavonoids in SWE extracts, it was found that SWE did not promote the extraction of these relatively nonpolar compounds. In contrast, Nastić et al. (2018a) [20] reported that naringin (996 mg 100 g^−1^ per dry extract-DE), rutin (125 mg 100 g^−1^ per DE), and epicatechin (120 mg 100 g^−1^ per DE) were the most abundant compounds in the extracts. It has been previously reported that flavonoids containing glucose or rhamnose as well as flavonoids with dominant hydroxyl groups have higher extraction efficiency at a relatively low temperature than their aglycone forms due to their strong hydrogen bonding with water. In addition, glycosylated flavonoids have a lower melting point and higher molecular weight, making them more easily degraded at high temperatures (>160 °C) [66,67]. A similar parallel observation could be made for the lower extraction efficiency of PGs with SWE since both glycosidic bonds and hydroxyl groups are predominant in their structures.

It is obvious that the HPLC-UV-DAD analysis showed a discrepancy between the results of quantified PGs and flavonoids, and the results of TPC/antioxidant capacity in SWE extracts, indicating the presence of other compounds with antioxidant capacity. This result could be explained by several mechanisms: (1) hydrolysis of compounds extracted from the native matrix, producing fragments involved in neoformation processes; (2) decomposition processes, producing degradation products with preserved antioxidant capacity; and (3) Maillard and caramelization reactions, producing compounds with preserved or even higher antioxidant capacity [68,69].

### 3.5. PCA Analysis

**Figure 7:**
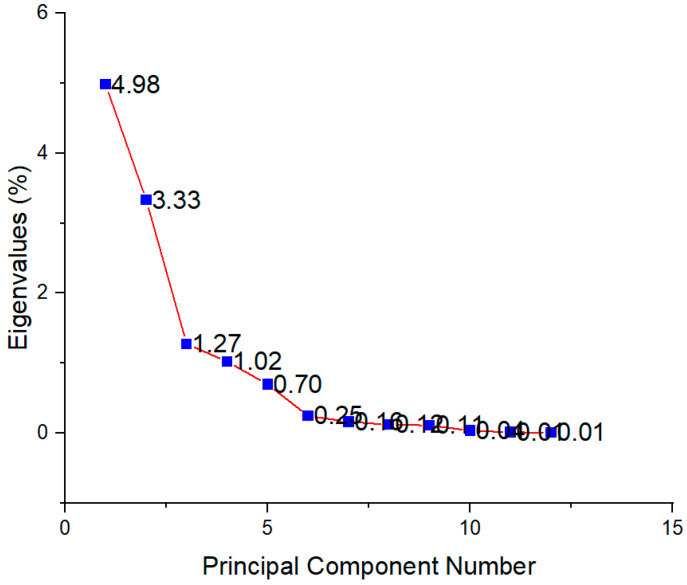
Scree plot showing eigenvalues for each principal component.

In order to determine the principal components with integrated maximum variance from the original variables for further discrimination of the studied *T. montanum* samples from different microsites, PCA was performed. As can be seen in Figure 7, the first three principal components account for 78.91% of the total variance (PC1 = 41.14%, PC2 = 27.49%, PC3 = 10.28%).

**Figure 8:**
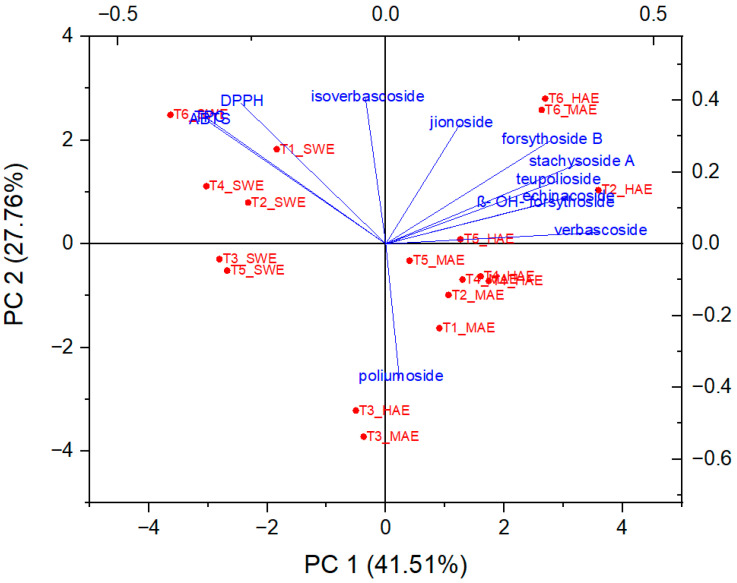
Biplot representing PC scores and loading vectors.

**Figure 9:**
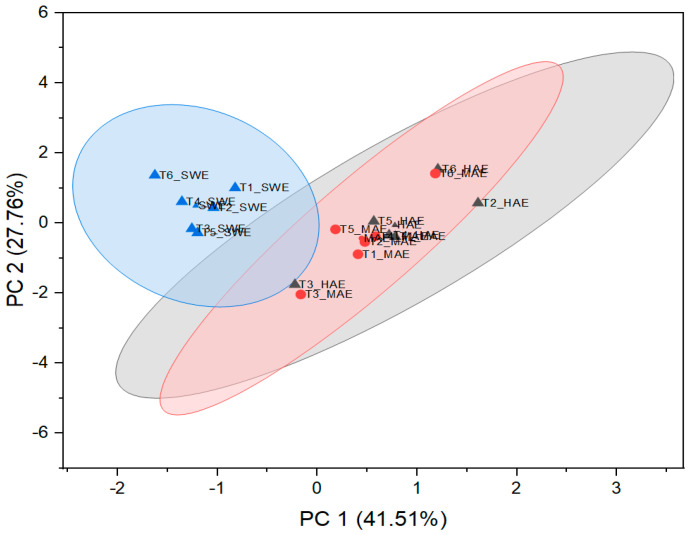
Visualization of standardized scores with cluster plot with confidence ellipse.

As can be seen from Figure 8 and Figure 9, PC1 correlates strongly with the most dominant PGs and thus has relatively high loadings for echinacoside (0.34), teupolioside (0.32), stachysosyde A (0.36), and verbascoside (0.40). In contrast, PC2 is strongly associated with the levels of TPC (0.35), ABTS (0.34), and DPPH (0.39).

From the biplot, it appears that the plant samples were indirectly grouped by the extraction technique used and not by the microlocation. SWE extracts have higher values in PC2, distinguishing them from HAE and MAE, based on the bioactive reaction variables measured spectrophotometrically. This trend is expected because the TPC values and antioxidant capacity results do not follow the trend of quantified caffeic acid derivatives in SWE extracts. These results are related to the negative correlation between the TPC, DPPH, ABTS values, and the individual phenolic compounds since their amount in the SWE samples is significantly lower than in the extracts from HAE and MAE.

On the other hand, important experimental variables describing all six samples of HAE and MAE are PGs, with verbascoside, stachysoside A, and forsythoside B having the greatest influence on PC1, as their charges are furthest from the origin PC.

Samples T3 and T6, originating from both MAE and HAE, proved to be the most pronounced among all samples. These observations are related to the highest poliumoside content in T3, along with verbascoside, stachysoside A, and forsythoside B in T6. In addition, Figure 9 confirms the clustering of the *T. montanum* samples with respect to the extraction techniques used, indicating the importance of similar dependent variables for each cluster, *i.e.*, PGs as the main contributors to the bioactive potential of the extracts of HAE and MAE and TPC, DPPH, and ABTS for SWE extracts.

## 4. Conclusions

Regarding the advanced extraction procedures used, SWE showed the highest TPC values and antioxidant capacities of all *T. montanum* samples (TPC: 84.50–109.55 mg eq GA g^−^^1^ dw; ABTS: 0.402–0.547 mmol eq Trolox g^−^^1^ dw, DPPH: 0.336–0.427 mmol eq Trolox g^−^^1^ dw). However, HAE proved to be the most successful in recovering total PGs (30.36–68.06 mg g^−^^1^ dw). In addition, the potential of MAE in terms of the extraction performance (25.88–58.88 mg g^−^^1^ dw) should not be neglected, especially considering its short extraction time (HAE: 30 min, MAE: 9 min). In this study, stachysoside A and teupolioside were detected for the first time in *T. montanum* L. in appreciable amounts. Interestingly, flavonoids, the most abundant polyphenolic group found so far in *Teucrium* sp., were found in negligible amounts. A PCA analysis classified HAE and MAE with PC1, which strongly correlates with the quantified PGs, *i.e.*, echinacoside, teupolioside, and stachysoside A, whereas all SWE extracts were mainly described with PC2, indicating the greatest influence of TPC, ABTS, and DPPH values on the bioactive potential assessment. Considering the maximum content of extracted PGs (T2_HAE = 6.8% dw), it is evident that *T. montanum* L. could serve as an exceptional source of PGs for many purposes, including biotechnological production of plant extracts and pharmaceuticals or for enhancement in functional foods.

## Figures and Tables

**Table 1:** Results of TPC and AC measured with ABTS and DPPH in HAE, MAE, and SWE extracts.

Extraction Technique	Sample	TPC (mg eq GA g^−1^ dw *)	ABTS (mmol eq Trolox g^−1^ dw **)	DPPH (mmol eq Trolox g^−1^ dw **)
HAE	T1	50.10 ± 0.06 ^a^	0.233 ± 0.00 ^a^	0.209 ± 0.00 ^a^
MAE		46.52 ± 0.74 ^b^	0.206 ± 0.00 ^b^	0.171 ± 0.00 ^a^
SWE		88.55 ± 1.81 ^ab^	0.412 ± 0.00 ^ab^	0.358 ± 0.01 ^a^
HAE	T2	52.78 ± 1.44 ^a^	0.244 ± 0.01 ^a^	0.230 ± 0.01 ^a^
MAE		47.75 ± 1.13 ^b^	0.219 ± 0.00 ^b^	0.204 ± 0.01 ^b^
SWE		92.36 ± 1.81 ^ab^	0.406 ± 0.01 ^ab^	0.366 ± 0.00 ^ab^
HAE	T3	46.16 ± 0.25 ^a^	0.221 ± 0.00 ^a^	0.209 ± 0.00 ^a^
MAE		48.26 ± 0.06 ^b^	0.218 ± 0.00 ^b^	0.211 ± 0.00 ^b^
SWE		88.98 ± 0.17 ^ab^	0.402 ± 0.01 ^ab^	0.336 ± 0.00 ^ab^
HAE	T4	49.49 ± 0.78 ^a^	0.261 ± 0.01 ^a^	0.231 ± 0.01 ^a^
MAE		47.38 ± 0.82 ^b^	0.223 ± 0.01 ^a^	0.196 ± 0.00 ^a^
SWE		96.18 ± 1.32 ^ab^	0.450 ± 0.01 ^a^	0.368 ± 0.00 ^a^
HAE	T5	55.14 ± 0.78 ^a^	0.246 ± 0.00 ^a^	0.292 ± 0.00 ^a^
MAE		48.29 ± 0.35 ^a^	0.265 ± 0.01 ^b^	0.317 ± 0.00 ^b^
SWE		84.50 ± 0.33 ^a^	0.458 ± 0.00 ^ab^	0.353 ± 0.00 ^ab^
HAE	T6	71.60 ±1.27 ^a^	0.350 ± 0.01 ^a^	0.338 ± 0.00 ^a^
MAE		71.80 ± 0.82 ^b^	0.329 ± 0.01 ^b^	0.331 ± 0.00 ^b^
SWE		109.55 ± 0.66 ^ab^	0.547 ± 0.01 ^ab^	0.427 ± 0.00 ^ab^

* expressed in milligrams of gallic acid equivalents per gram of dry matter; ** expressed in mmol of Trolox equivalents per gram of dry matter; HAE—heat-assisted extraction; MAE—microwave-assisted extraction; SWE—subcritical water extraction. Values marked with the same superscript letters (^“a”^ and ^“b”^) in the same column within the same sample are significantly different (*p* < 0.05).

**Table 2:** Correlations between measured variables expressed by Pearson’s correlation coefficients.

	HAE				MAE				SWE		
	TPC	DPPH	ABTS		TPC	DPPH	ABTS		TPC	DPPH	ABTS
TPC	1			TPC	1			TPC	1		
DPPH	0.93	1		DPPH	0.70	1		DPPH	0.93	1	
ABTS	0.95	0.85	1	ABTS	0.92	0.92	1	ABTS	0.80	0.90	1

HAE—heat-assisted extraction; MAE—microwave-assisted extraction; SWE—subcritical water extraction.

**Table 4:** UHPLC-HR MS/MS analysis of reference standards.

Reference Standard	Molecular Formula	Error (ppm)	Calculated Mass (*m*/*z*)	Observed Mass [M-H]^−^ (*m*/*z*)	MS/MS (*m*/*z*) and Abundance of Each Fragment Ion (%)
echinacoside	C_35_H_46_O_20_	2.80	785.2510	785.2532	MS^2^ [785.2510]: 623.2200 (2.7), 477.1603 (2.9), 161.0245 (100)
verbascoside	C_29_H_36_O_15_	2.40	623.1981	623.1996	MS^2^ [623.1996]: 461.1665 (7.9), 315.1081 (2.5), 161.0248 (100)

**Table 5:** Identified polyphenolic compounds in HAE, MAE, and SWE extracts quantified by HPLC-UV-DAD.

		mg g^−1^ dw *											
Extraction Technique	Sample	Vicenin-2 ^1^	β-OH-Isoforshythoside ^2^	Echinacoside	Jionoside A ^2^	Teupolioside ^2^	Stachysoside A ^2^	Poliumoside ^2^	Verbascoside	Forsythoside B ^2^	Isoverbascoside ^3^	Diosmin ^4^	Acacetin-7-*O*-Rutinoside ^5^
HAE	T1	0.71 ± 0.03	0.57 ± 0.01	9.10 ± 0.20 ^a^	1.00 ± 0.04	4.23 ± 0.04	10.57 ± 0.07 ^a^	1.02 ± 0.02	4.42 ± 0.07 ^ab^	2.51 ± 0.07	/	0.45 ± 0.04	0.40 ± 0.01
MAE		0.69 ± 0.04	0.78 ± 0.05	6.33 ± 0.41 ^a^	1.09 ± 0.03	3.03 ± 0.17	5.59 ± 0.04 ^a^	0.78 ± 0.01	2.78 ± 0.01 ^a^	0.94 ± 0.12	/	0.39 ± 0.00	0.37 ± 0.03
SWE		/	/	2.94 ± 0.71 ^a^	1.40 ± 0.55	/	1.06 ± 0.49 ^a^	/	1.61 ± 0.60 ^b^	2.12 ± 1.34	2.09 ± 0.91	/	/
HAE	T2	0.58 ± 0.03 ^a^	0.85 ± 0.03	23.54 ± 1.10 ^a^	1.48 ± 0.10	6.93 ± 0.42 ^a^	13.39 ± 0.83 ^ab^	9.14 ± 0.39 ^a^	7.90 ± 0.46 ^a^	2.82 ± 0.33	2.01 ± 0.24	0.55 ± 0.10	0.37 ± 0.02
MAE		0.27 ± 0.02 ^a^	1.44 ± 0.09	11.94 ± 0.08 ^a^	1.48 ± 0.04	4.67 ± 0.04 ^b^	0.69 ± 0.01 ^a^	7.55 ± 0.24 ^a^	3.72 ± 0.06 ^a^	/	1.28 ± 0.07	0.38 ± 0.07	0.17 ± 0.01
SWE		/	/	3.97 ± 0.11 ^a^	1.20 ± 0.20	1.22 ± 0.63 ^ab^	0.87 ± 0.11 ^b^	1.89 ± 0.08 ^a^	0.96 ± 0.07 ^a^	0.45 ± 0.09	1.04 ± 0.07	0.43 ± 0.02	/
HAE	T3	/	/	0.69 ± 0.05	/	2.08 ± 0.02	1.91 ± 0.06	21.72 ± 0.22 ^a^	3.13 ± 0.06 ^a^	/	0.83 ± 0.15	0.39 ± 0.01	/
MAE		/	/	1.81 ± 0.29	/	2.71 ± 0.06	1.90 ± 0.01	24.55 ± 0.76 ^a^	3.20 ± 0.09 ^b^	/	/	0.53 ± 0.02	/
SWE		/	/	/	/	/	1.20 ± 0.05	4.33 ± 0.13 ^a^	1.06 ± 0.05 ^ab^	/	1.29 ± 0.06	0.33 ± 0.02	/
HAE	T4	0.36 ± 0.01	1.01 ± 0.01 ^a^	6.66 ± 0.08 ^b^	0.77 ± 0.02	19.56 ± 0.70 ^a^	8.02 ± 0.18 ^a^	0.63 ± 0.01	3.35 ± 0.08 ^a^	1.33 ± 0.00	/	0.44 ± 0.02	/
MAE		0.34 ± 0.00	3.08 ± 0.44 ^a^	5.48 ± 0.22 ^a^	1.75 ± 0.12	8.79 ± 1.51 ^a^	3.55 ± 0.63 ^a^	0.80 ± 0.01	1.87 ± 0.23 ^a^	/	0.56 ± 0.09	0.53 ± 0.10	/
SWE		/	/	1.95 ± 0.03 ^ab^	1.35 ± 0.06	/	/	/	/	/	1.32 ± 0.08	0.61 ± 0.02	/
HAE	T5	0.57 ± 0.02 ^a^	0.61 ± 0.28	8.90 ± 0.22 ^b^	0.83 ± 0.06	4.60 ± 0.12	8.89 ± 0.13 ^a^	0.59 ± 0.01	6.28 ± 0.10 ^a^	1.58 ± 0.03	1.09 ± 0.05	0.37 ± 0.07	0.15 ± 0.02
MAE		0.70 ± 0.00 ^a^	/	9.43 ± 0.06 ^a^	1.19 ± 0.02	3.49 ± 0.02	5.26 ± 0.15 ^a^	0.74 ± 0.03	4.31 ± 0.07 ^a^	0.94 ± 0.01	0.55 ± 0.01	0.42 ± 0.08	0.16 ± 0.00
SWE		/	/	2.94 ± 0.27 ^ab^	/	/	/	/	1.84 ± 0.05 ^a^	/	/	/	0.27 ± 0.11
HAE	T6	0.70 ± 0.01	1.43 ± 0.01 ^a^	8.57 ± 0.21 ^b^	0.97 ± 0.03	21.02 ± 0.86 ^a^	21.33 ± 0.78 ^a^	1.06 ± 0.03	6.00 ± 0.19	3.66 ± 0.05	1.84 ± 0.06	0.45 ± 0.03	0.46 ± 0.02
MAE		0.75 ± 0.01	2.66 ± 0.01 ^a^	8.78 ± 0.00 ^a^	1.72 ± 0.11	17.39 ± 0.04 ^a^	17.55 ± 0.17 ^a^	1.20 ± 0.19	5.50 ± 0.20	2.61 ± 0.10	1.47 ± 0.16	0.58 ± 0.01	0.54 ± 0.00
SWE		/	/	3.10 ± 0.91 ^ab^	1.44 ± 0.70	1.28 ± 0.76 ^a^	/	/	/	/	2.16 ± 1.10	0.66 ± 0.26	/

* Results are expressed in mg per g of dry weight of sample; HAE—heat-assisted extraction, MAE—microwave-assisted extraction, SWE—subcritical water extraction; */*: not quantified; ^1^ quantified as mg eq AP g^−1^ dw; ^2^ quantified as mg eq ECH g^−1^ dw; ^3^ quantified as mg eq VERB g^−1^ dw; ^4^ quantified as mg eq DS g^−1^ dw; ^5^ quantified as mg eq AC g^−1^ dw. Values marked with the same superscript letters in the same column within the same sample are significantly different (*p* < 0.05).

## Data Availability

Data is contained within the article and Supplementary Material.

## Supplementary Materials

The following supporting information can be downloaded at: https://www.mdpi.com/article/10.3390/antiox12111903/s1.
