# Supplementary material for: Teucrium montanum L.—Unrecognized Source of Phenylethanoid Glycosides: Green Extraction Approach and Elucidation of Phenolic Compounds via NMR and UHPLC-HR MS/MS
Source: Antioxidants (Basel). 2023 Oct 24;12(11):1903. doi: 10.3390/antiox12111903 (PMC10669637; doi:10.3390/antiox12111903)
Supplement: Supplementary file 1 [file antioxidants-12-01903-s001.zip › antioxidants-2659615-supplementary.pdf]

Supplementary Materials

Figure S1: ^{1}H spectrum, structure, numbering and full assignment of **echinacoside** in methanol-d_4 at 25 °C 2
Figure S2: ^{13}C spectrum, structure, numbering and full assignment of **echinacoside** in methanol-d_4 at 25 °C....3
Figure S3: COSY spectrum of **echinacoside** in methanol-d_4 at 25 °C 4
Figure S4: ^{1}H-^{13}C HSQC spectrum of **echinacoside** in methanol-d_4 at 25 °C 5
Figure S5: ^{1}H-^{13}C HMBC spectrum of **echinacoside** in methanol-d_4 at 25 °C 6
Figure S6: Fully assigned NOESY spectrum of **echinacoside** in methanol-d_4 at 25 °C 7
Figure S7: ^{1}H spectrum, structure, numbering and full assignment of **verbascoside** in methanol-d_4 at 25 °C.....8
Figure S8: ^{13}C spectrum, structure, numbering and full assignment of **verbascoside** in methanol-d_4 at 25 °C....9
Figure S9: COSY spectrum of **verbascoside** in methanol-d_4 at 25 °C 10
Figure S10: ^{1}H-^{13}C HSQC spectrum of **verbascoside** in methanol-d_4 at 25 °C 11
Figure S11: ^{1}H-^{13}C HMBC spectrum of **verbascoside** in methanol-d_4 at 25 °C 12
Figure S12: ^{1}H spectrum, structure, numbering and full assignment of **PH5** in methanol-d_4 at 25 °C 13
Figure S13: ^{13}C spectrum, structure, numbering and full assignment of **PH5** in methanol-d_4 at 25 °C 14
Figure S14: COSY spectrum of **PH5** in methanol-d_4 at 25 °C 15
Figure S15: ^{1}H-^{13}C HSQC spectrum of **PH5** in methanol-d_4 at 25 °C 16
Figure S16: ^{1}H-^{13}C HMBC spectrum of **PH5** in methanol-d_4 at 25 °C 17
Figure S17: Fully assigned NOESY spectrum of **PH5** in methanol-d_4 at 25 °C 18
Figure S18: ^{1}H spectrum, structure, numbering and full assignment of **PH6** in methanol-d_4 at 25 °C 19
Figure S19: ^{13}C spectrum, structure, numbering and full assignment of **PH6** in methanol-d_4 at 25 °C 20
Figure S20: COSY spectrum of **PH6** in methanol-d_4 at 25 °C 21
Figure S21: ^{1}H-^{13}C HSQC spectrum of **PH6** in methanol-d_4 at 25 °C 22
Figure S22: ^{1}H-^{13}C HMBC spectrum of **PH6** in methanol-d_4 at 25 °C 23
Figure S23: Fully assigned NOESY spectrum of **PH6** in methanol-d_4 at 25 °C 24
Figure S24: ^{1}H spectrum, structure, numbering and full assignment of **PH7** in methanol-d_4 at 25 °C 25
Figure S25: ^{13}C spectrum, structure, numbering and full assignment of **PH7** in methanol-d_4 at 25 °C 26
Figure S26: COSY spectrum of **PH7** in methanol-d_4 at 25 °C 27
Figure S27: ^{1}H-^{13}C HSQC spectrum of **PH7** in methanol-d_4 at 25 °C 28
Figure S28: ^{1}H-^{13}C HMBC spectrum of **PH7** in methanol-d_4 at 25 °C 29
Figure S29: Fully assigned NOESY spectrum of **PH7** in methanol-d_4 at 25 °C 30
Table S1: Comparison of proton chemical shifts for all analyzed compounds in methanol- d_4 at 25 °C, the biggest differences marked with superscripted mark " * "........31
Table S2: Comparison of carbon chemical shifts for all analyzed compounds in methanol- d_4 at 25 °C, the biggest differences marked with superscripted mark " * "........32
Table S3: Comparison of coupling constants $^{n}J_{H,H}$ (Hz) for all analyzed compounds in methanol-d_4 at 25 °C, the biggest differences marked with superscripted mark " * "........33

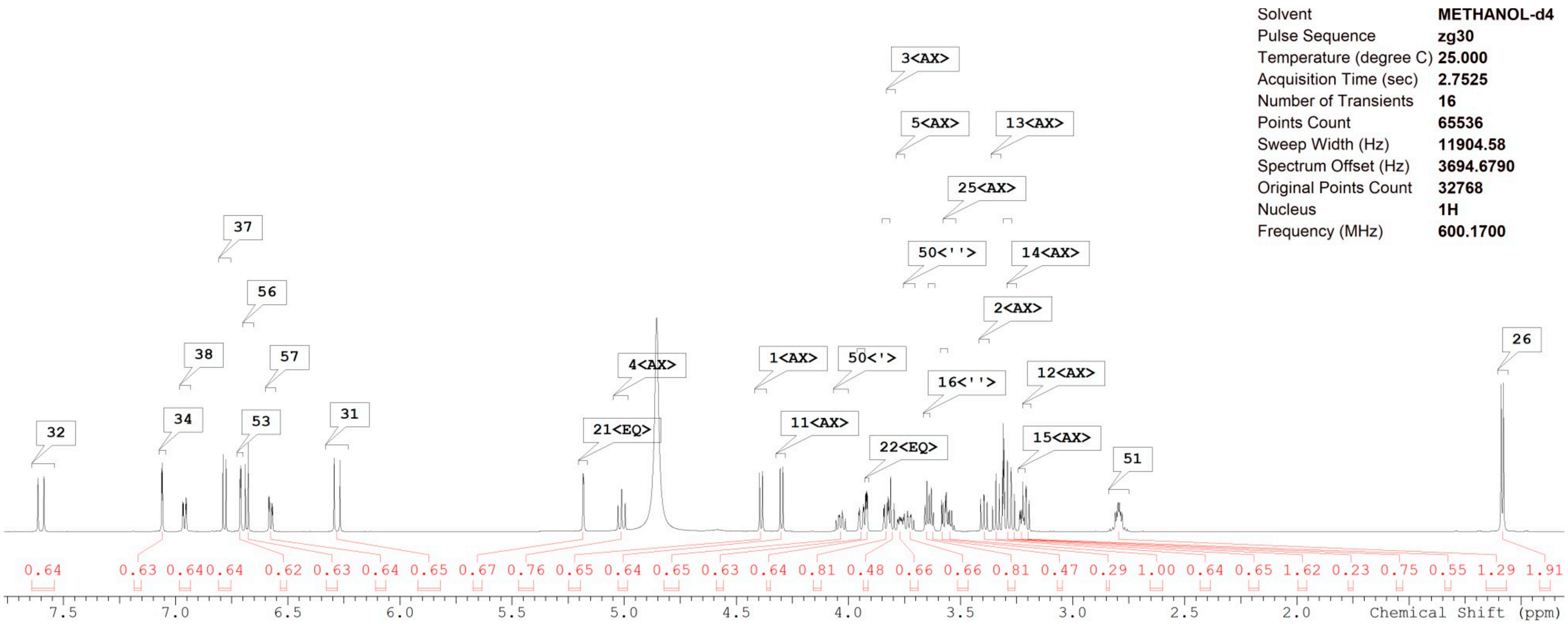

Shift1 (ppm)	H's	Type	J (Hz)	Atom1	Multiplet1	Shift1 (ppm)	H's	Type	J (Hz)	Atom1	Multiplet1
1.085	3.000	d	6.2	26	M31	3.920	1.000	dd	3.3, 1.8	22<EQ>	M15
2.796	2.000	m	-	51	M20	3.943	1.000	dd	11.4, 2.2	6<'>	M14
3.209	1.000	dd	9.3, 7.8	12<AX>	M26	4.035	1.000	ddd	9.6, 8.1, 7.0	50<'>	M13
3.223	1.000	ddd	9.4, 5.4, 2.2	15<AX>	M30	4.299	1.000	d	7.6	11<AX>	M12
3.276	1.000	t	8.9	14<AX>	M28	4.390	1.000	d	7.8	1<AX>	M11
3.292	1.000	t	9.6	24<AX>	M29	5.012	1.000	t	9.6	4<AX>	M10
3.343	1.000	t	9.1	13<AX>	M27	5.183	1.000	d	1.6	21<EQ>	M09
3.396	1.000	dd	9.1, 8.0	2<AX>	M25	6.281	1.000	d	16.0	31	M02
3.553	1.000	dq	9.6, 6.2	25<AX>	M24	6.577	1.000	dd	8.1, 2.1	57	M08
3.574	1.000	m	9.4, 3.3	23<AX>	M23	6.683	1.000	d	8.0	56	M07
3.636	1.000	t	11.4, 5.4	6<">	M19	6.712	1.000	d	2.2	53	M06
3.645	1.000	t	11.9, 5.6	16<">	M22	6.783	1.000	d	8.2	37	M05
3.730	1.000	m	9.6, 8.2, 6.7	50<">	M18	6.961	1.000	dd	8.3, 2.1	38	M04
3.767	1.000	ddd	9.6, 5.4, 2.2	5<AX>	M21	7.061	1.000	d	2.0	34	M03
3.812	1.000	br t	9.3	3<AX>	M16	7.602	1.000	d	15.8	32	M01
3.832	1.000	dd	11.9, 2.3	16<'>	M17						

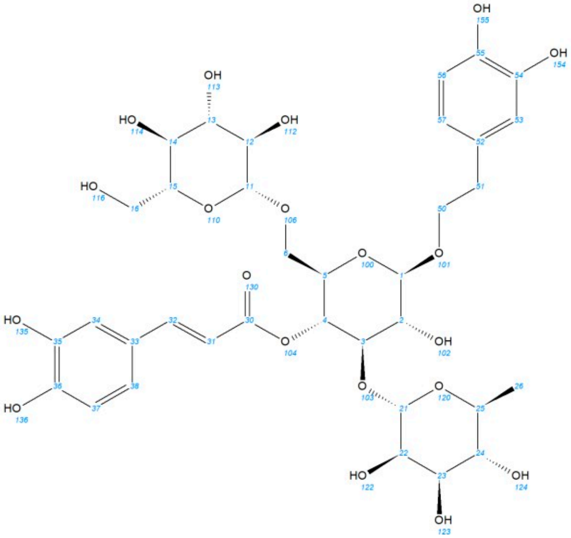

Figure S1: ^{1}H spectrum, structure, numbering and full assignment of **echinacoside** in methanol-d$_4$ at 25 °C

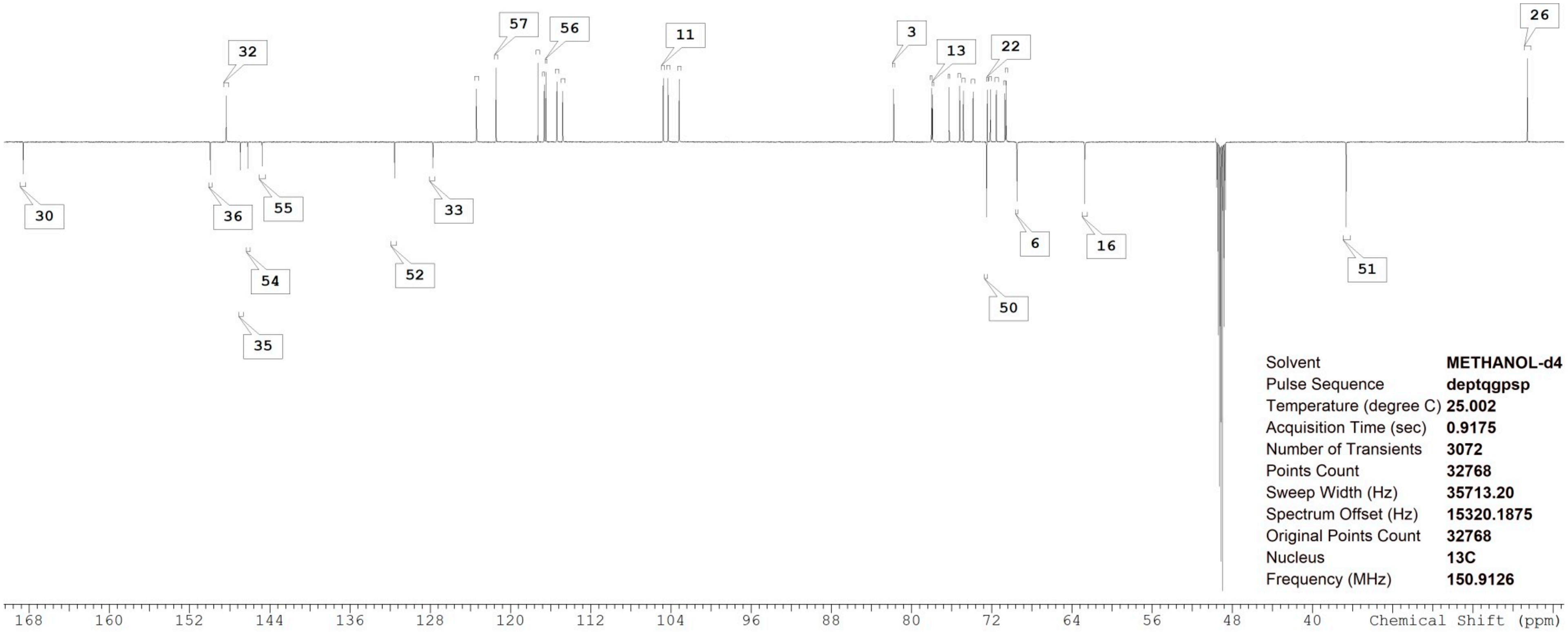

Shift1 (ppm)	C's	Type	Atom1	Multiplet1
18.589	1.000	s	26	M35
36.680	1.000	s	51	M34
62.745	1.000	s	16	M33
69.498	1.000	s	6	M32
70.574	1.000	s	25	M30
70.696	1.000	s	4	M29
71.563	1.000	s	14	M28
72.163	1.000	s	23	M27
72.466	1.000	s	22	M26
72.524	1.000	s	50	M31
73.881	1.000	s	24	M25
74.849	1.000	s	5	M24
75.210	1.000	s	12	M23
76.257	1.000	s	2	M22
77.919	1.000	s	13	M21
78.020	1.000	s	15	M20
81.790	1.000	s	3	M19
103.196	1.000	s	21	M18
104.301	1.000	s	1	M17
104.778	1.000	s	11	M16
114.809	1.000	s	31	M15
115.387	1.000	s	34	M14
116.478	1.000	s	56	M13
116.651	1.000	s	37	M12
117.279	1.000	s	53	M11
121.454	1.000	s	57	M10
123.411	1.000	s	38	M09
127.759	1.000	s	33	M07
131.579	1.000	s	52	M06
144.788	1.000	s	55	M05
146.218	1.000	s	54	M04
146.955	1.000	s	35	M03
148.371	1.000	s	32	M08
149.967	1.000	s	36	M02
168.614	1.000	s	30	M01

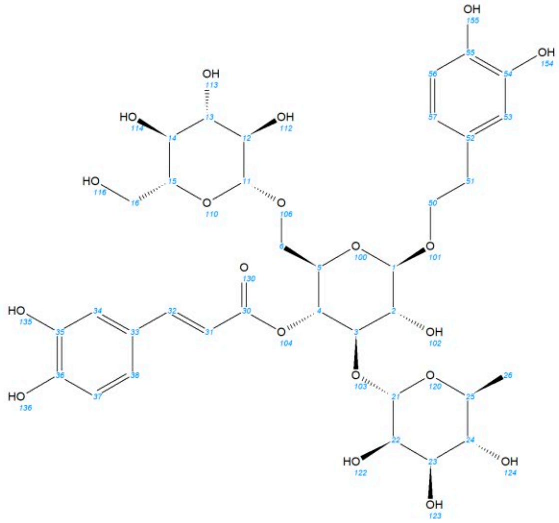

Figure S2: ^{13}C spectrum, structure, numbering and full assignment of **echinacoside** in methanol-d_4 at 25 °C

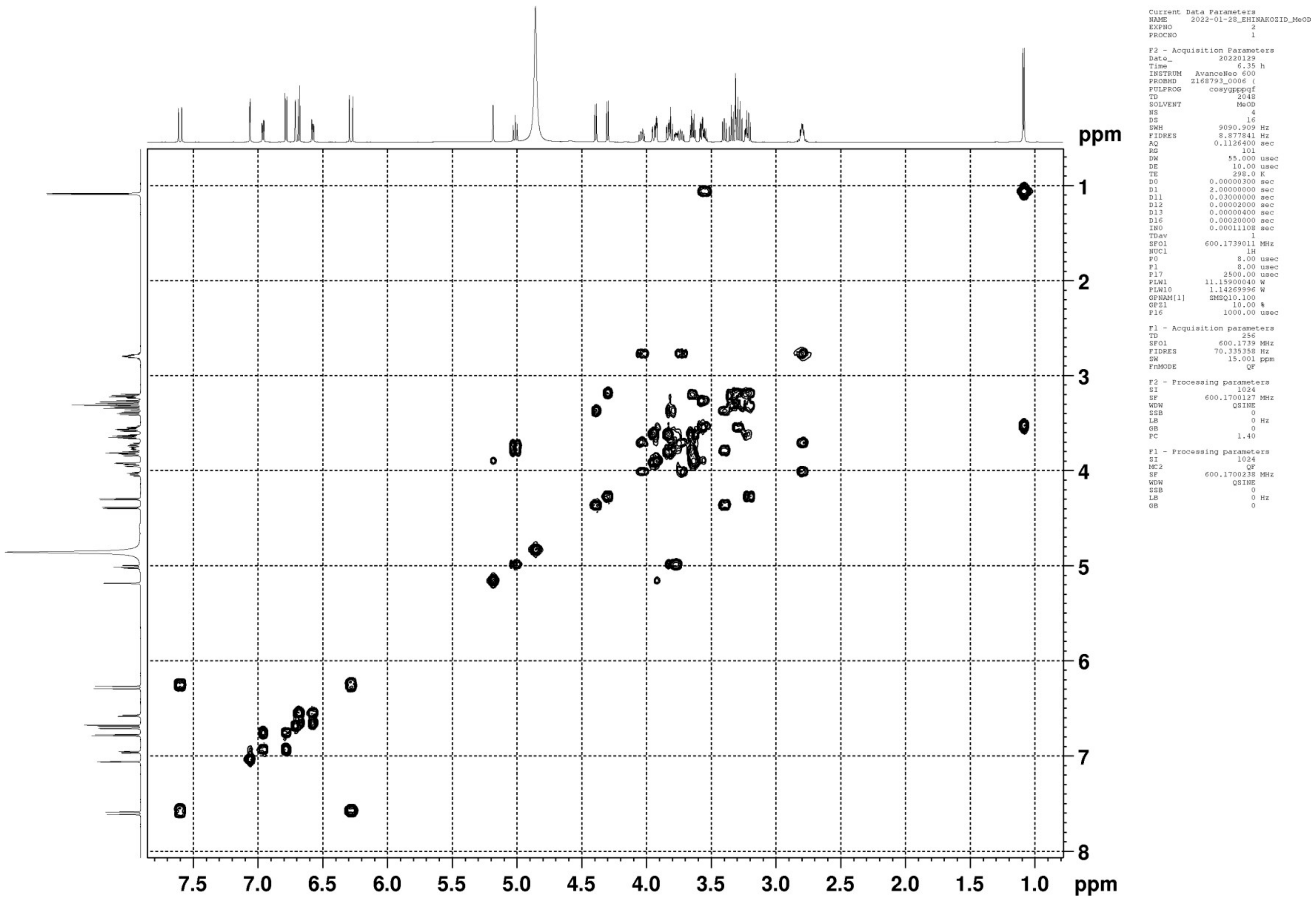

Figure S3: COSY spectrum of **echinacoside** in methanol-d_4 at 25 °C

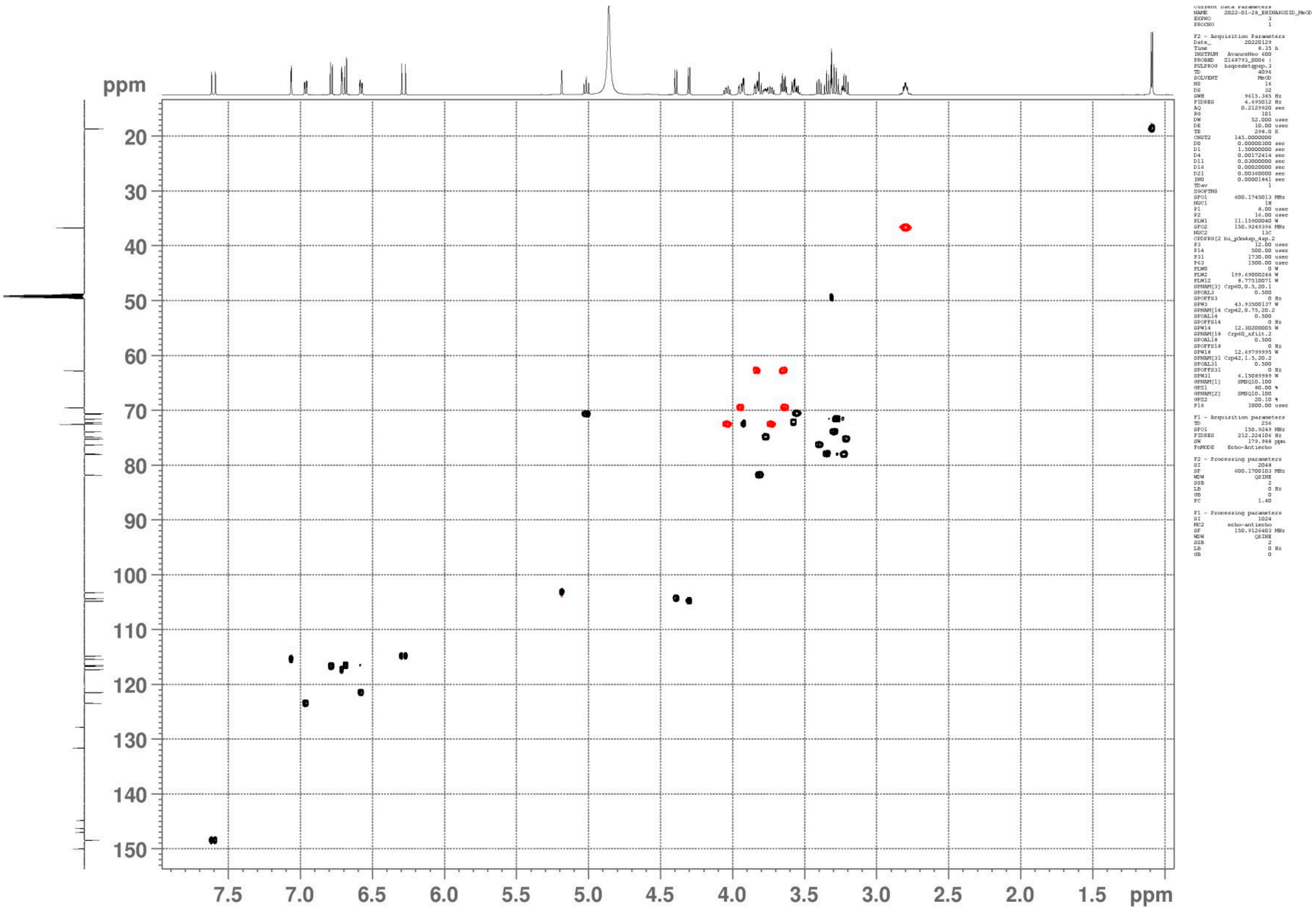

Figure S4: ^{1}H-^{13}C HSQC spectrum of **echinacoside** in methanol-d$_4$ at 25 °C

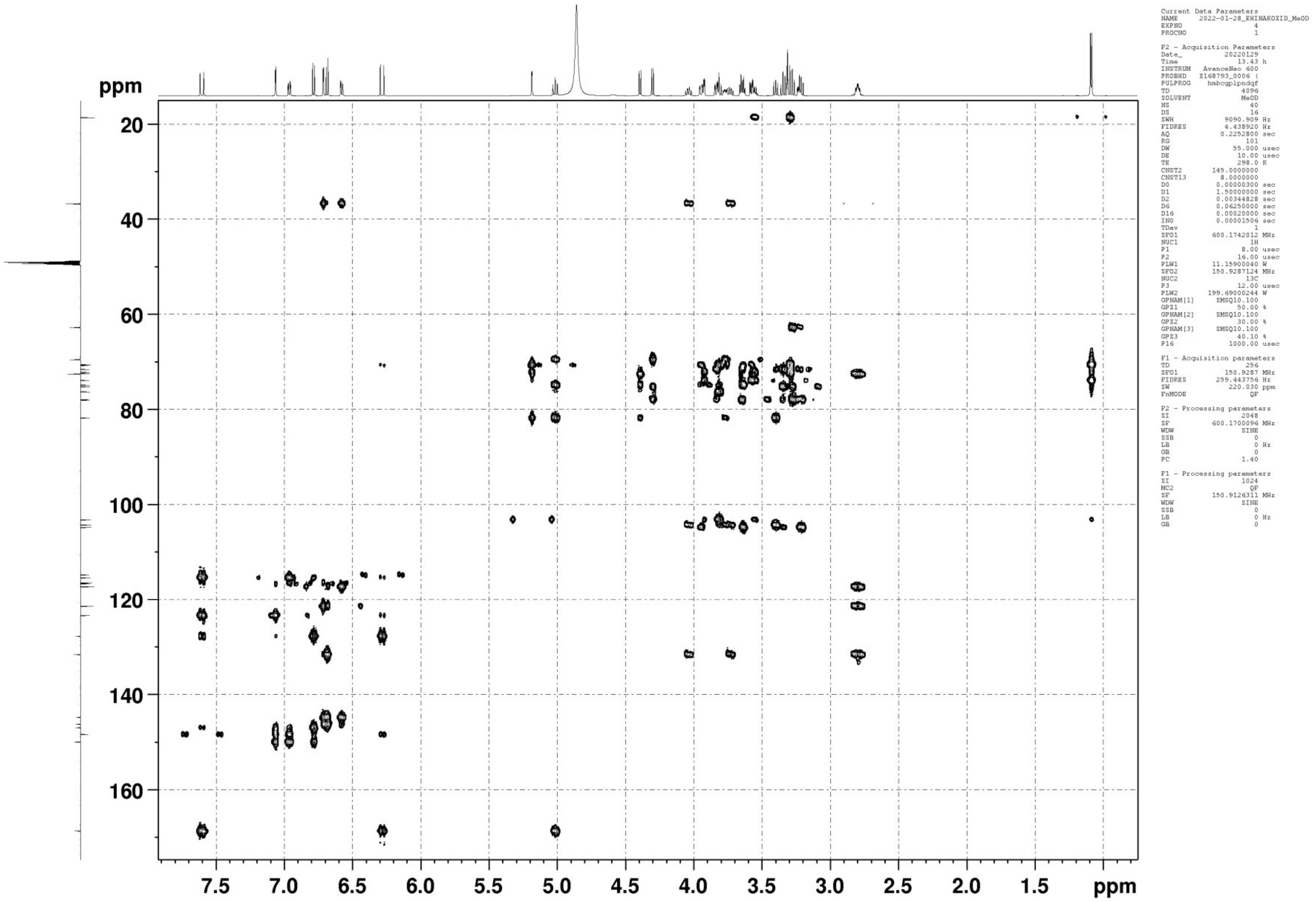

Figure S5: ^{1}H-^{13}C HMBC spectrum of **echinacoside** in methanol-d$_4$ at 25 °C

Acquisition Time (sec)	(0.0860, 0.0213)
Comment	Z168793_0006 (CPP1.1 TCI 600S3 H&F-C/N-D-05 Z XT)
Frequency (MHz)	(600.1700, 600.1700)
Nucleus	(1H, 1H)
Origin	AvanceNeo 600
Original Points Count	(1024, 256)
Points Count	(2048, 1024)
Pulse Sequence	roesyphpr.2
Solvent	METHANOL-d4
Sweep Width (Hz)	(11898.95, 11991.28)

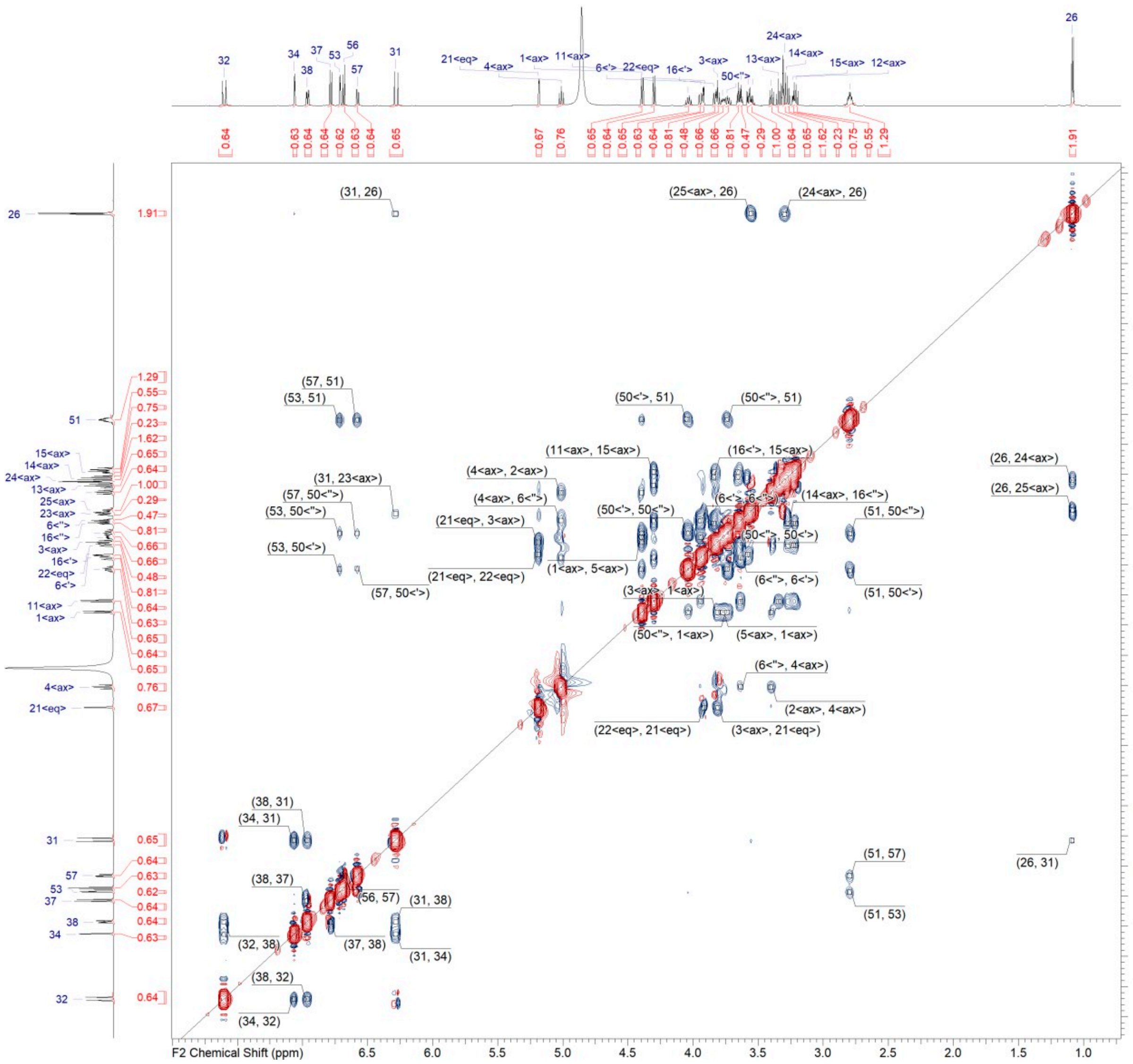

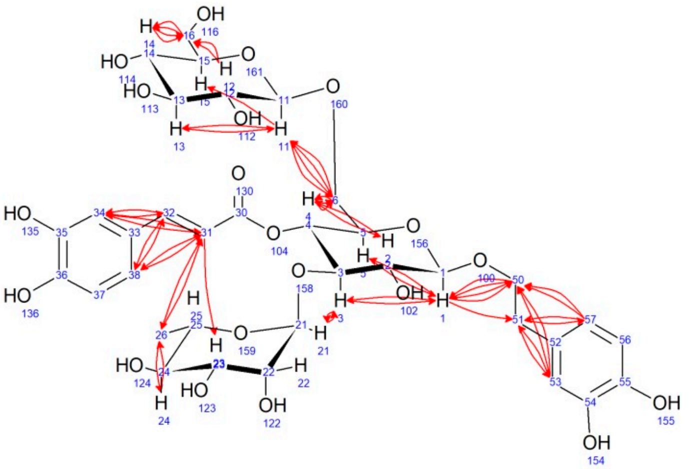

Figure S6: Fully assigned NOESY spectrum of **echinacoside** in methanol-d4 at 25 °C

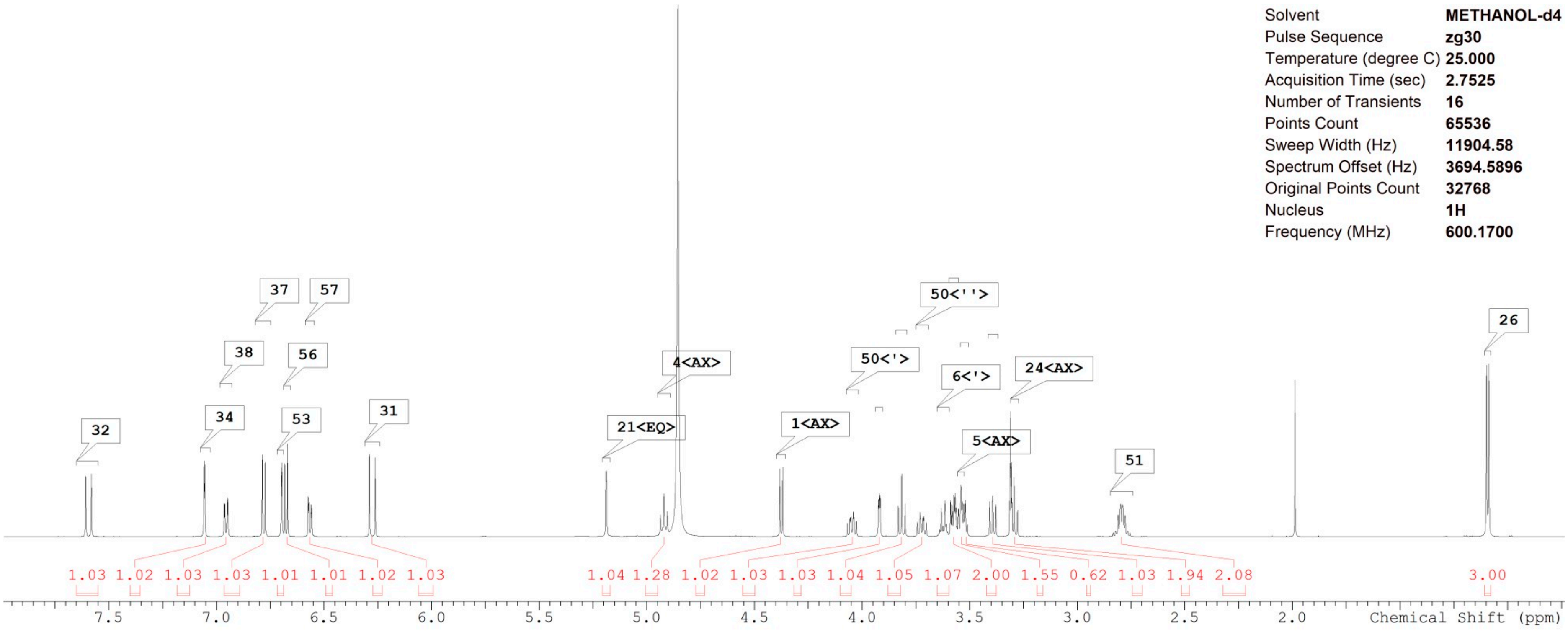

Shift1 (ppm)	H's	Type	J (Hz)	Atom1	Multiplet1	Shift1 (ppm)	H's	Type	J (Hz)	Atom1	Multiplet1
1.094	3.000	d	6.2	26	M19	6.276	1.000	d	15.8	31	M08
2.794	2.000	m	-	51	M17	6.566	1.000	dd	8.1, 2.1	57	M07
3.294	1.000	t	9.6	24<AX>	M18	6.677	1.000	d	8.0	56	M06
3.393	1.000	dd	9.2, 7.9	2<AX>	M20	6.698	1.000	d	2.0	53	M05
3.524	1.000	dd	10.2, 5.8	6<">	M21	6.780	1.000	d	8.2	37	M04
3.542	1.000	m	-	5<AX>	M22	6.956	1.000	dd	8.2, 2.0	38	M03
3.572	2.000	m	-	23<AX>, 25<AX>	M23	7.057	1.000	d	2.0	34	M02
3.624	1.000	d	10.0	6<'>	M16	7.595	1.000	d	15.8	32	M01
3.723	1.000	ddd	9.6, 8.3, 6.8	50<">	M15						
3.816	1.000	t	9.3	3<AX>	M14						
3.920	1.000	dd	3.2, 1.7	22<EQ>	M13						
4.047	1.000	ddd	9.5, 8.3, 6.7	50<'>	M12						
4.376	1.000	d	7.8	1<AX>	M11						
4.921	1.000	br t	9.4	4<AX>	M10						
5.189	1.000	d	1.6	21<EQ>	M09						

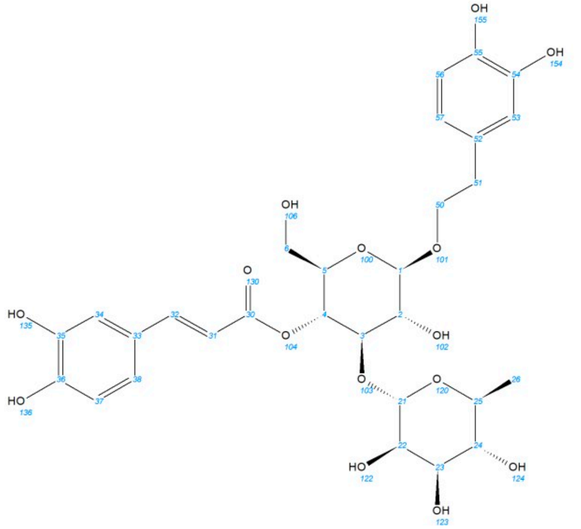

Figure S7: ^{1}H spectrum, structure, numbering and full assignment of **verbascoside** in methanol-d_4 at 25 °C

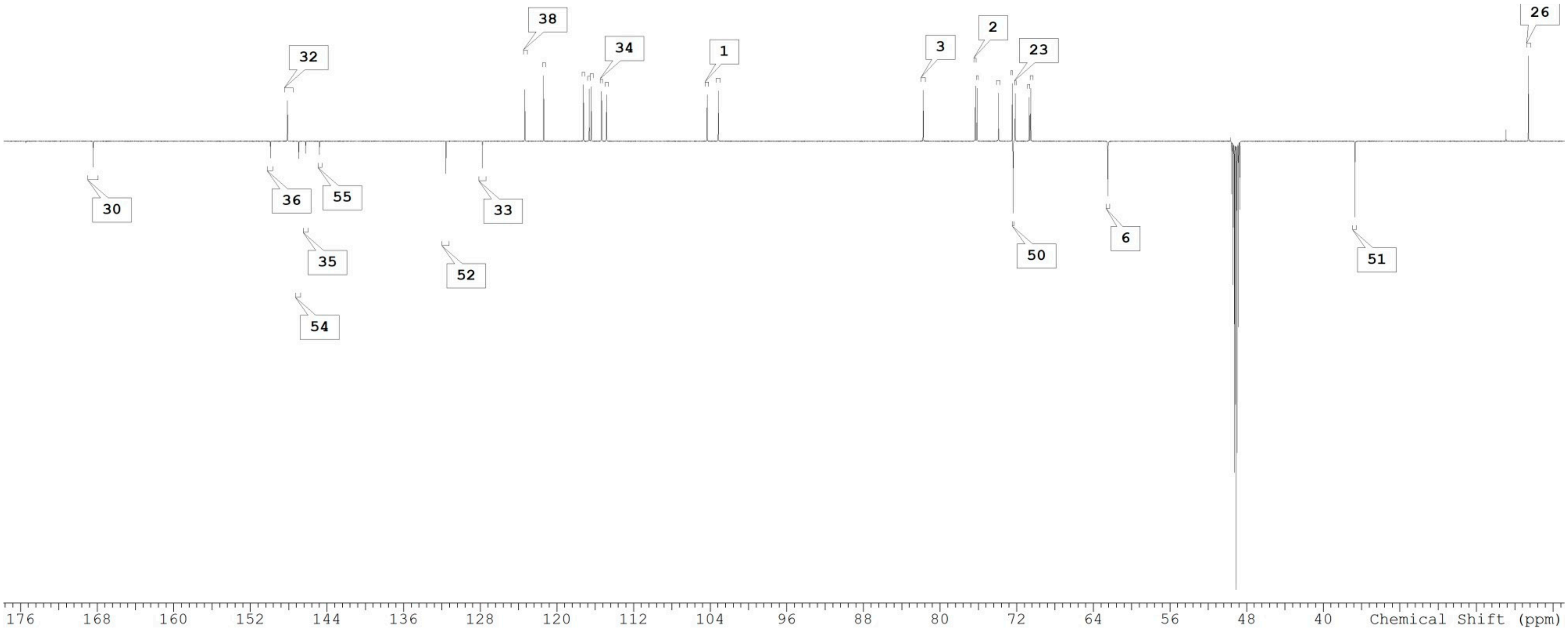

Shift1 (ppm)	C's	Type	Atom1	Multiplet1	Shift1 (ppm)	C's	Type	Atom1	Multiplet1
18.597	1.000	s	26	M29	115.352	1.000	s	34	M14
36.703	1.000	s	51	M28	116.436	1.000	s	56	M13
62.493	1.000	s	6	M27	116.645	1.000	s	37	M12
70.553	1.000	s	25	M25	117.244	1.000	s	53	M11
70.705	1.000	s	4	M24	121.397	1.000	s	57	M10
72.178	1.000	s	23	M23	123.362	1.000	s	38	M09
72.402	1.000	s	50	M26	127.789	1.000	s	33	M08
72.482	1.000	s	22	M22	131.595	1.000	s	52	M07
73.919	1.000	s	24	M21	144.811	1.000	s	55	M05
76.165	1.000	s	5	M20	146.263	1.000	s	35	M04
76.338	1.000	s	2	M19	146.964	1.000	s	54	M03
81.784	1.000	s	3	M18	148.155	1.000	s	32	M06
103.168	1.000	s	21	M17	149.925	1.000	s	36	M02
104.338	1.000	s	1	M16	168.428	1.000	s	30	M01
114.832	1.000	s	31	M15					

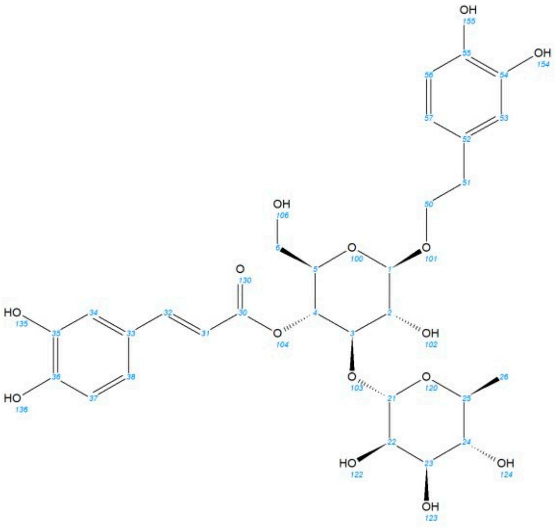

Solvent	**METHANOL-d4**
Pulse Sequence	**deptqgpsp**
Temperature (degree C)	**25.000**
Acquisition Time (sec)	**0.9175**
Number of Transients	**3072**
Points Count	**32768**
Sweep Width (Hz)	**35713.20**
Spectrum Offset (Hz)	**15322.5537**
Original Points Count	**32768**
Nucleus	**13C**
Frequency (MHz)	**150.9126**

Figure S8: ^{13}C spectrum, structure, numbering and full assignment of **verbascoside** in methanol-d_4 at 25 °C

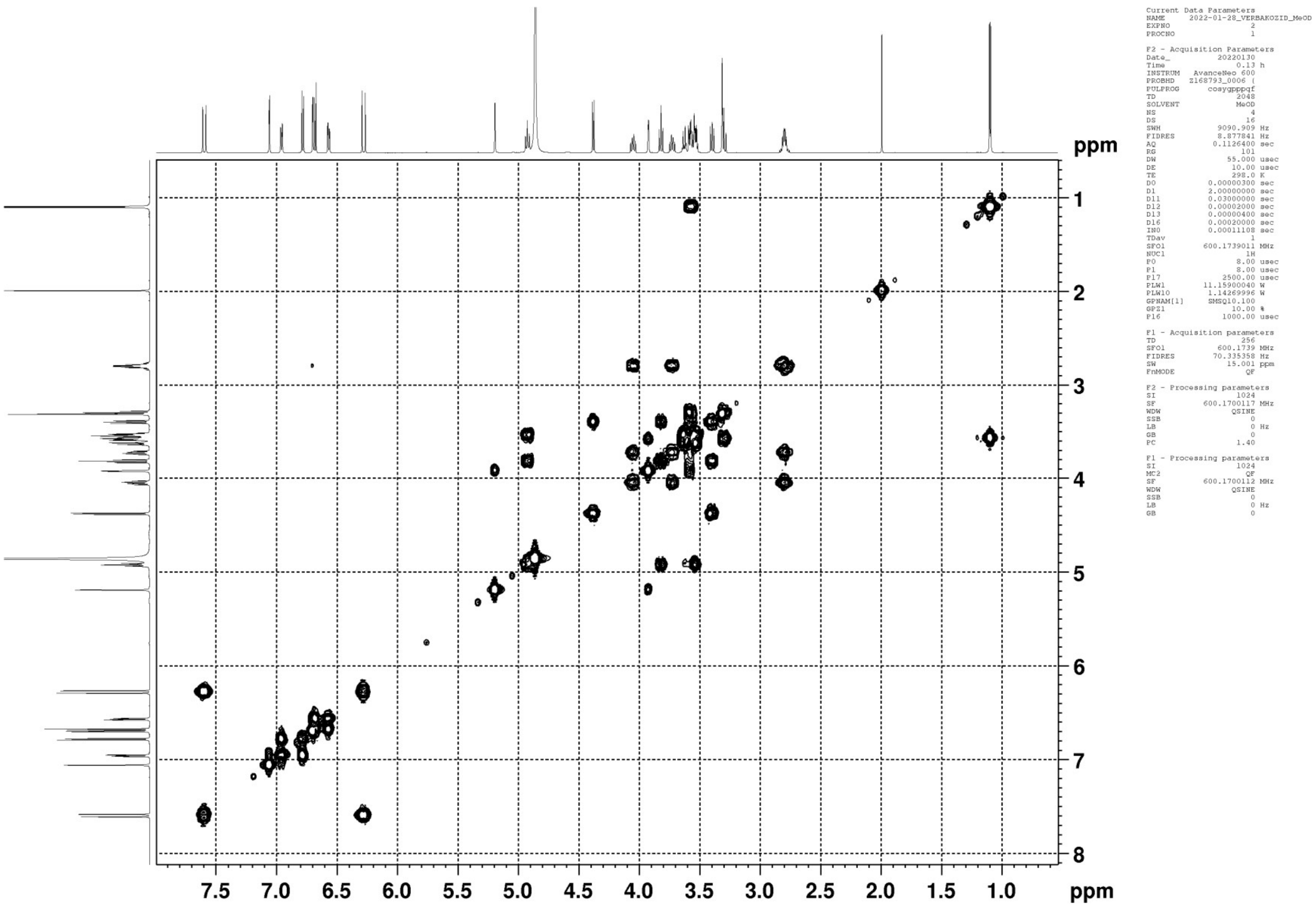

Figure S9: COSY spectrum of **verbascoside** in methanol-d_4 at 25 °C

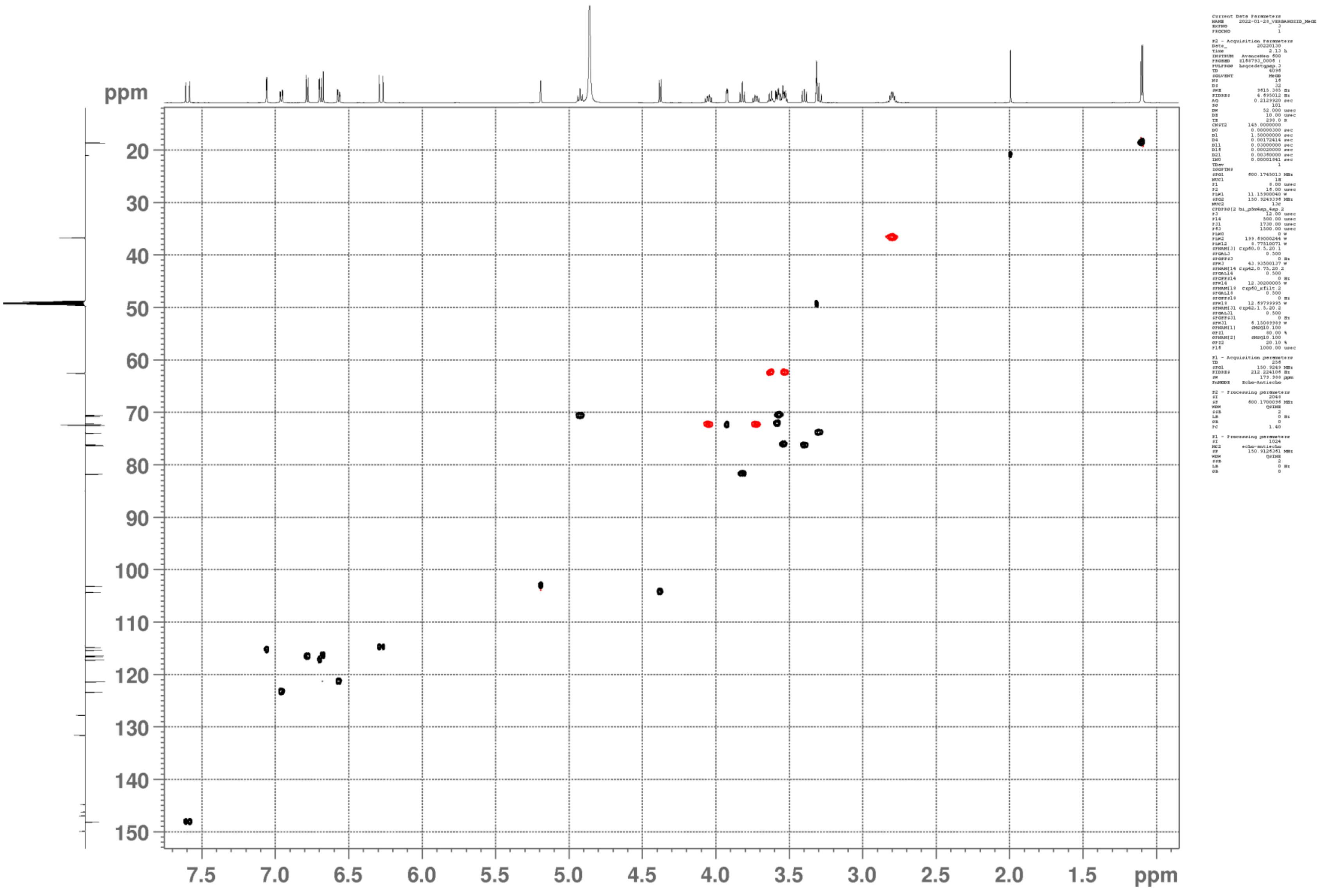

Figure S10: ^{1}H-^{13}C HSQC spectrum of **verbascoside** in methanol-d_4 at 25 °C

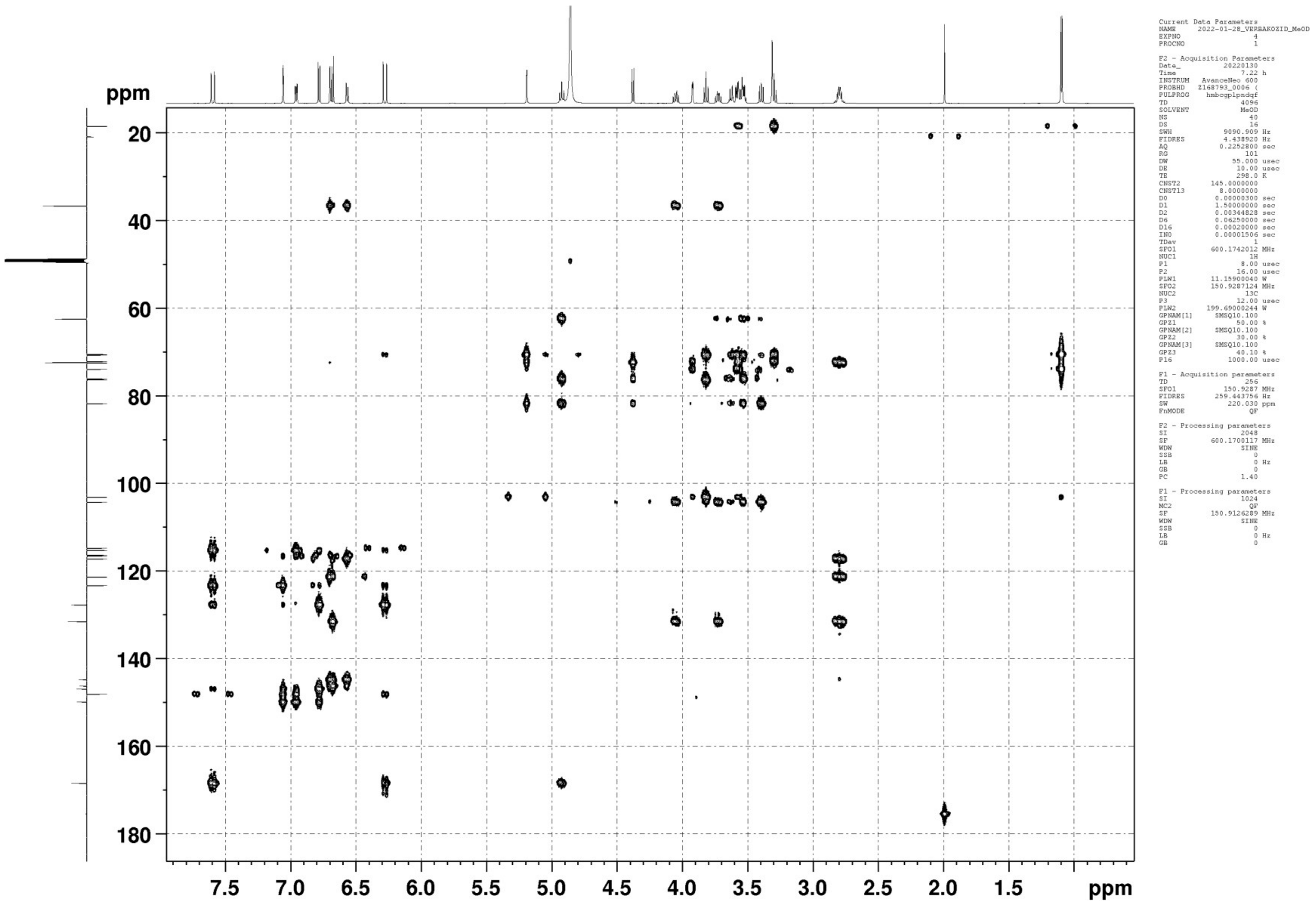

Figure S11: ^{1}H-^{13}C HMBC spectrum of **verbascoside** in methanol-d_4 at 25 °C

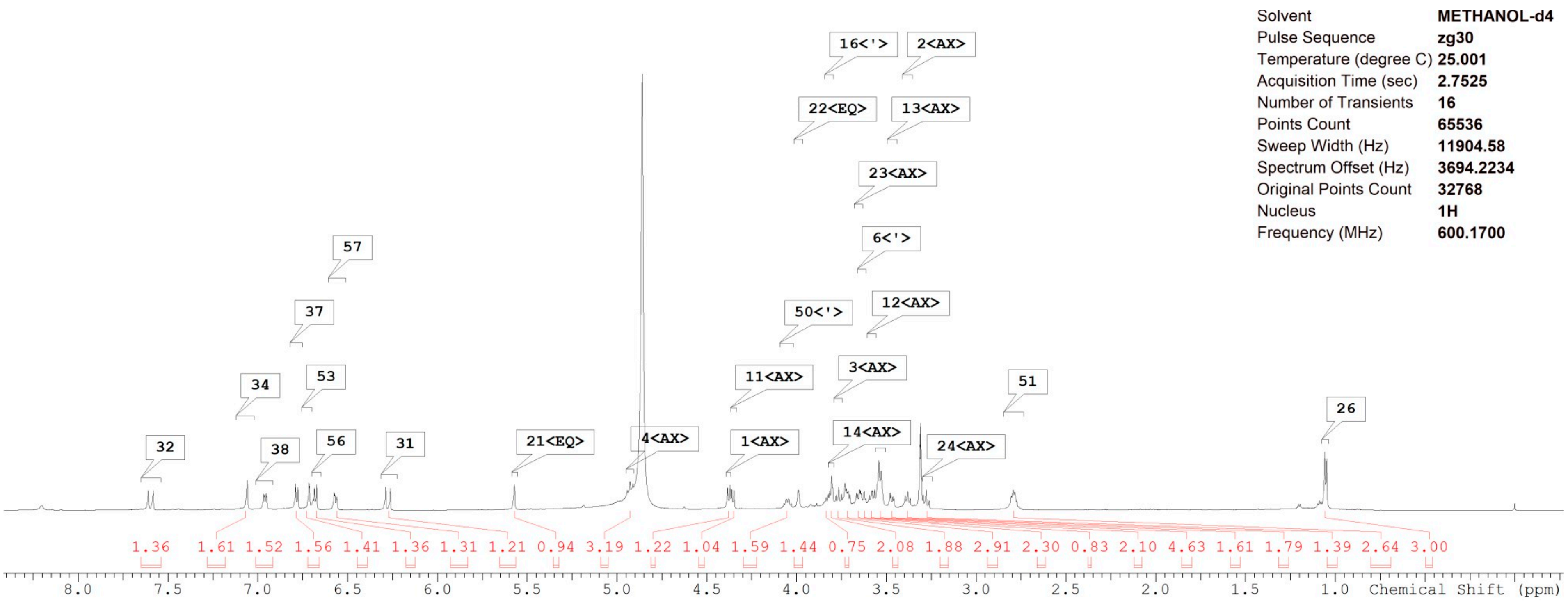

Shift1 (ppm)	H's	Type	J (Hz)	Atom1	Multiplet1
1.054	3.000	br d	6.0	26	M15
2.792	2.000	m	-	51	M16
3.279	1.000	br t	9.6	24<AX>	M17
3.382	1.000	br t	9.1	2<AX>	M18
3.470	1.000	br dd	9.7, 3.4	13<AX>	M19
3.536	4.000	m	-	5<AX>, 6<">, 25<AX>, 15<AX>	M21
3.582	1.000	br dd	9.6, 7.8	12<AX>	M20
3.635	1.000	br d	-	6<'>	M22
3.656	1.000	br dd	9.9, 3.4	23<AX>	M23
3.722	2.000	m	-	50<">, 16<">	M25
3.766	1.000	br t	9.3	3<AX>	M24
3.805	1.000	br s	3.4	14<AX>	M27
3.820	1.000	br dd	11.4, 7.3	16<'>	M26
3.990	1.000	br dd	3.1, 1.3	22<EQ>	M14
4.052	1.000	m	9.4, 7.8, 6.7	50<'>	M13

Shift1 (ppm)	H's	Type	J (Hz)	Atom1	Multiplet1
4.355	1.000	br d	7.6	11<AX>	M12
4.378	1.000	br d	8.0	1<AX>	M11
4.928	1.000	br t	9.3	4<AX>	M10
5.573	1.000	s	-	21<EQ>	M09
6.276	1.000	br d	15.8	31	M02
6.567	1.000	dd	8.0, 1.8	57	M08
6.680	1.000	br d	8.0	56	M07
6.714	1.000	br d	1.8	53	M06
6.784	1.000	br d	8.2	37	M05
6.959	1.000	br dd	8.2, 1.8	38	M04
7.059	1.000	br d	1.8	34	M03
7.598	1.000	br d	15.8	32	M01

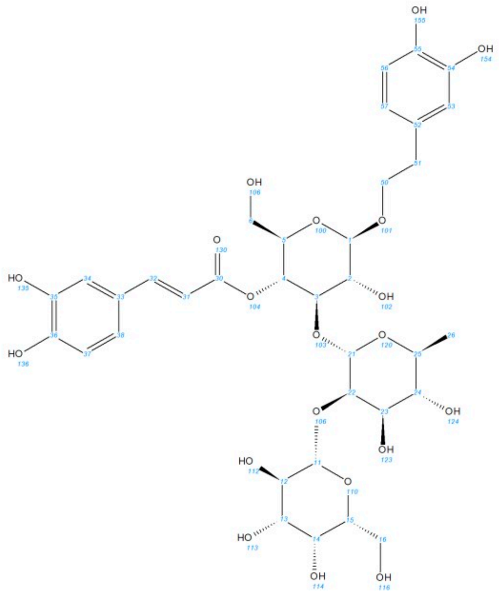

Figure S12: ^{1}H spectrum, structure, numbering and full assignment of **PH5** in methanol-d$_4$ at 25 °C

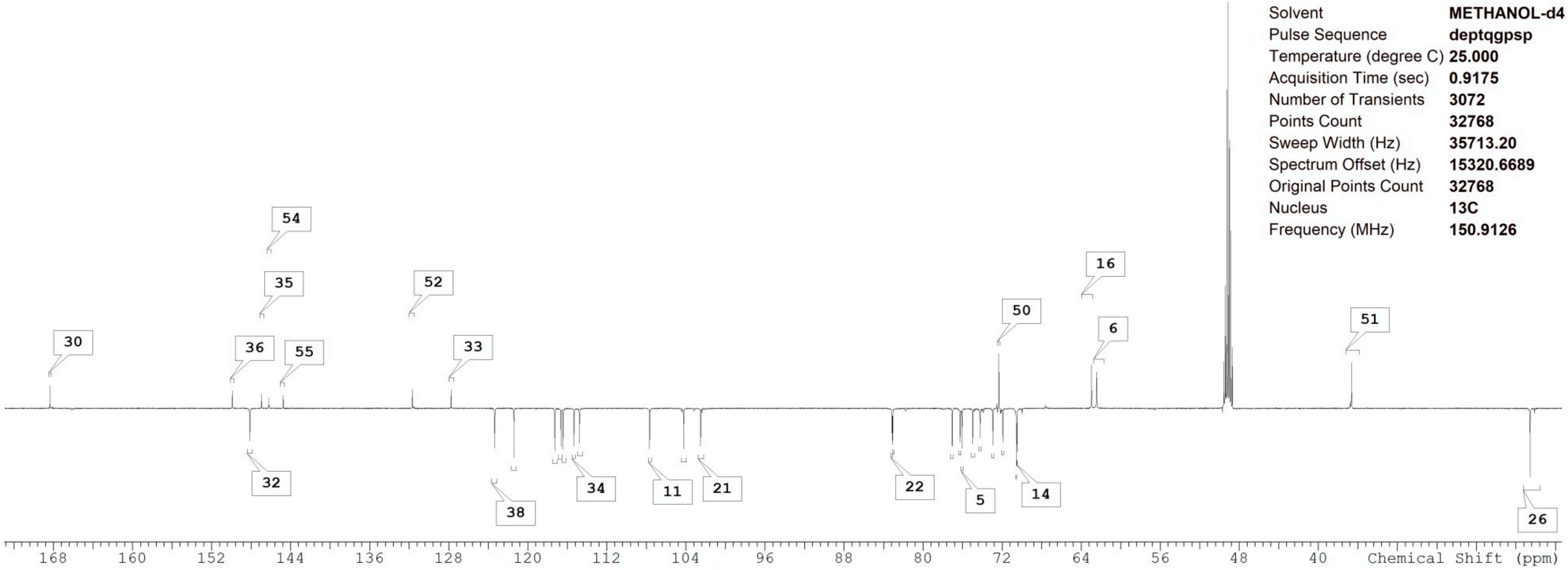

Shift1 (ppm)	C's	Type	Atom1	Multiplet1
18.577	1.000	s	26	M31
36.640	1.000	s	51	M32
62.445	1.000	s	6	M33
62.979	1.000	s	16	M34
70.497	1.000	s	14	M29
70.548	2.000	s	4, 25	M30
71.942	1.000	s	23	M27
72.346	1.000	s	50	M28
72.953	1.000	s	12	M26
74.246	1.000	s	24	M25
74.990	1.000	s	13	M24
76.073	1.000	s	5	M23
76.261	1.000	s	2	M22
77.070	1.000	s	15	M21
83.093	1.000	s	3	M20
83.151	1.000	s	22	M19
102.528	1.000	s	21	M18
104.239	1.000	s	1	M17
107.691	1.000	s	11	M16
114.784	1.000	s	31	M15
115.332	1.000	s	34	M14
116.445	1.000	s	56	M13
116.661	1.000	s	37	M12
117.290	1.000	s	53	M11
121.428	1.000	s	57	M10
123.363	1.000	s	38	M09
127.776	1.000	s	33	M07
131.698	1.000	s	52	M06
144.777	1.000	s	55	M05
146.222	1.000	s	54	M04
146.965	1.000	s	35	M03
148.143	1.000	s	32	M08
149.927	1.000	s	36	M02
168.379	1.000	s	30	M01

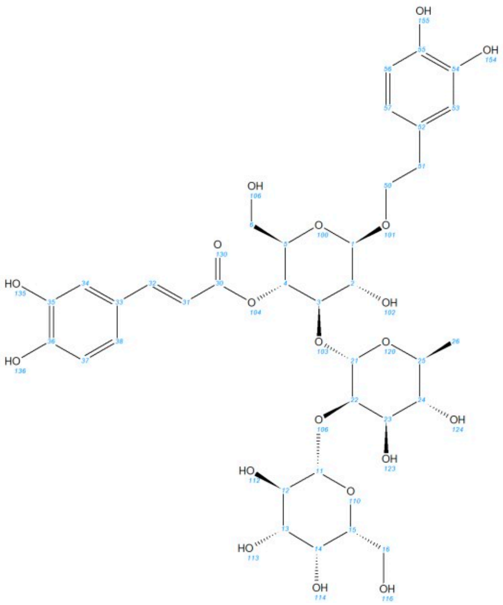

Figure S13: ^{13}C spectrum, structure, numbering and full assignment of **PH5** in methanol-d_4 at 25 °C

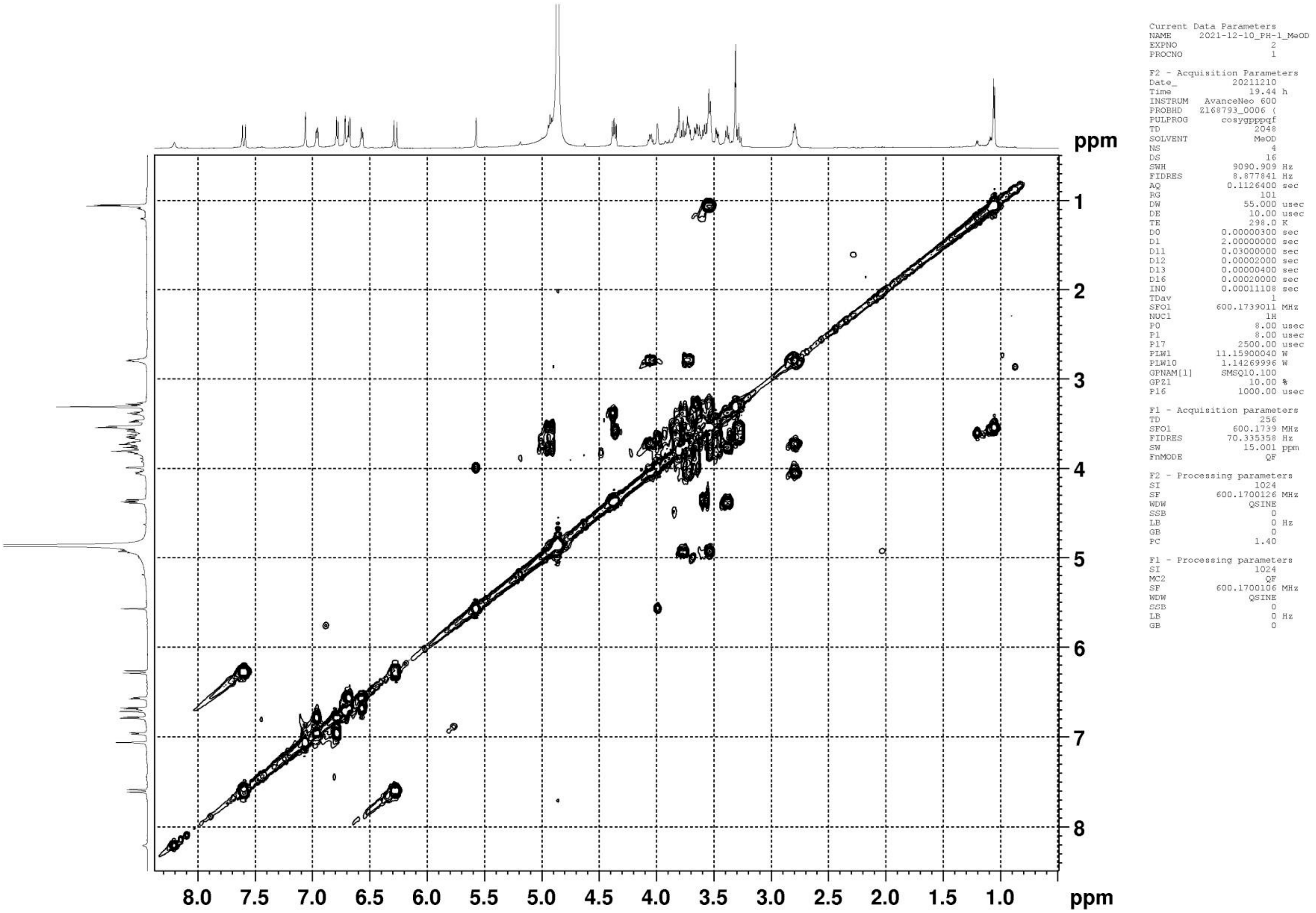

Figure S14: COSY spectrum of **PH5** in methanol-d_4 at 25 °C

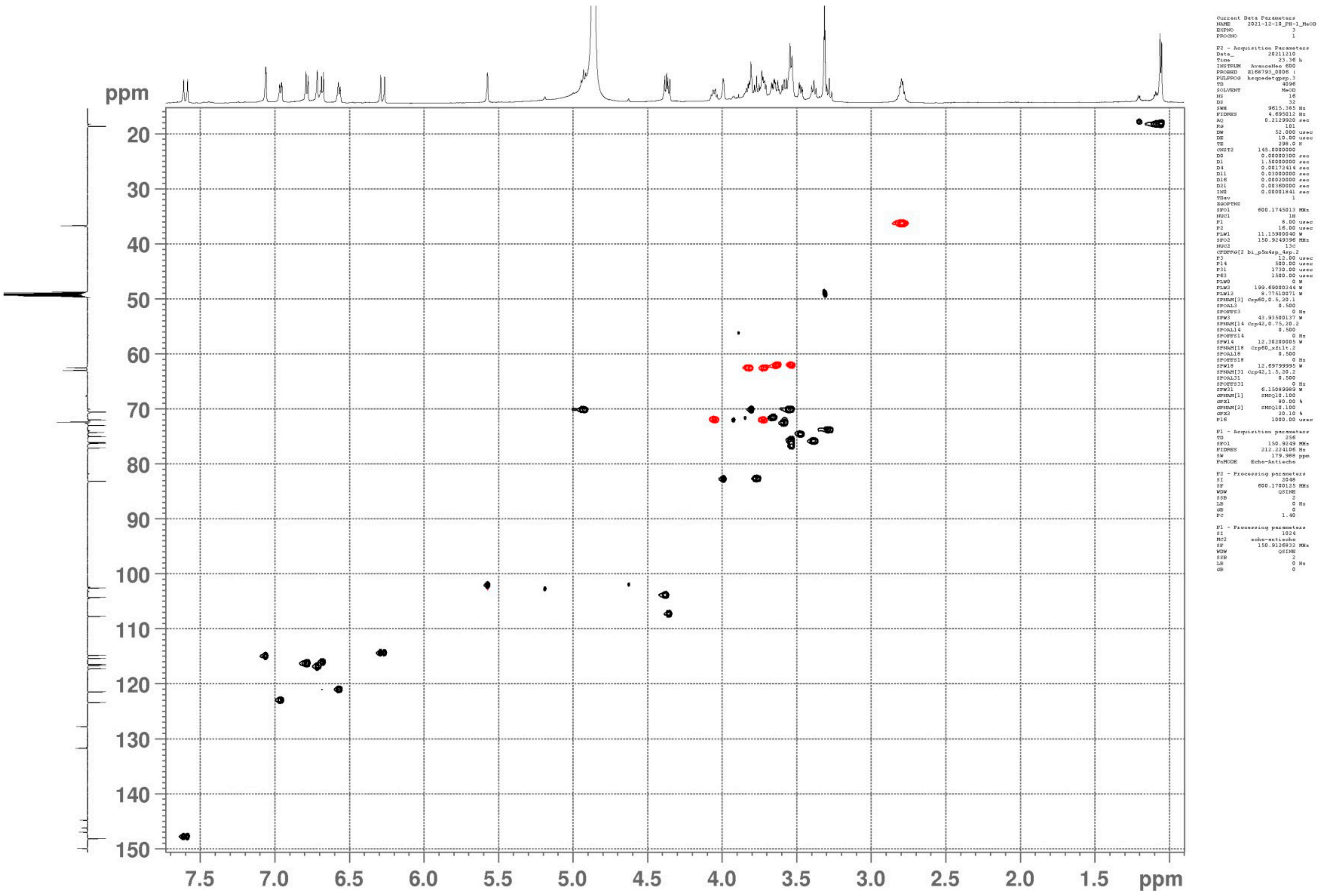

Figure S15: ^{1}H-^{13}C HSQC spectrum of **PH5** in methanol-d_4 at 25 °C

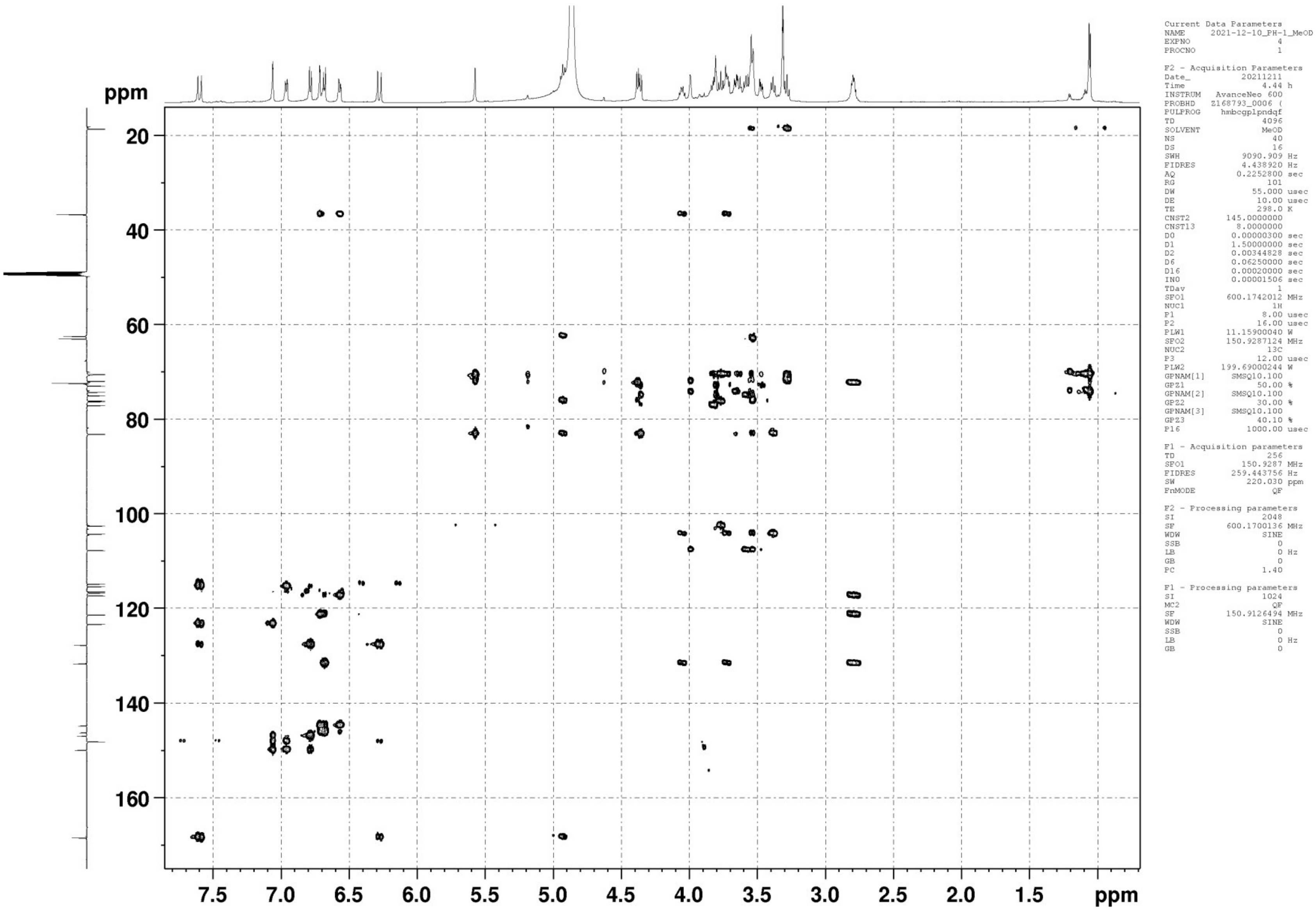

Figure S16: ^{1}H-^{13}C HMBC spectrum of **PH5** in methanol-d_4 at 25 °C

Acquisition Time (sec)	(0.1741, 0.0435)
Comment	Z168793_0006 (CPP1.1 TCI 600S3 H&F-C/N-D-05 Z XT)
Frequency (MHz)	(600.1700, 600.1700)
Nucleus	(1H, 1H)
Origin	AvanceNeo 600
Original Points Count	(1024, 256)
Points Count	(2048, 1024)
Pulse Sequence	roesyphpr.2
Solvent	METHANOL-d4
Sweep Width (Hz)	(5879.48, 5876.61)

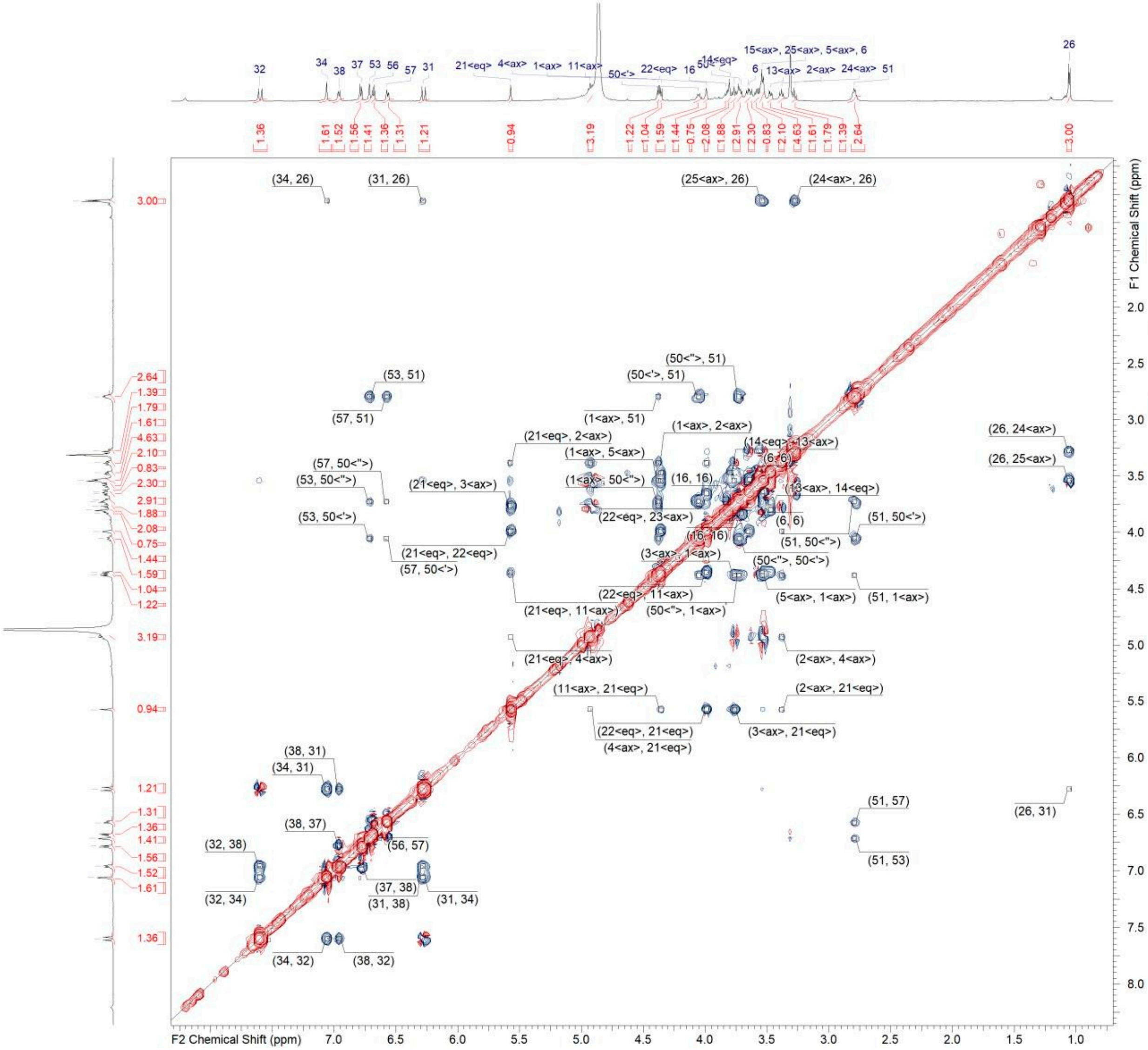

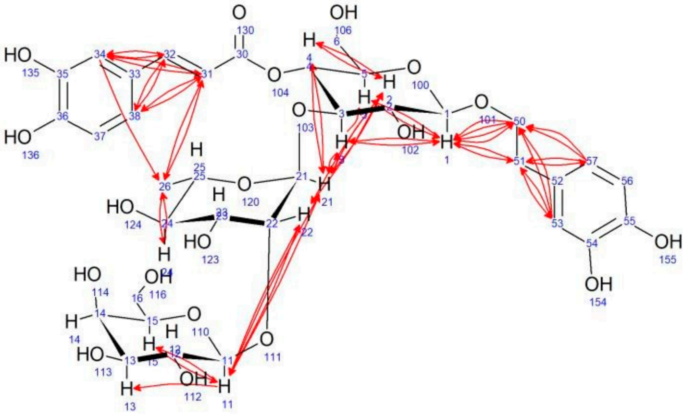

Figure S17: Fully assigned NOESY spectrum of **PH5** in methanol-d_4 at 25 °C

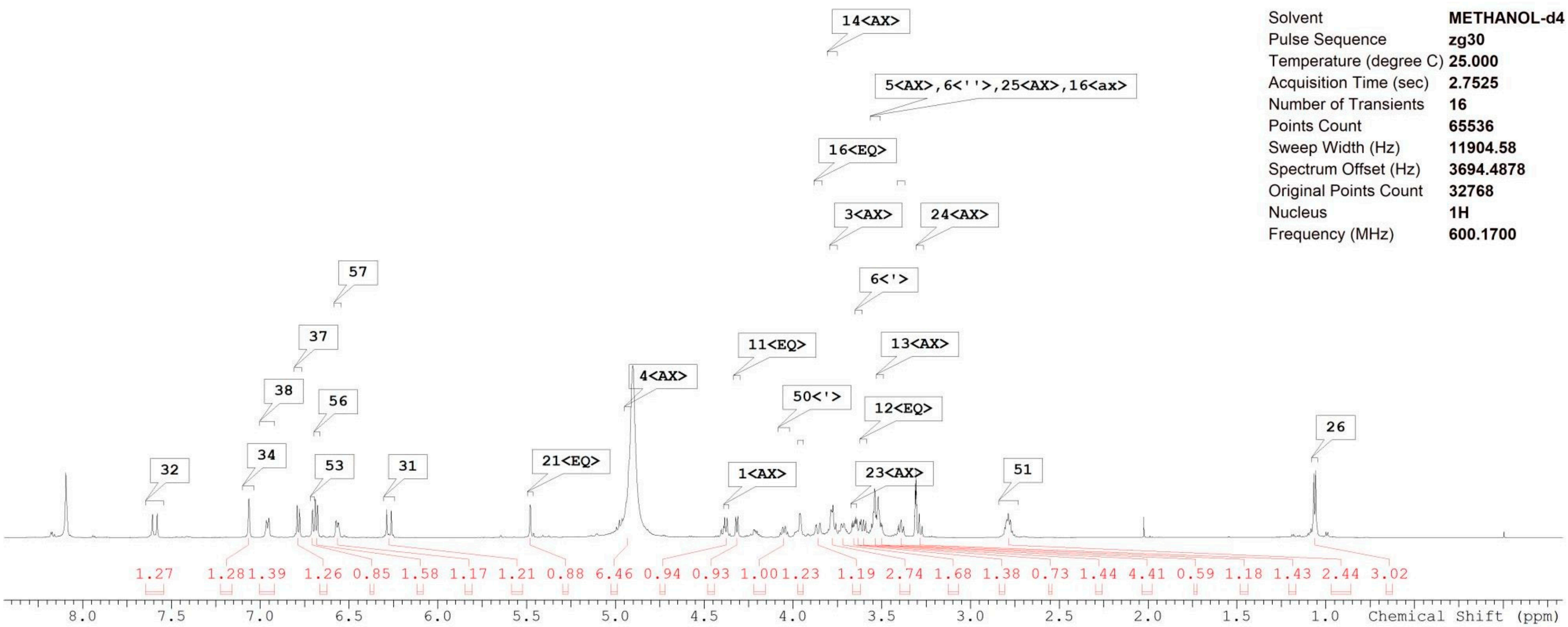

Shift1 (ppm)	H's	Type	J (Hz)	Atom1	Multiplet1
1.063	3.000	br d	6.2	26	M27
2.787	2.000	m	-	51	M26
3.290	1.000	t	9.6	24<AX>	M25
3.392	1.000	br t	9.3, 7.8	2<AX>	M24
3.510	1.000	br dd	9.0, 3.3	13<AX>	M23
3.540	4.000	m	-	5<AX>, 6<">, 25<AX>, 16<ax>	M22
3.605	1.000	br dd	9.0, 7.4	12<EQ>	M21
3.633	1.000	br dd	13.6, 3.5	6<'>	M19
3.658	1.000	br dd	9.6, 3.3	23<AX>	M20
3.720	1.000	q	9.4	50<">	M18
3.775	1.000	br t	9.3	3<AX>	M16
3.783	1.000	br s	-	14<AX>	M17
3.860	1.000	br dd	12.8, 2.1	16<EQ>	M15
3.961	1.000	br dd	3.0, 1.5	22<EQ>	M14
4.052	1.000	q	8.4	50<'>	M13

Shift1 (ppm)	H's	Type	J (Hz)	Atom1	Multiplet1
4.317	1.000	d	7.3	11<EQ>	M12
4.379	1.000	br d	7.8	1<AX>	M11
4.925	1.000	br s	9.3	4<AX>	M10
5.482	1.000	s	-	21<EQ>	M09
6.276	1.000	d	15.8	31	M08
6.568	1.000	br dd	8.0, 1.8	57	M07
6.684	1.000	d	8.0	56	M06
6.707	1.000	d	1.6	53	M05
6.786	1.000	d	8.2	37	M04
6.958	1.000	br dd	8.1, 1.9	38	M03
7.065	1.000	s	-	34	M02
7.594	1.000	br d	15.8	32	M01

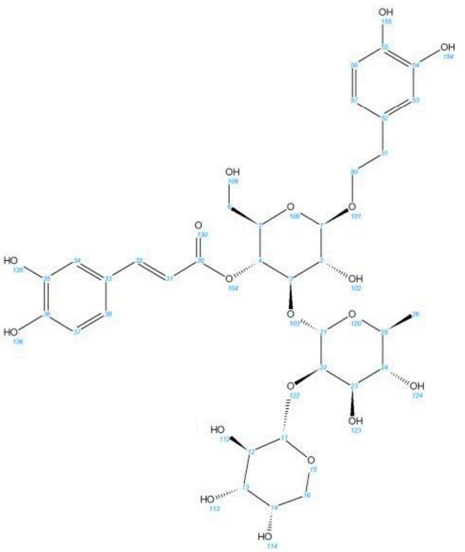

Figure S18: ^{1}H spectrum, structure, numbering and full assignment of **PH6** in methanol-d_4 at 25 °C

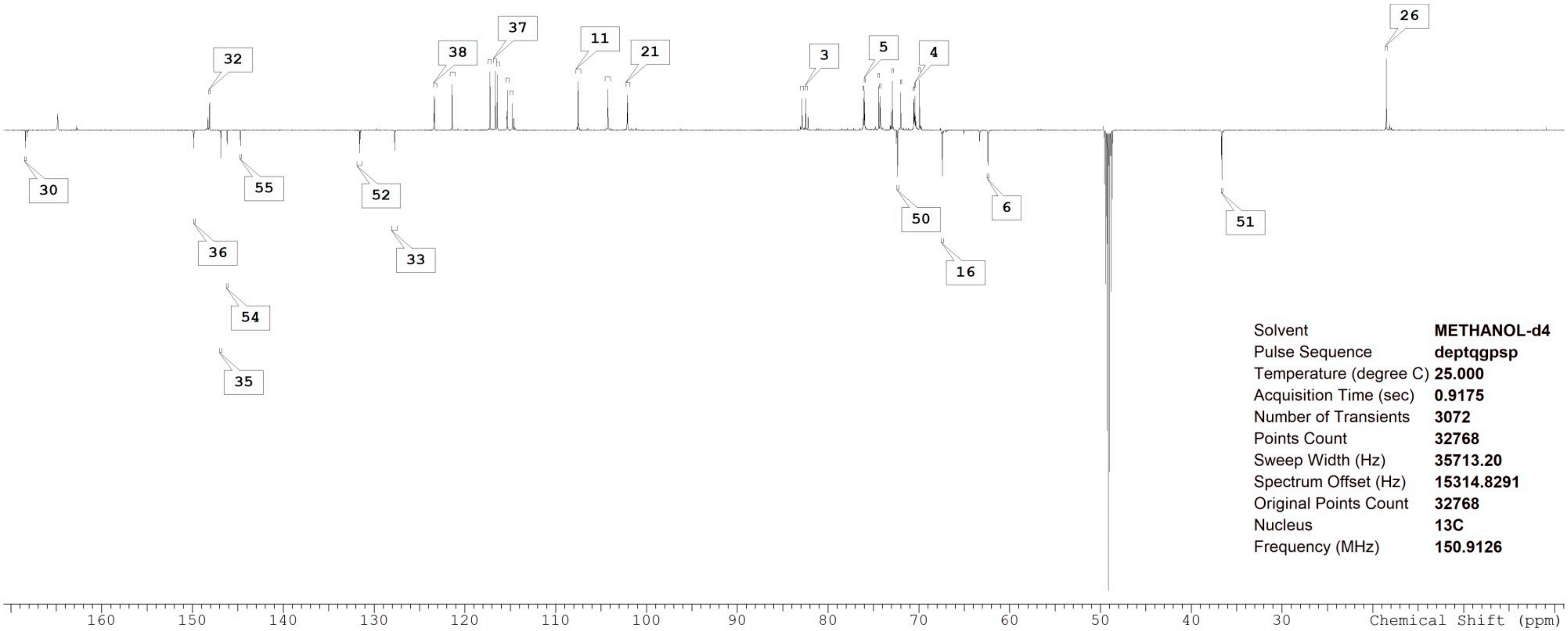

Shift1 (ppm)	C's	Type	Atom1	Multiplet1
18.546	1.000	s	26	M34
36.652	1.000	s	51	M33
62.413	1.000	s	6	M32
67.440	1.000	s	16	M31
69.953	1.000	s	14	M30
70.451	1.000	s	4	M29
70.574	1.000	s	25	M28
72.019	1.000	s	23	M27
72.286	1.000	m	50	M26
72.936	1.000	s	12	M25
74.294	1.000	s	24	M24
74.438	1.000	s	13	M23
76.034	1.000	s	5	M22
76.114	1.000	s	2	M21
82.484	1.000	s	3	M20

Shift1 (ppm)	C's	Type	Atom1	Multiplet1
82.888	1.000	s	22	M19
102.106	1.000	s	21	M18
104.258	1.000	s	1	M17
107.552	1.000	s	11	M16
114.781	1.000	s	31	M15
115.352	1.000	s	34	M14
116.442	1.000	s	56	M13
116.659	1.000	s	37	M12
117.258	1.000	s	53	M11
121.418	1.000	s	57	M10
123.375	1.000	s	38	M09
127.752	1.000	s	33	M08
131.616	1.000	s	52	M07
144.731	1.000	s	55	M05
146.190	1.000	s	54	M04

Shift1 (ppm)	C's	Type	Atom1	Multiplet1
146.898	1.000	s	35	M03
148.133	1.000	s	32	M06
149.859	1.000	s	36	M02
168.420	1.000	s	30	M01

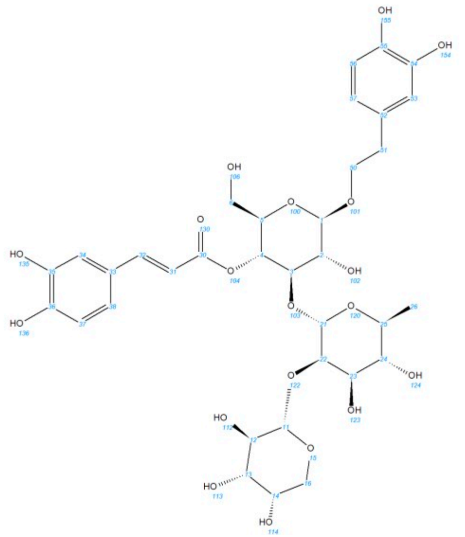

Figure S19: ^{13}C spectrum, structure, numbering and full assignment of **PH6** in methanol-d$_4$ at 25 °C

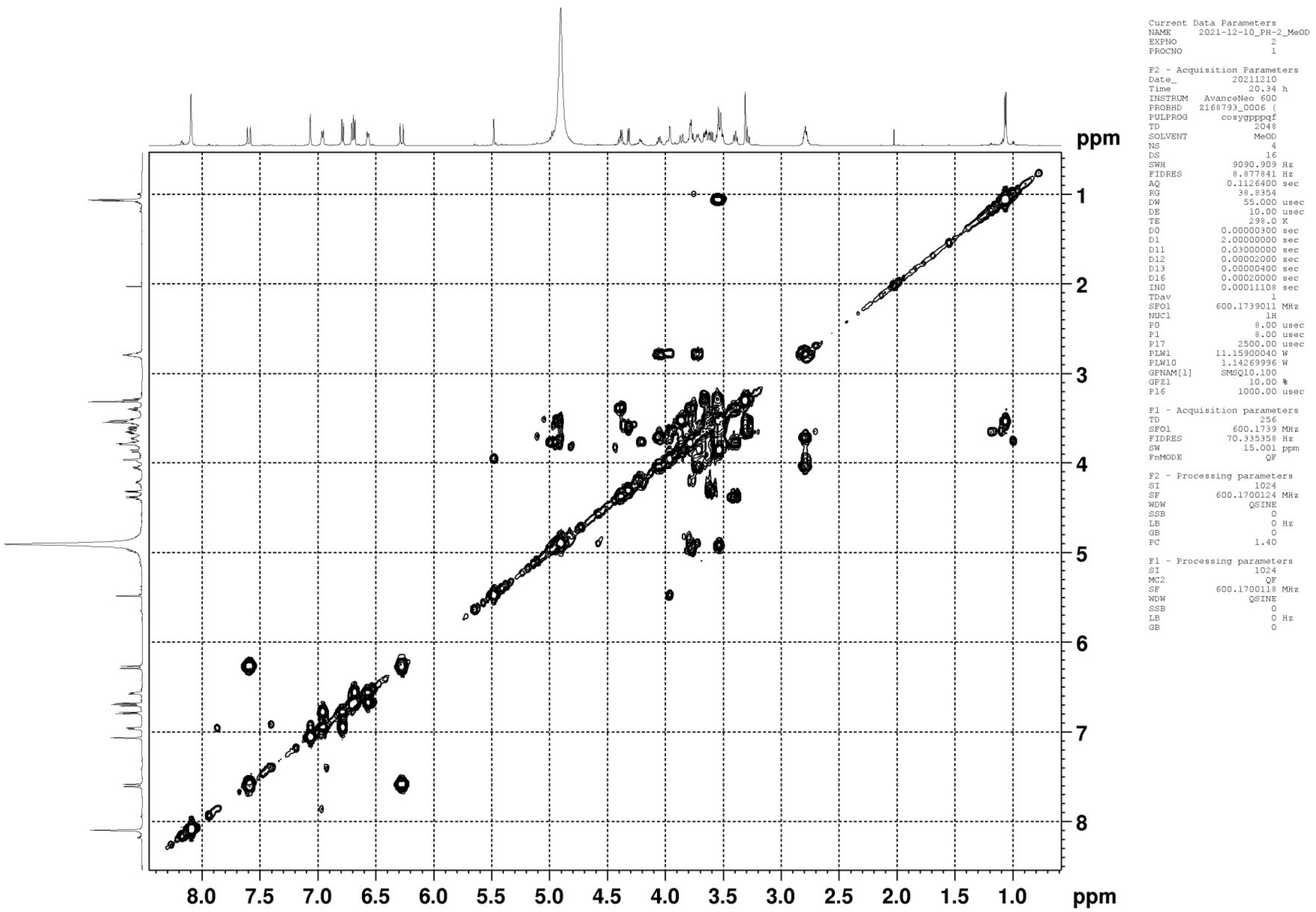

Figure S20: COSY spectrum of **PH6** in methanol-d_4 at 25 °C

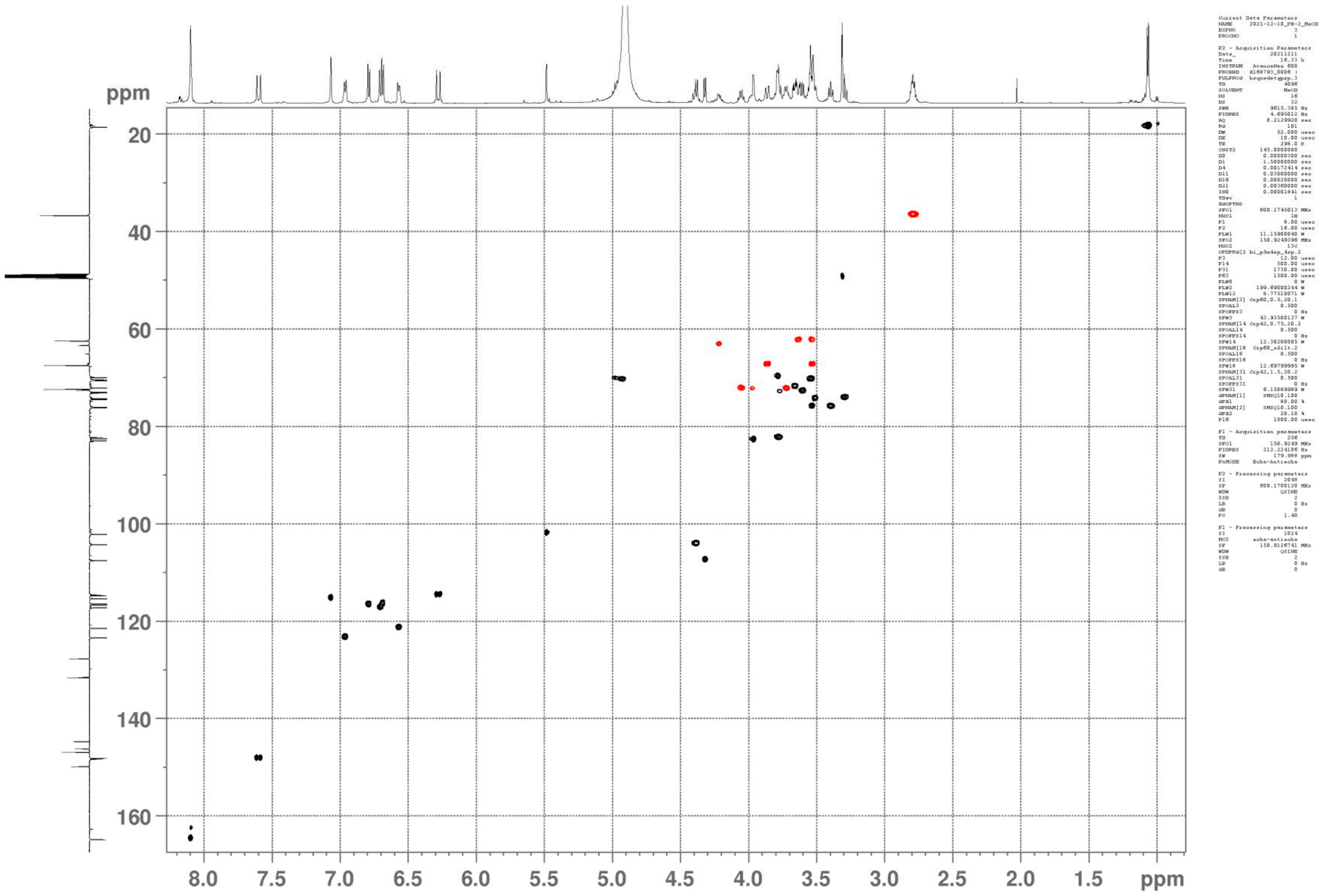

Figure S21: ^{1}H-^{13}C HSQC spectrum of **PH6** in methanol-d_4 at 25 °C

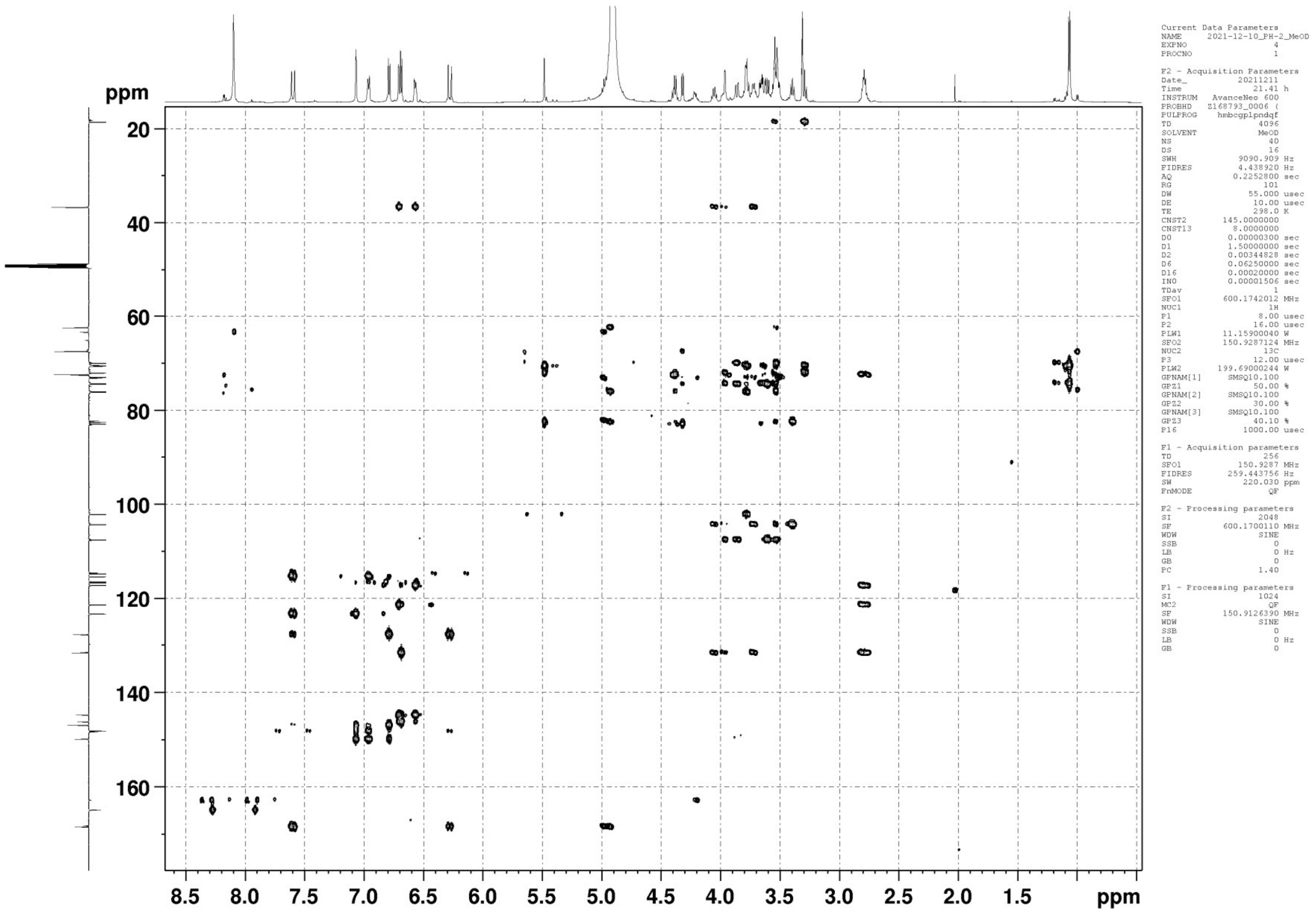

Figure S22: ^{1}H-^{13}C HMBC spectrum of **PH6** in methanol-d$_4$ at 25 °C

**Acquisition Time (sec)**	(0.0860, 0.0213)
**Comment**	Z168793_0006 (CPP1.1 TCI 600S3 H&F-C/N-D-05 Z XT)
**Frequency (MHz)**	(600.1700, 600.1700)
**Nucleus**	(1H, 1H)
**Origin**	AvanceNeo 600
**Original Points Count**	(1024, 256)
**Points Count**	(2048, 1024)
**Pulse Sequence**	roesyphpr.2
**Solvent**	METHANOL-d4
**Sweep Width (Hz)**	(11898.95, 11991.28)

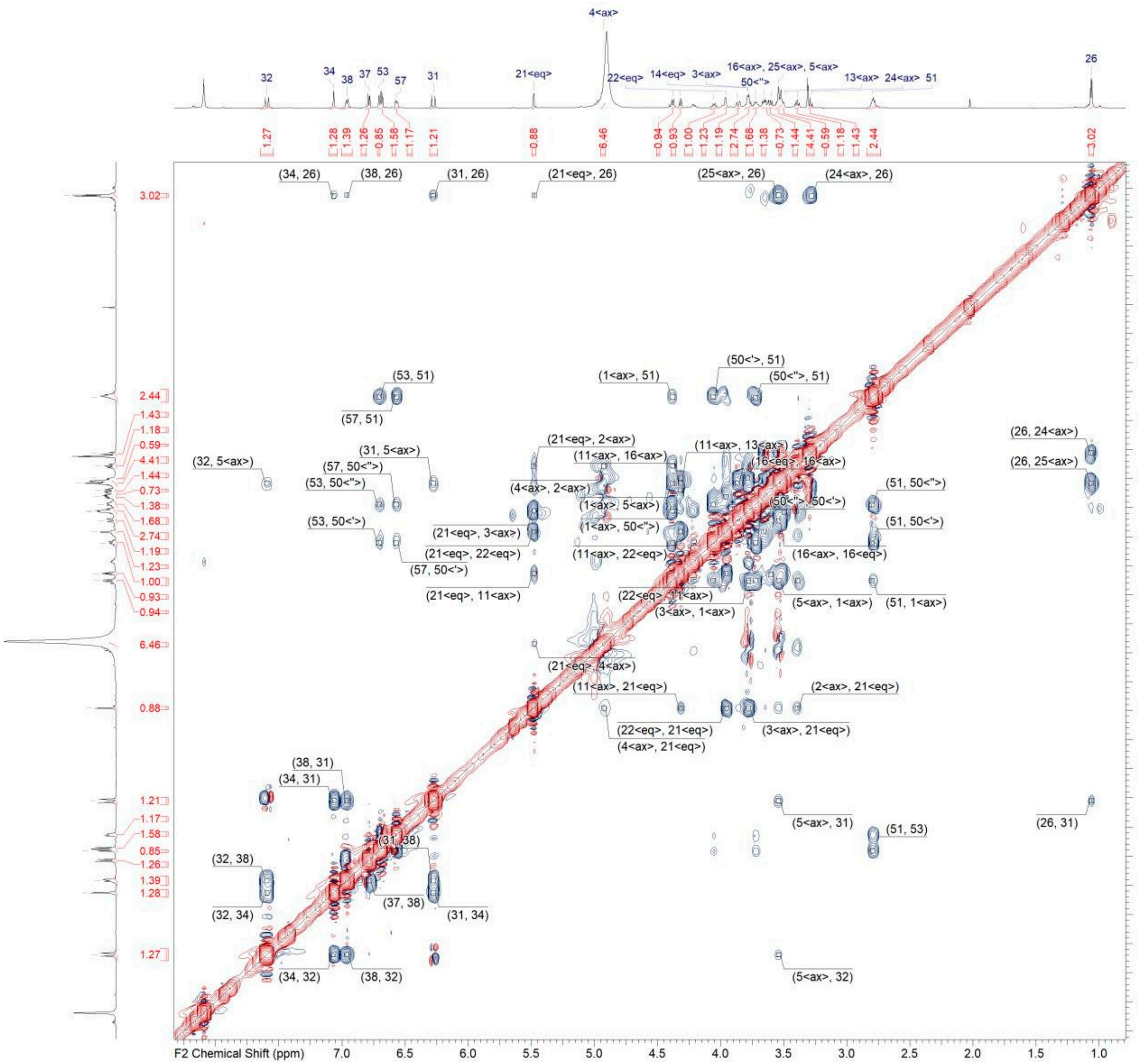

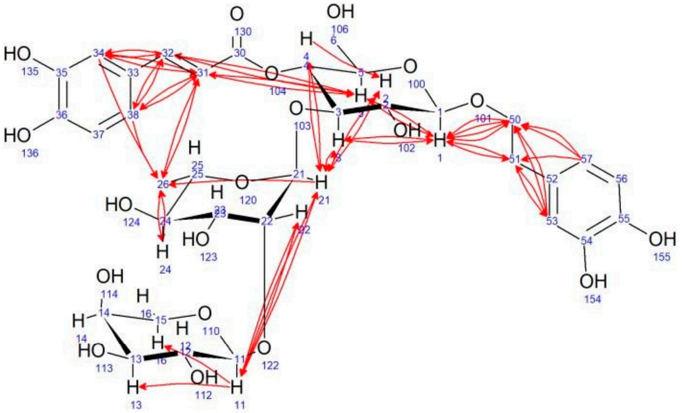

Figure S23: Fully assigned NOESY spectrum of **PH6** in methanol-d_4 at 25 °C

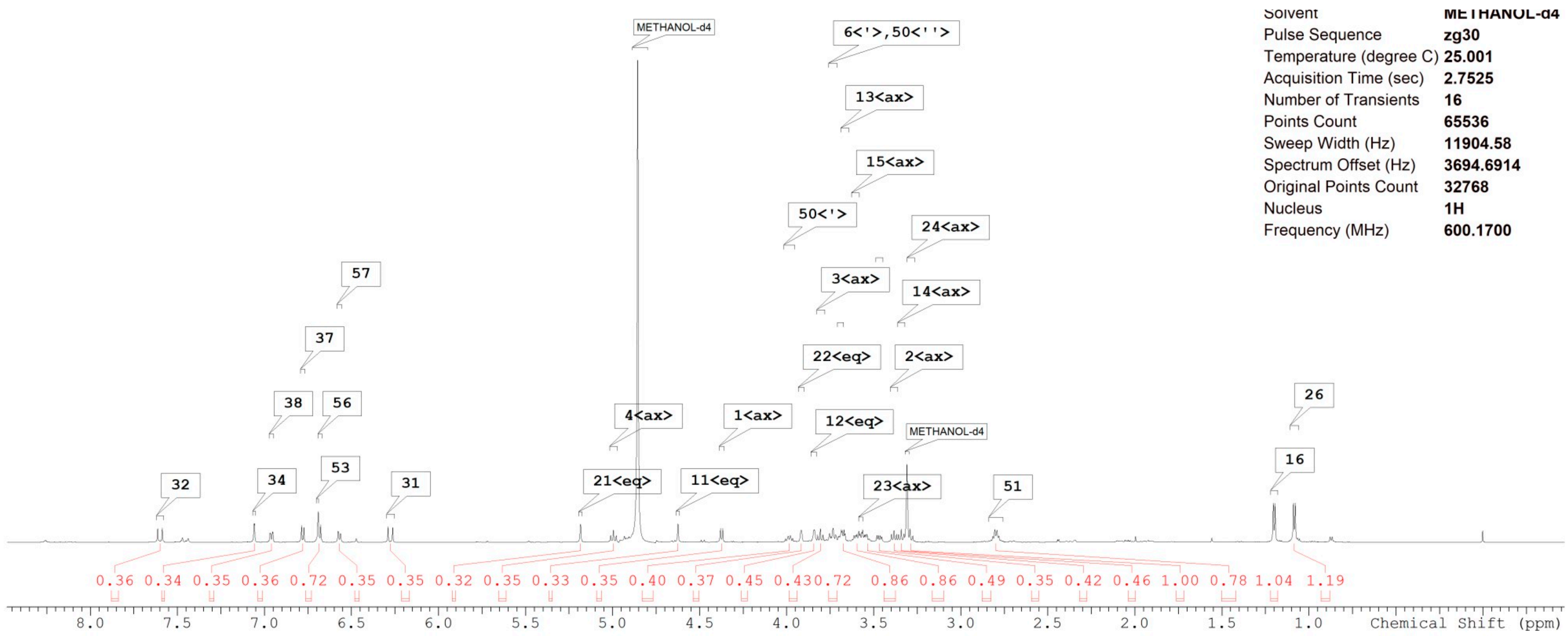

Shift1 (ppm)	H's	Type	J (Hz)	Connected Atoms	Atom1	Multiplet1	Shift1 (ppm)	H's	Type	J (Hz)	Connected Atoms	Atom1	Multiplet1
1.085	3.000	d	6.2	16	26	M02	3.917	1.000	dd	3.0, 1.5	-	22<eq>	M10
1.201	3.000	d	6.2	26	16	M03	3.986	1.000	ddd	9.5, 8.2, 6.9	-	50<'>	M13
2.799	2.000	br s	-	-	51	M04	4.375	1.000	d	8.0	57	1<ax>	M11
3.292	1.000	t	9.4	-	24<ax>	M05	4.626	1.000	d	1.2	-	11<eq>	M12
3.343	1.000	br t	9.5	-	14<ax>	M06	4.996	1.000	t	9.6	-	4<ax>	M14
3.384	1.000	t	8.5	-	2<ax>	M28	5.185	1.000	d	1.1	-	21<eq>	M15
3.468	1.000	dd	11.4, 5.4	-	6<">	M27	6.278	1.000	d	16.0	-	31	M17
3.552	1.000	dq	9.3, 6.2	-	25<ax>	M07	6.571	1.000	dd	8.0, 1.6	1<ax>, 38	57	M18
3.575	1.000	dd	8.7, 2.5	-	23<ax>	M29	6.684	1.000	d	7.9	-	56	M19
3.601	1.000	dq	9.3, 6.2	-	15<ax>	M30	6.693	1.000	d	1.6	-	53	M24
3.677	1.000	br dd	9.4, 3.5	-	13<ax>	M08	6.781	1.000	d	8.2	38	37	M20
3.691	1.000	ddd	9.6, 4.4, 2.2	-	5<ax>	M26	6.960	1.000	dd	8.2, 1.8	37, 57	38	M21
3.737	2.000	m	-	-	6<'>, 50<">	M25	7.059	1.000	d	1.8	-	34	M22
3.807	1.000	t	9.2	-	3<ax>	M09	7.601	1.000	d	15.8	-	32	M23
3.843	1.000	dd	3.2, 1.5	-	12<eq>	M16							

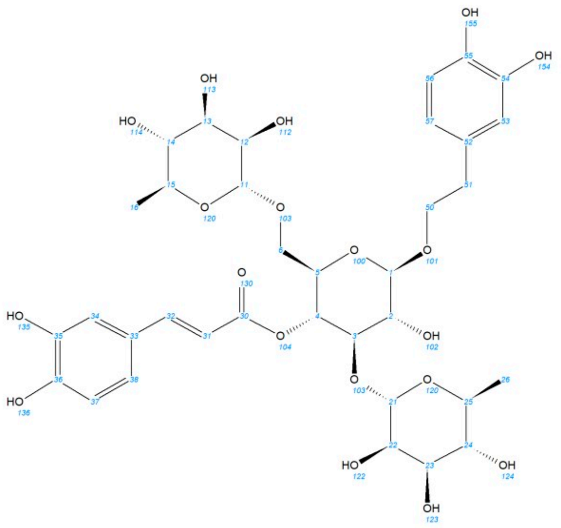

Figure S24: ^{1}H spectrum, structure, numbering and full assignment of **PH7** in methanol-d$_4$ at 25 °C

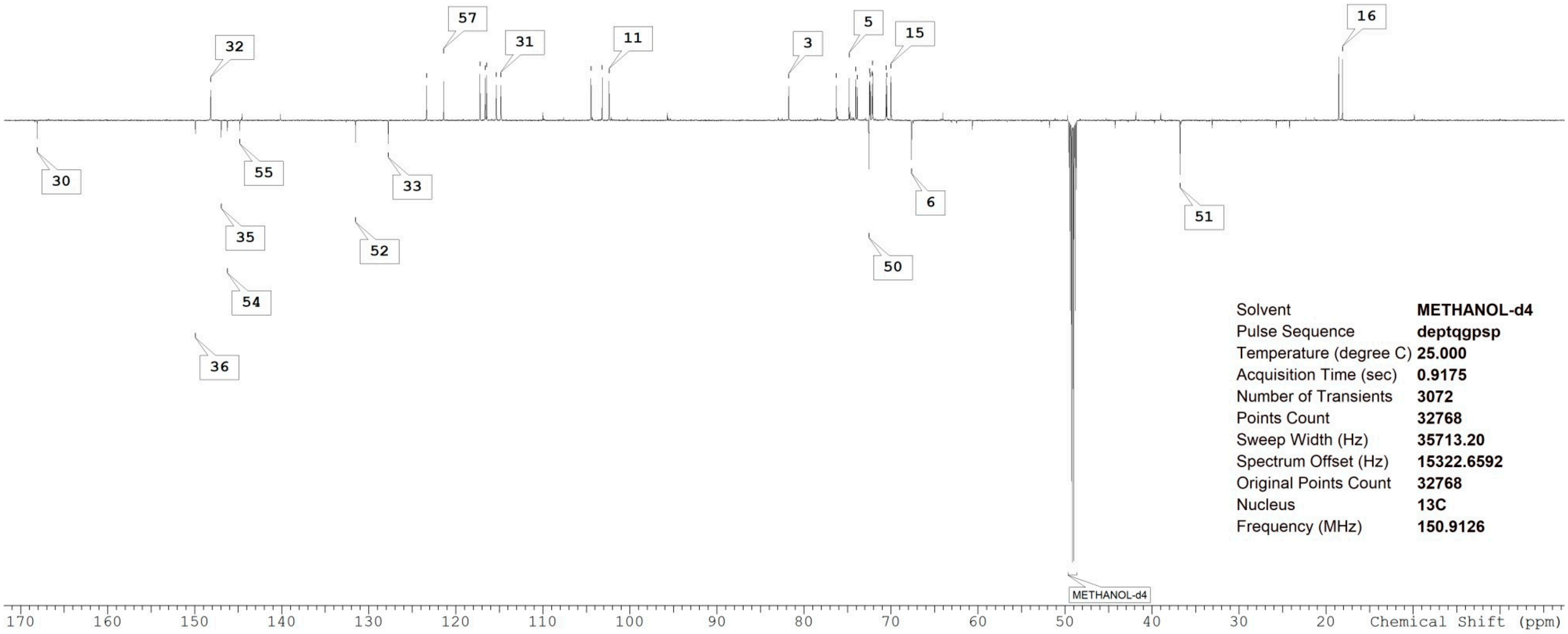

Shift1 (ppm)	C's	Type	Atom1	Multiplet1
18.150	1.000	s	16	M01
18.576	1.000	s	26	M02
36.805	1.000	s	51	M07
67.665	1.000	s	6	M11
70.019	1.000	s	15	M12
70.489	1.000	s	4	M13
70.576	1.000	s	25	M14
72.128	1.000	s	12	M15
72.186	1.000	s	23	M16
72.410	1.000	s	13	M17
72.475	1.000	s	22	M18
72.569	1.000	s	50	M19
73.891	1.000	s	24	M20
74.071	1.000	s	14	M21
74.837	1.000	s	5	M22
76.296	1.000	s	2	M23
81.763	1.000	s	3	M24
102.404	1.000	s	11	M25
103.205	1.000	s	21	M26
104.484	1.000	s	1	M27
114.840	1.000	s	31	M28
115.367	1.000	s	34	M29
116.494	1.000	s	56	M30
116.646	1.000	s	37	M31
117.238	1.000	s	53	M32
121.412	1.000	s	57	M33
123.377	1.000	s	38	M34
127.789	1.000	s	33	M35
131.531	1.000	s	52	M36
144.841	1.000	s	55	M37
146.264	1.000	s	54	M38
146.971	1.000	s	35	M39
148.192	1.000	s	32	M40
149.933	1.000	s	36	M41
168.125	1.000	s	30	M42

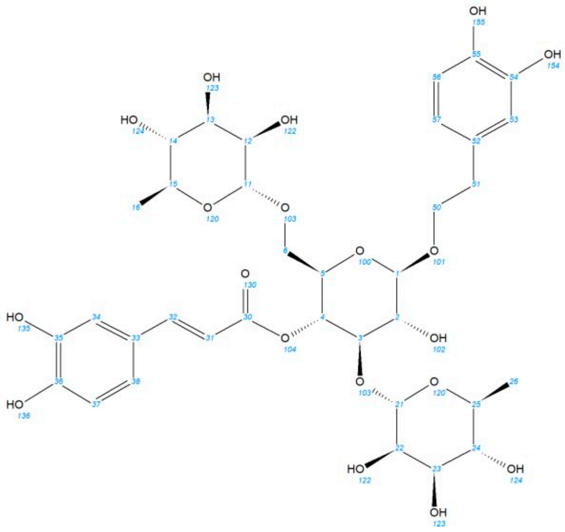

Figure S25: ^{13}C spectrum, structure, numbering and full assignment of **PH7** in methanol-d_4 at 25 °C

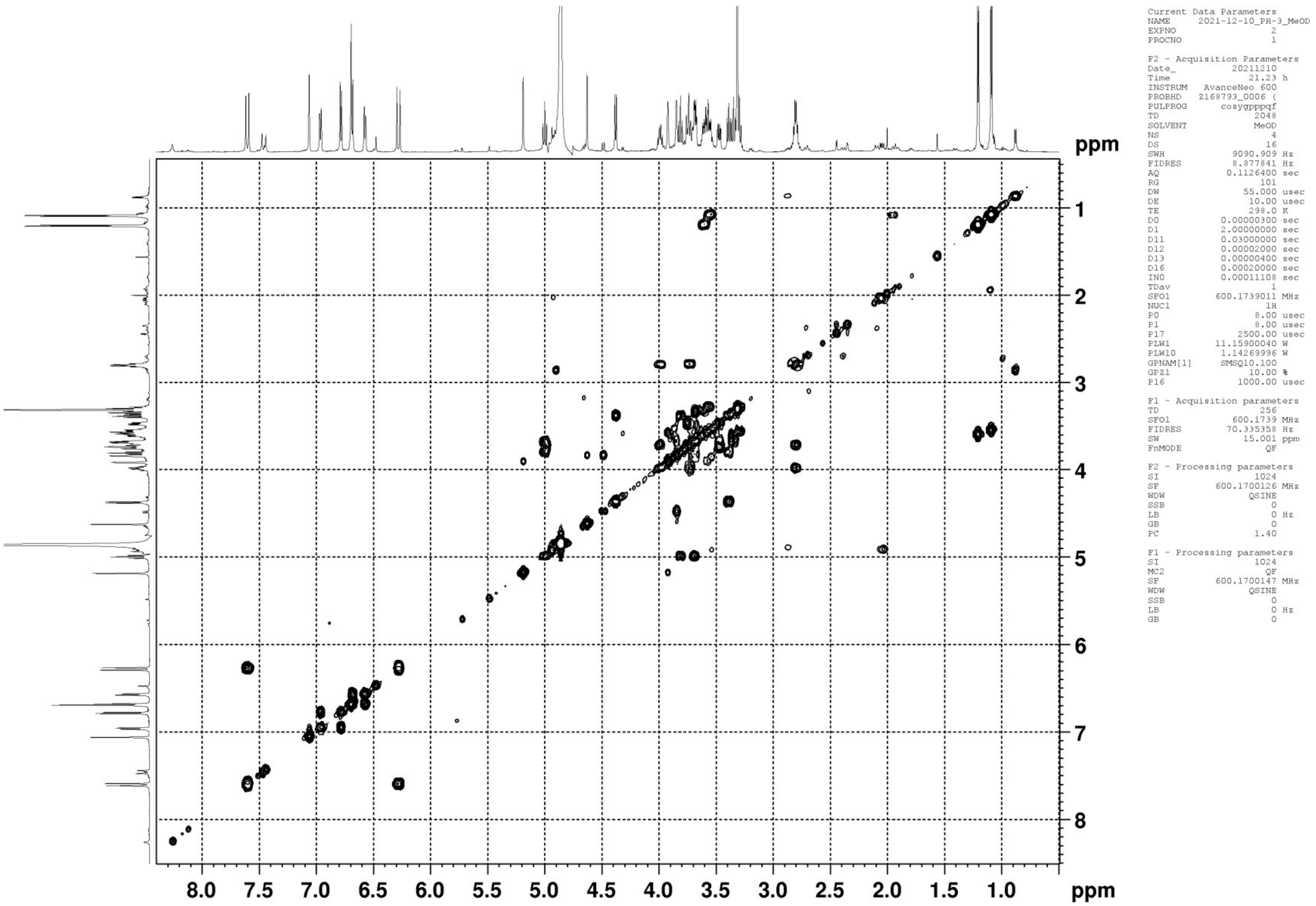

Figure S26: COSY spectrum of **PH7** in methanol-d_4 at 25 °C

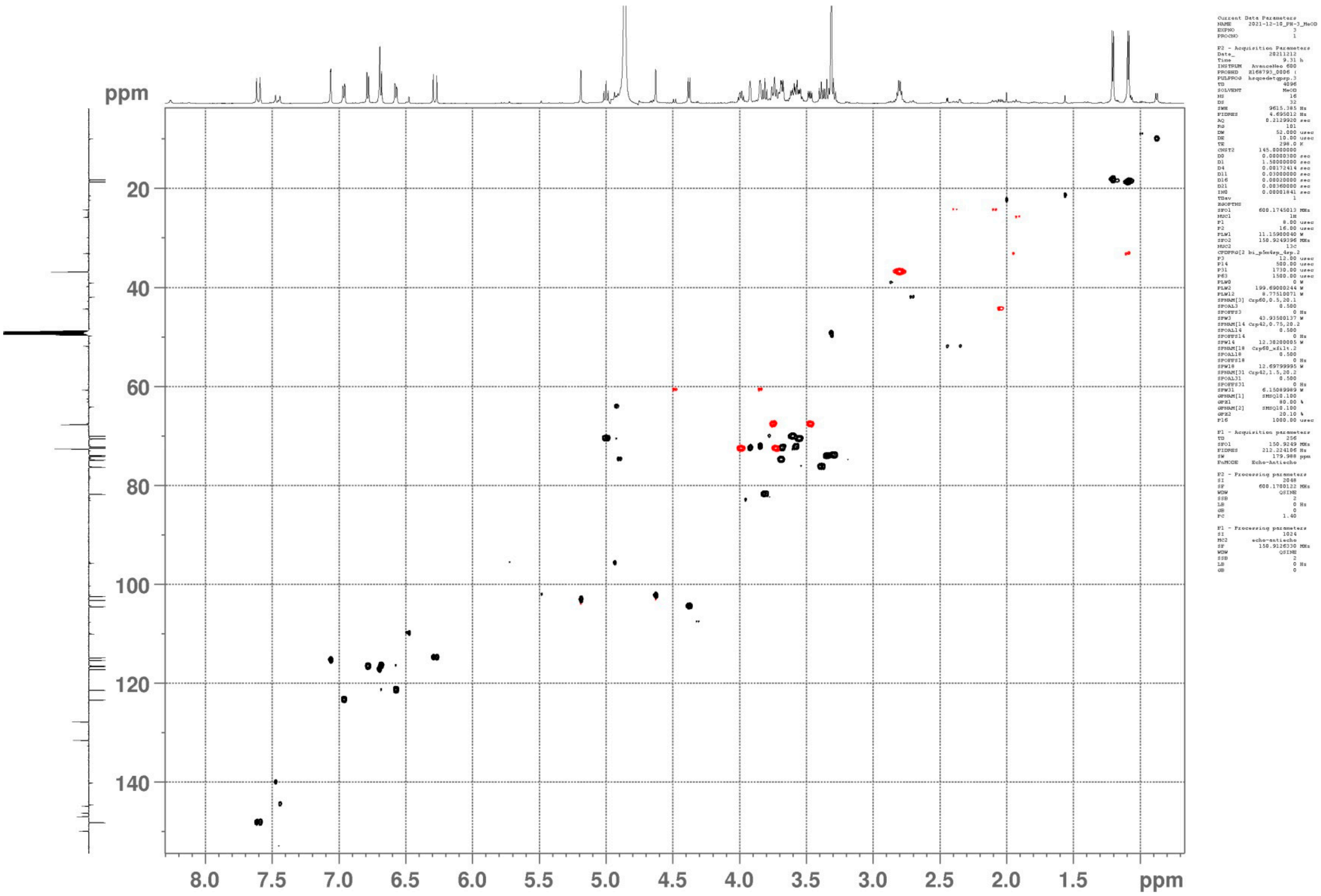

Figure S27: ^{1}H-^{13}C HSQC spectrum of **PH7** in methanol-d_4 at 25 °C

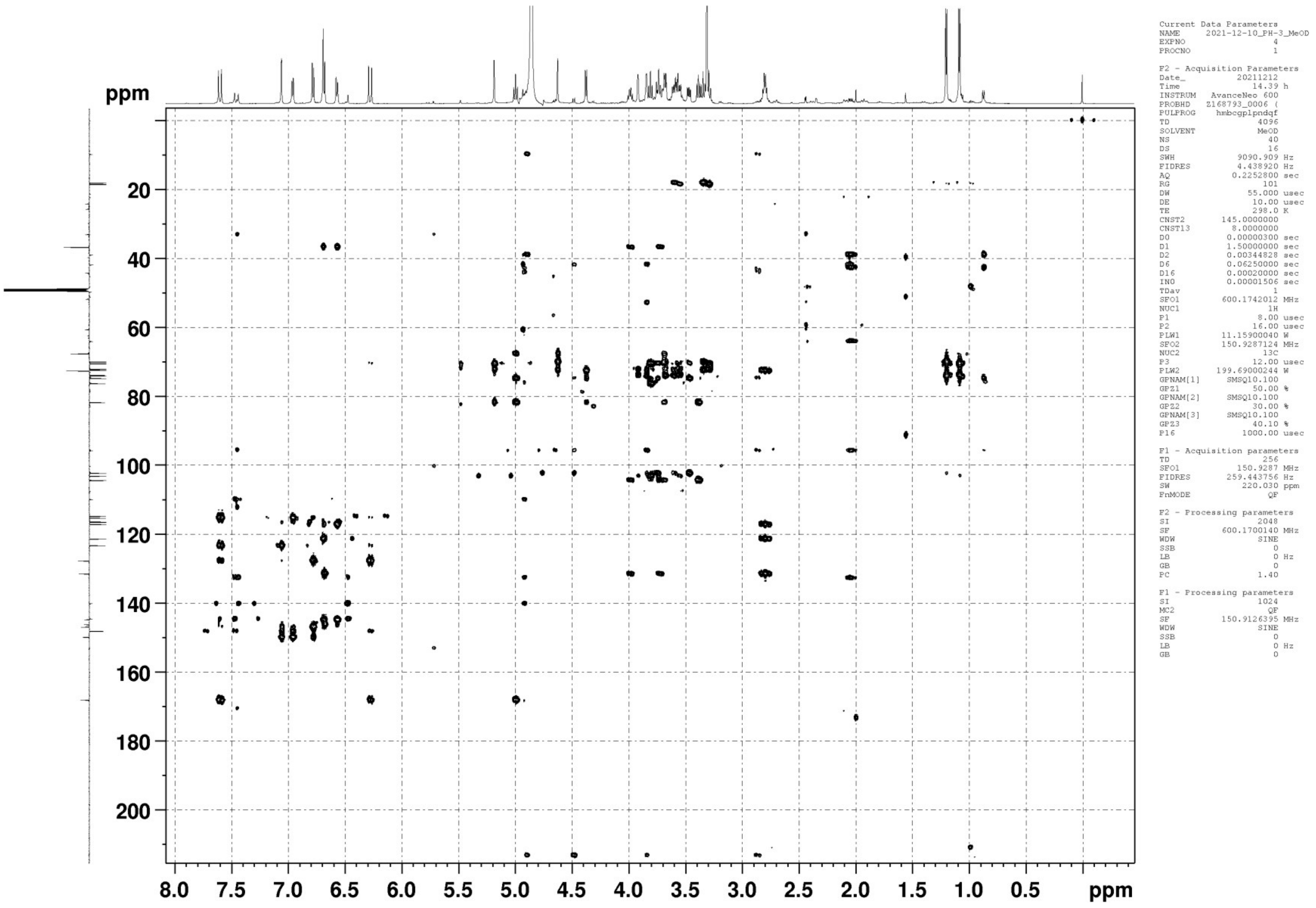

Figure S28: ^{1}H-^{13}C HMBC spectrum of **PH7** in methanol-d_4 at 25 °C

Acquisition Time (sec)	(0.0860, 0.0213)
Comment	Z168793_0006 (CPP1.1 TCI 600S3 H&F-C/N-D-05 Z XT)
Frequency (MHz)	(600.1700, 600.1700)
Nucleus	(1H, 1H)
Origin	AvanceNeo 600
Original Points Count	(1024, 256)
Points Count	(2048, 1024)
Pulse Sequence	noesygpphzs
Solvent	METHANOL-d4
Sweep Width (Hz)	(11898.95, 11991.28)

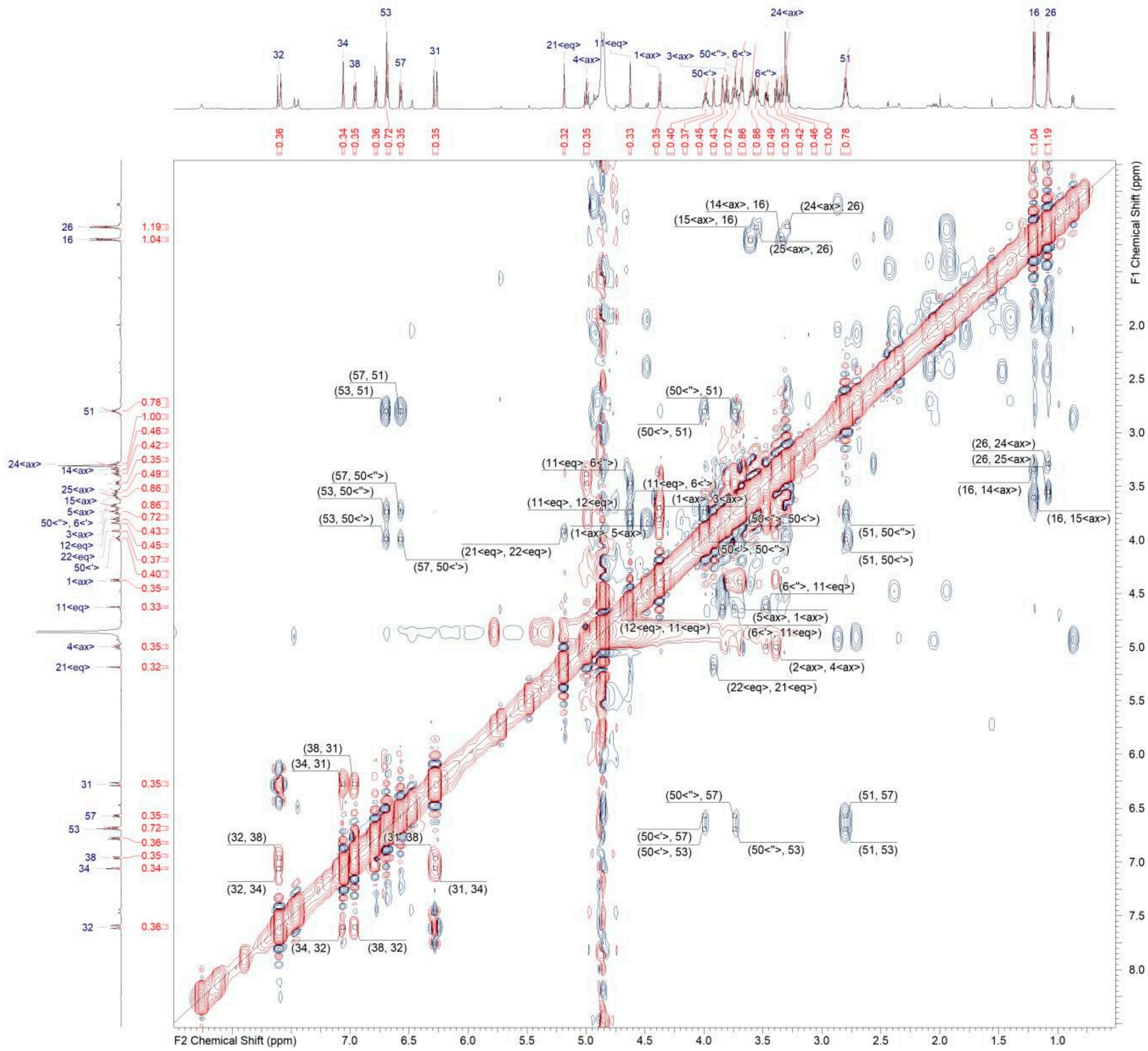

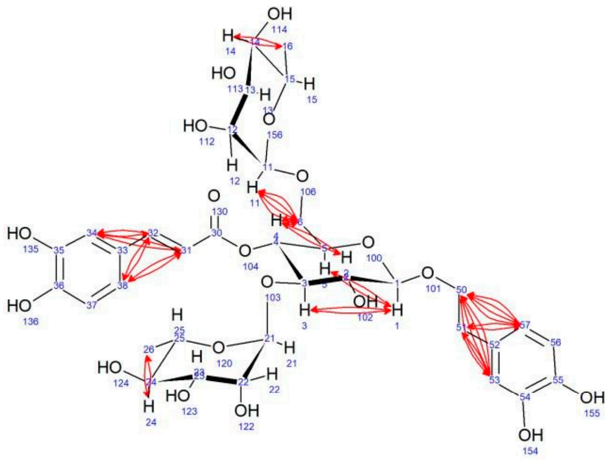

Figure S29: Fully assigned NOESY spectrum of **PH7** in methanol-d$_4$ at 25 °C

Table S1. Comparison of proton chemical shifts for all analyzed compounds in methanol- d_4 at 25 °C, the biggest differences marked with superscripted mark " * ".

Unit	Proton	Echinacoside	Verbascoside	PH5	PH6	PH7
β-D-glucopyranose	1<AX>	4.39	4.38	4.38	4.38	4.37
	2<AX>	3.40	3.39	3.38	3.39	3.38
	3<AX>	3.81	3.82	3.77	3.78	3.81
	4<AX>	5.01	4.92*	4.93*	4.93*	5.00
	5<AX>	3.77	3.54*	3.54*	3.54*	3.69
	6<'>	3.94	3.62*	3.64*	3.63*	3.47*
	6<">	3.64	3.52*	3.54*	3.54*	3.74*
Second sugar	11	4.30	n/a	4.36	4.32	4.63*
	12	3.21	n/a	3.58*	3.61*	3.84*
	13	3.34	n/a	3.47*	3.51*	3.68*
	14	3.28	n/a	3.81*	3.78*	3.34
	15	3.22	n/a	3.54*	n/a	3.60
	16<'>	3.83	n/a	3.82	3.86*	1.20
	16<">	3.65	n/a	3.72	3.54*	n/a
α-L-rhamnose	21<EQ>	5.18	5.19	5.57*	5.48*	5.18
	22<EQ>	3.92	3.92	3.99*	3.96*	3.92
	23<AX>	3.57	3.57	3.66*	3.66*	3.58
	24<AX>	3.29	3.29	3.28	3.29	3.29
	25<AX>	3.55	3.57	3.54	3.54	3.55
	26	1.09	1.09	1.05	1.06	1.08
Caffeic acid	31	6.28	6.28	6.28	6.28	6.28
	32	7.60	7.60	7.60	7.59	7.60
	34	7.06	7.06	7.06	7.07	7.06
	37	6.78	6.78	6.78	6.79	6.78
	38	6.96	6.96	6.96	6.96	6.96
Hydroxytyrosol	50<'>	4.04	4.05	4.05	4.05	3.99
	50<">	3.73	3.72	3.72	3.72	3.74
	51	2.80	2.79	2.79	2.79	2.80
	53	6.71	6.70	6.71	6.71	6.69
	56	6.68	6.68	6.68	6.68	6.68
	57	6.58	6.57	6.57	6.57	6.57

Table S2. Comparison of carbon chemical shifts for all analyzed compounds in methanol- d_4 at 25 °C, the biggest differences marked with superscripted mark " * ".

Unit	Carbon	Echinacoside	Verbascoside	PH5	PH6	PH7
**β-D-glucopyranose**	1	104.3	104.3	104.2	104.3	104.5
	2	76.3	76.3	76.3	76.1	76.3
	3	81.8	81.8	83.1	82.5	81.8
	4	70.7	70.7	70.5	70.5	70.5
	5	74.8	76.2	76.1	76.0	74.8
	6	69.5	62.5*	62.4*	62.4*	67.7*
**second sugar**	11	104.8	n/a	107.7*	107.6*	102.4*
	12	75.2	n/a	73.0*	72.9*	72.1
	13	77.9	n/a	75.0*	74.4*	72.4
	14	71.6	n/a	70.5*	70.0*	74.1
	15	78.0	n/a	77.1		70.0
	16	62.7	n/a	63.0	67.4*	18.2
**α-L-rhamnose**	21	103.2	103.2	102.5	102.1	103.2
	22	72.5	72.5	83.2*	82.9*	72.5
	23	72.2	72.2	71.9	72.0	72.2
	24	73.9	73.9	74.2	74.3	73.9
	25	70.6	70.6	70.5	70.6	70.6
	26	18.6	18.6	18.6	18.5	18.6
**caffeic acid**	30	168.6	168.4	168.4	168.4	168.1
	31	114.8	114.8	114.8	114.8	114.8
	32	148.4	148.2	148.1	148.1	148.2
	33	127.8	127.8	127.8	127.8	127.8
	34	115.4	115.4	115.3	115.4	115.4
	35	147.0	146.3	147.0	146.9	147.0
	36	150.0	149.9	149.9	149.9	149.9
	37	116.7	116.6	116.7	116.7	116.6
	38	123.4	123.4	123.4	123.4	123.4
**hydroxytyrosol**	50	72.5	72.4	72.3	72.3	72.6
	51	36.7	36.7	36.6	36.7	36.8
	52	131.6	131.6	131.7	131.6	131.5
	53	117.3	117.2	117.3	117.3	117.2
	54	146.2	147.0	146.2	146.2	146.3
	55	144.8	144.8	144.8	144.7	144.8
	56	116.5	116.4	116.4	116.4	116.5
	57	121.5	121.4	121.4	121.4	121.4

Table S3. Comparison of coupling constants $^nJ_{H,H}$ (Hz) for all analyzed compounds in methanol-d4 at 25 °C, the biggest differences marked with superscripted mark " * ".

$^nJ_{H,H}$/Hz	Proton	Echinacoside	Verbascoside	PH5	PH6	PH7
β-D-glucopyranose	1, 2	7.8	7.8	8.0	7.8	8.0
	2, 3	9.3	9.2	9.1	9.3	9.2
	3, 4	9.6	9.5	9.3	9.3	9.6
	4, 5	9.6	9.5	9.3	9.3	9.6
	5, 6a	2.2	<1*	overlap	3.5	2.2
	5, 6b	5.5	5.8	overlap	overlap	5.5
	6a, 6b	11.4	10.2	12.4	13.6	11.4
second sugar	11, 12	7.6	n/a	7.6	7.3	1.5
	12, 13	9.3	n/a	9.6	9.0	3.2
	13, 14	9.1	n/a	3.4*	3.3*	9.5
	14, 15	9.0	n/a	overlap (ca 2-3 Hz)*	n/a	9.3
	15, 16a	2.3	n/a	7.3*	n/a	6.2
	15, 16b	5.6	n/a	overlap	n/a	n/a
	16a, 16b	11.9	n/a	11.4	12.8*	n/a
	14, 16a	n/a	n/a	n/a	2.1*	n/a
	14, 16b	n/a	n/a	n/a	overlap (ca 3-4 Hz)*	n/a
α-L-rhamnose	21, 22	1.6	1.6	1.3	1.5	1.5
	22, 23	3.3	3.2	3.1	3.3	3.0
	23, 24	9.5	9.6	9.6	9.6	9.5
	24, 25	9.6	9.6	9.6	9.6	9.3
	25, 26	6.2	6.2	6.0	6.2	6.2
caffeic acid	31, 32	15.8	15.8	15.8		15.8
	34, 38	2.0	2.0	1.8	1.9	1.8
	37, 38	8.3	8.2	8.2	8.2	8.2
hydroxytyrosol	50a, 50b	9.6	9.6	9.5	9.5	9.5
	50a, 51a<br>50a, 51b	7.0; 8.1	6.7; 8.3	6.7; 7.8	8.4<br>8.4	8.17; 6.9
	50b, 51a<br>50b, 51b	6.7; 8.2	6.8; 8.3	overlap<br>overlap	9.5<br>9.5	overlap<br>overlap
	53, 57	2.2	2.0	1.8	1.8	1.6
	56, 57	8.0	8.0	8.0	8.0	8.0
